# Supplementary material for: TRP14 is the rate-limiting enzyme for intracellular cystine reduction and regulates proteome cysteinylation
Source: EMBO J. 2024 May 29;43(13):12. doi: 10.1038/s44318-024-00117-1 (PMC11217419; doi:10.1038/s44318-024-00117-1)
Supplement: Supplementary file 1 — Appendix [file 44318_2024_117_MOESM1_ESM.pdf]

Table of content of the Appendix Figures and Tables

Page

Appendix Figure S1……………………………………………………….2

Appendix Figure S2……………………………………………………….3

Appendix Figure S3……………………………………………………….4

Appendix Figure S4……………………………………………………….5

Appendix Figure S5……………………………………………………….6

Appendix Figure S6……………………………………………………….7

Appendix Figure S7……………………………………………………….8

Appendix Figure S8……………………………………………………….9

Appendix Figure S9……………………………………………………...10

Appendix Table S1……………………………………………………...11

Appendix Table S2……………………………………………………...12

Appendix Table S3……………………………………………………...13

Appendix Table S4……………………………………………………...14

Appendix Table S5……………………………………………………...15

Appendix Table S6……………………………………………………...16

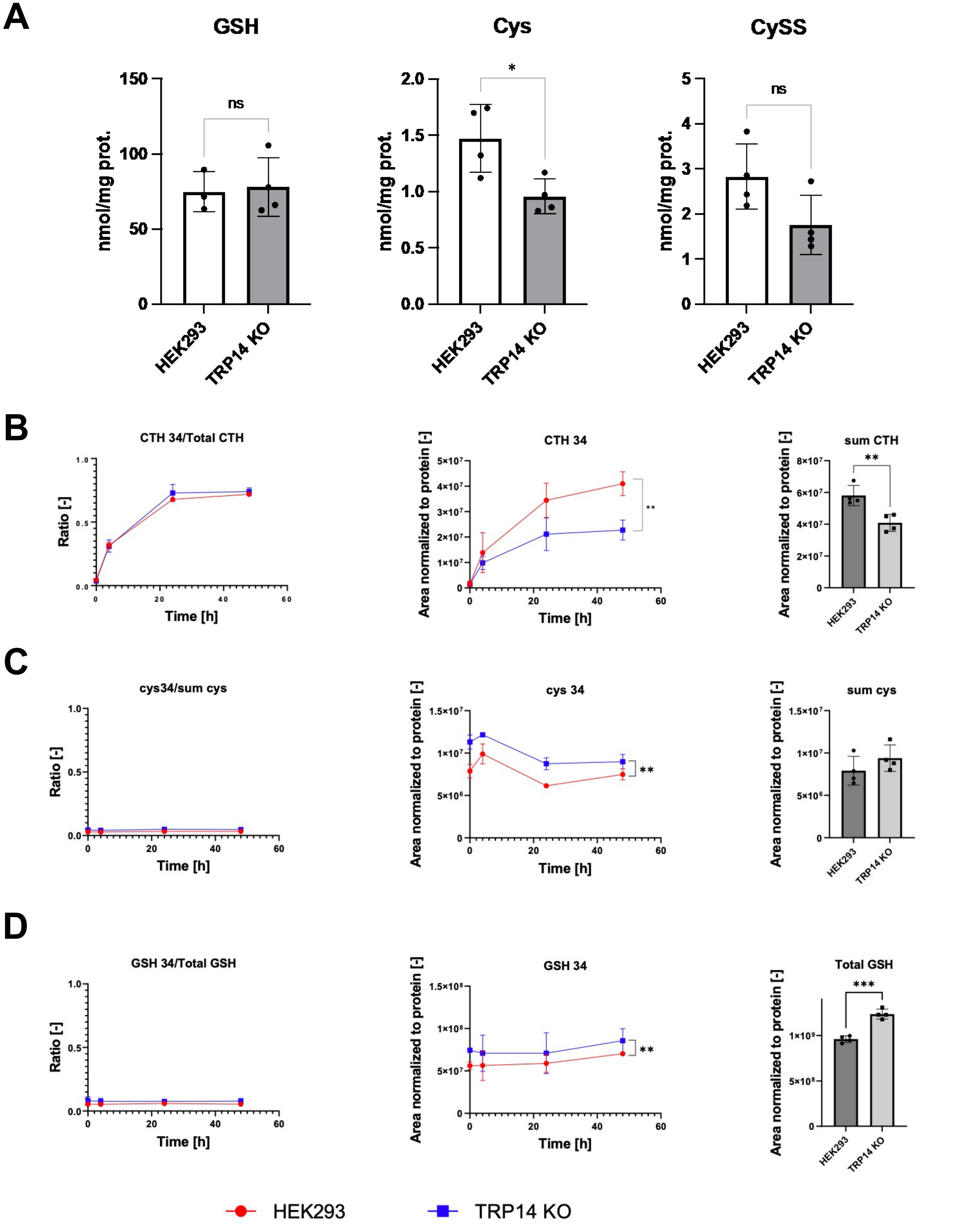

Appendix Figure S1. **A)** GSH, Cys, and CySS levels in HEK293 and TRP14 KO cells under normal culturing conditions (200 µM CySS in the culture medium) . **B-D)** Analysis of the flux through the transsulfuration pathway in HEK293 WT and TRP14 KO cells using heavy sulfur ([34S])-labeled methionine under normal culturing conditions. Ratio of total (left), steady-state levels of heavy (middle) and steady-state levels of total (right) cystathionine (**B**), cysteine (**C**), and glutathione (**D**) under normal culturing conditions (200 µM CySS in the culture medium) in HEK293 WT and TRP14 KO cells.

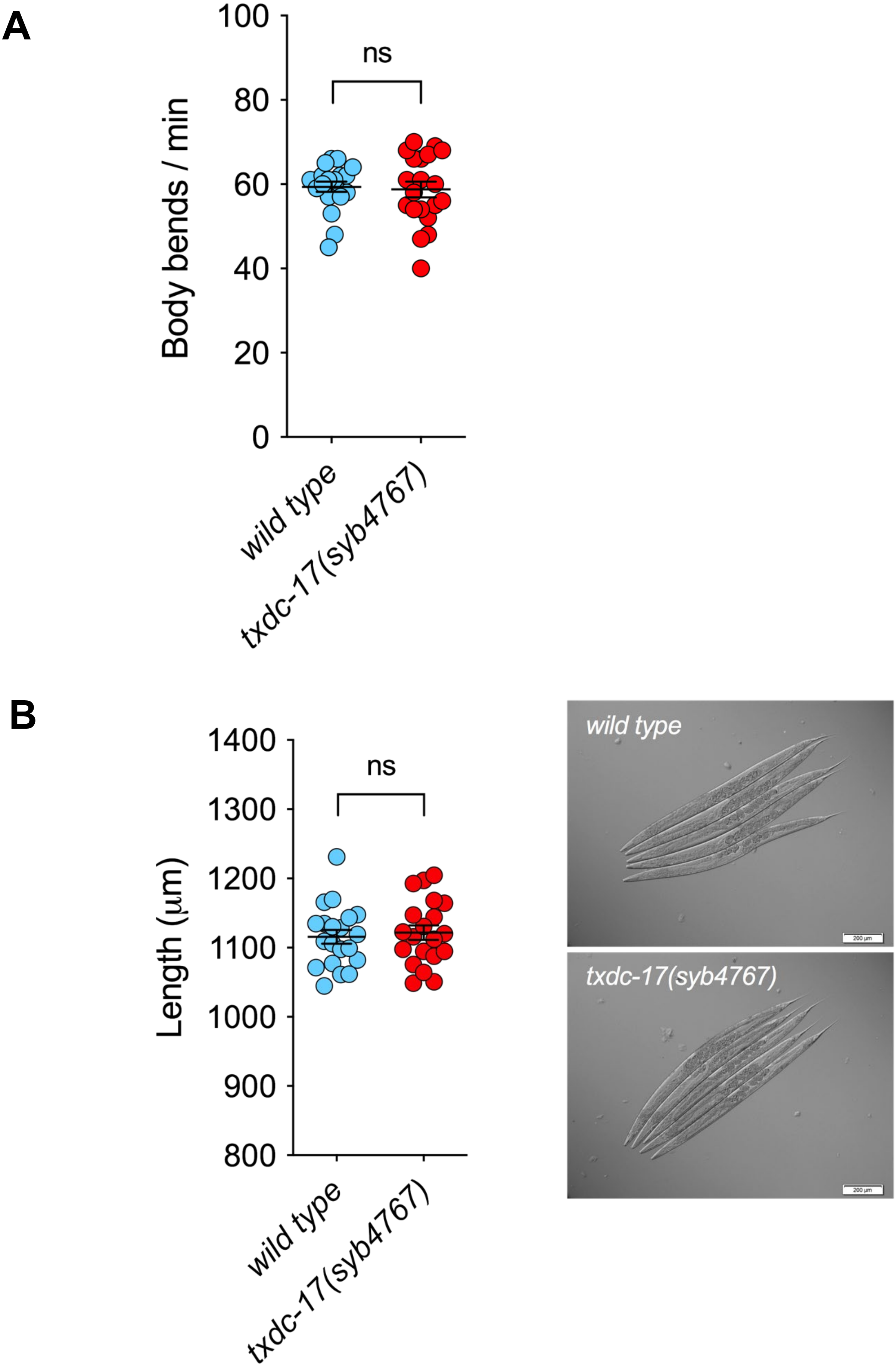

Appendix Figure S2. **A)** Motility (body bends per minute) of wild type and *txdc-17(syb4767)* worms. **B)** Length of *wild type* and *txdc-17(syb4767)* worms. A representative image corresponding to each genotype is shown.

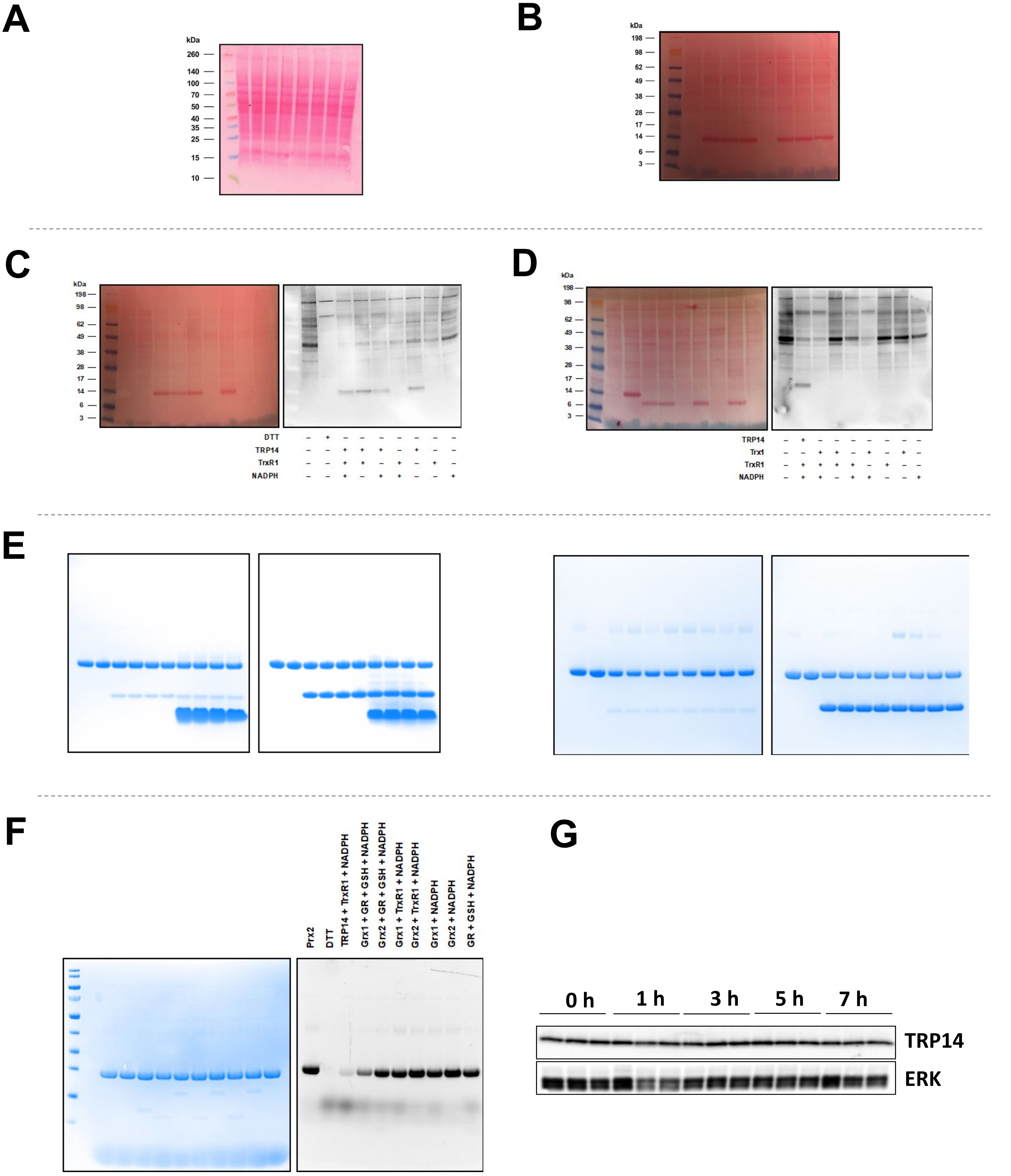

Appendix Figure S3. **A)** Ponceau staining of the membrane shown in Figure 5A. **B)** Ponceau staining of the membrane shown in Figure 5B. **C)** <u>Protein de-cysteinylation experiments on cysteinylated cell lysates: the TRP14 system</u>. Control experiments showed that all the components of the enzymatic system are required for an efficient reduction of the cysteinylated substrates by TRP14. A Ponceau staining is shown as a loading control. **D)** <u>Protein de-cysteinylation experiments on cysteinylated cell lysates: the Trx1 system</u>. All the components of the enzymatic system are required for an efficient reduction of the cysteinylated substrates by Trx1. A Ponceau staining is shown as a loading control. **E)** Coomassie staining (loading control) corresponding to the gels shown in Figures 5F and 5G. **F) Protein decysteinylation assays: the glutaredoxin 1 and 2 systems:** Effect of the different components of the glutaredoxin 1 and 2 systems on cysteinylated Prx2. The concentrations used in the experiments were as follows: 25 μM Prx2; 10 μM TRP14; 10 nM TrxR1; 10 μM Grx1; 10 μM Grx2; 10 nM GR; 1mM GSH; 1 mM NADPH. Incubation time: 15 min. **G)** Western blotting of TRP14 in the time course of acute pancreatitis with ERK as loading control.

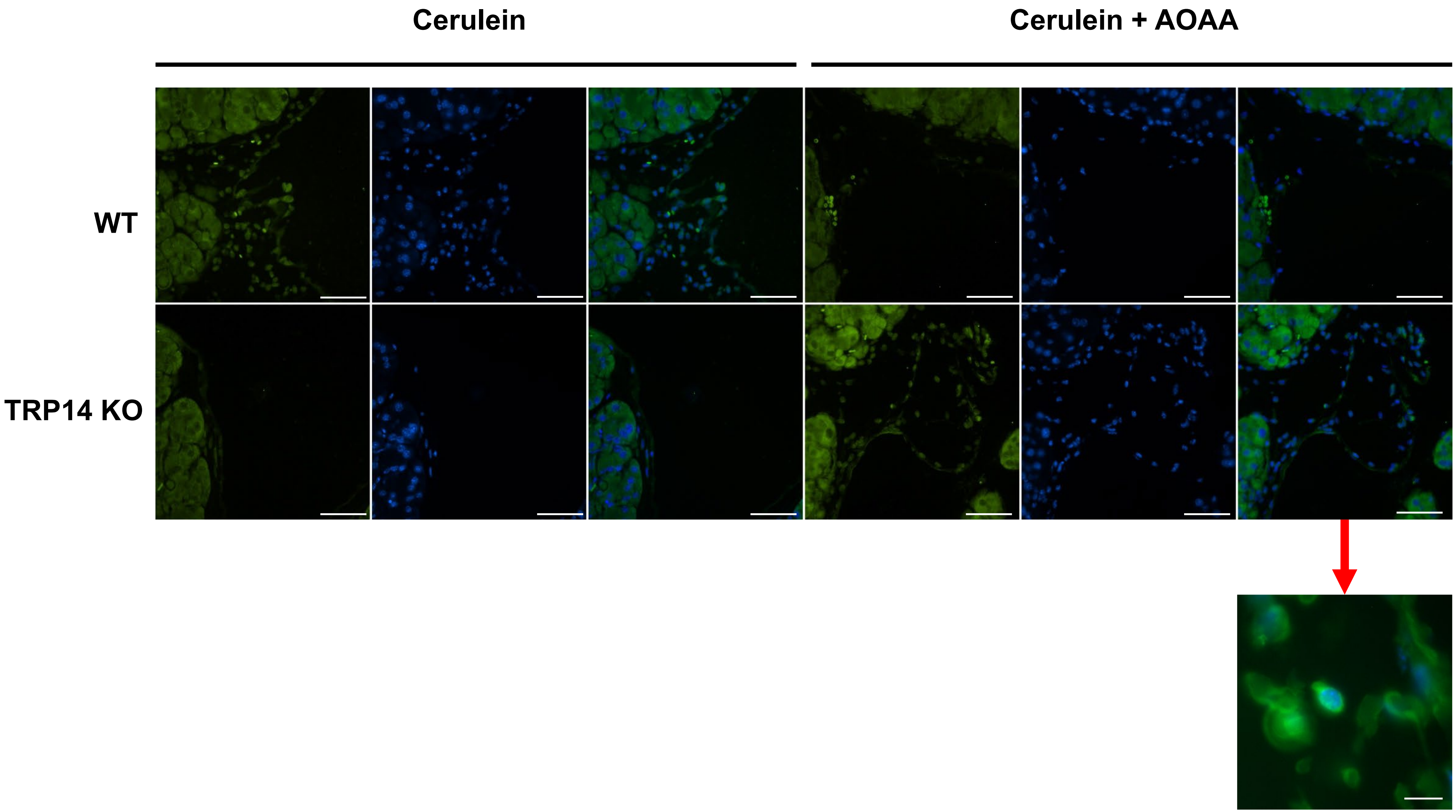

Appendix Figure S4. Representative immunohistochemistry images of mouse pancreatic tissue stained for CD11b/Integrin Alpha M (green) showing activated neutrophiles infiltrated in the pancreas of wild type and TRP14 KO mice with pancreatitis with and without AOAA administration. Hoechst staining (blue) shows nuclei. Scale bar: 50 μm (10 μm in the magnified image)

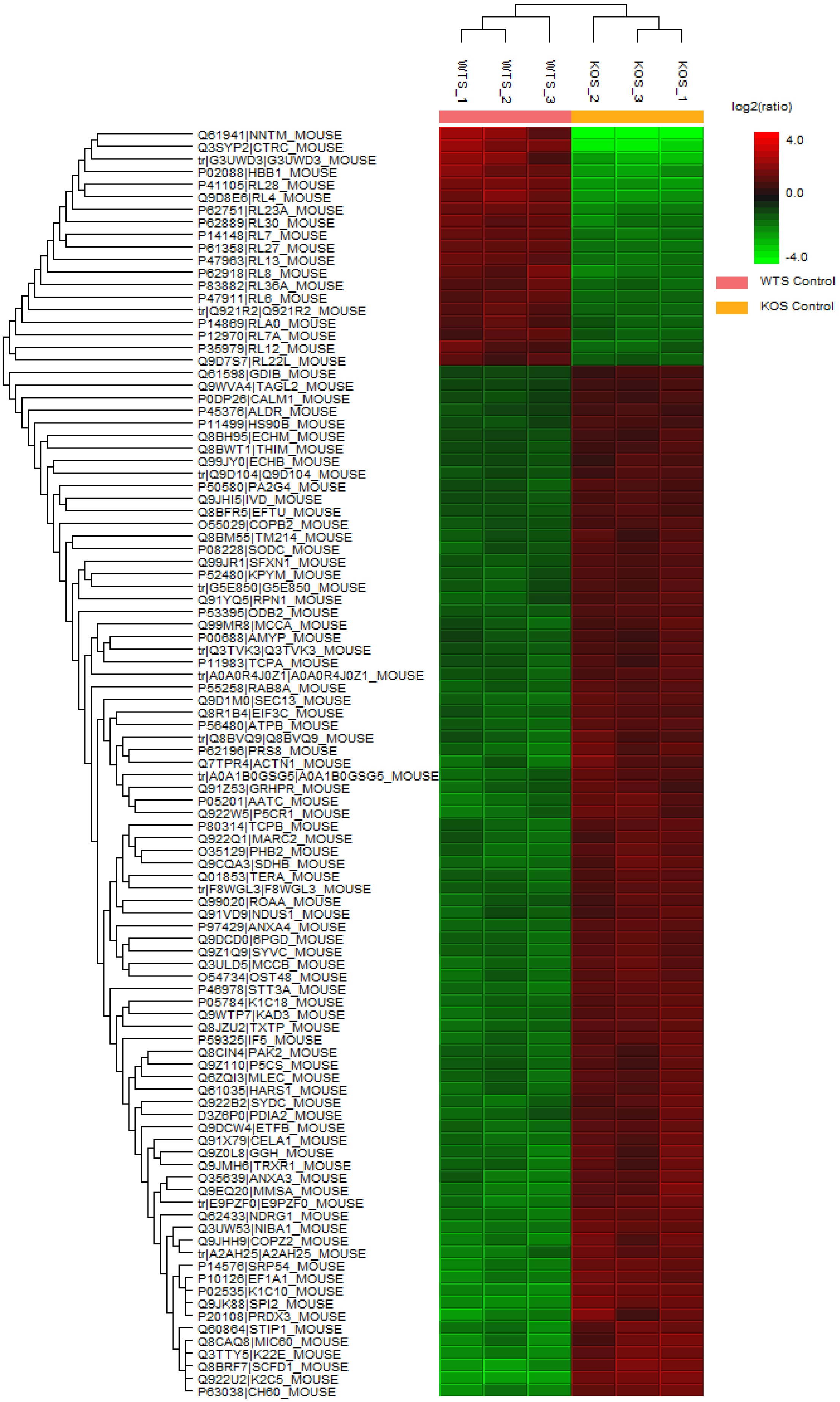

Appendix Figure S5. ***In vivo* proteomics.** Heat map showing protein levels in pancreas from sham wild type and TRP14 knockout mice.

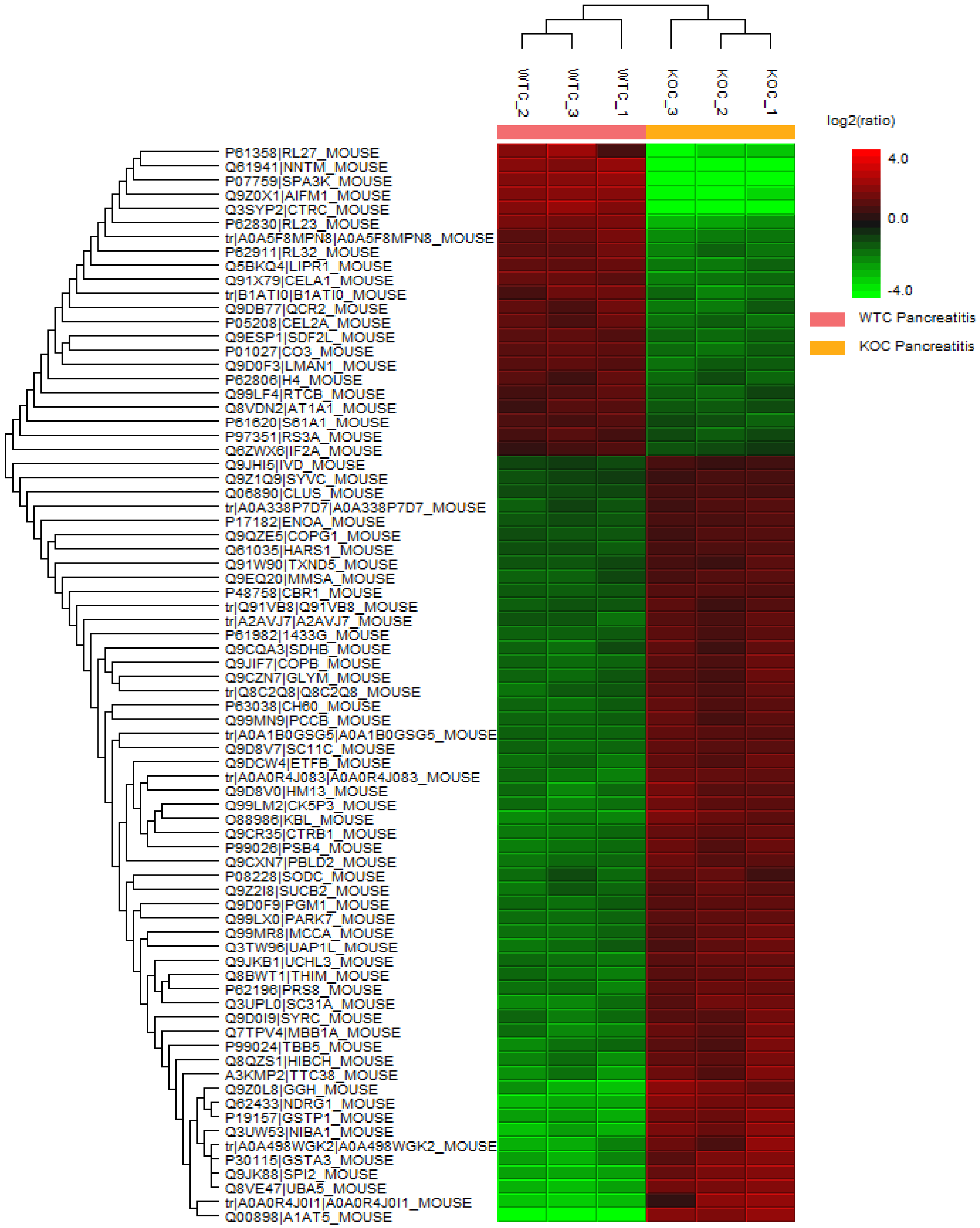

Appendix Figure S6. ***In vivo* proteomics.** Heat map showing protein levels in pancreas from TRP14 knockout mice in sham animals and after acute pancreatitis induction.

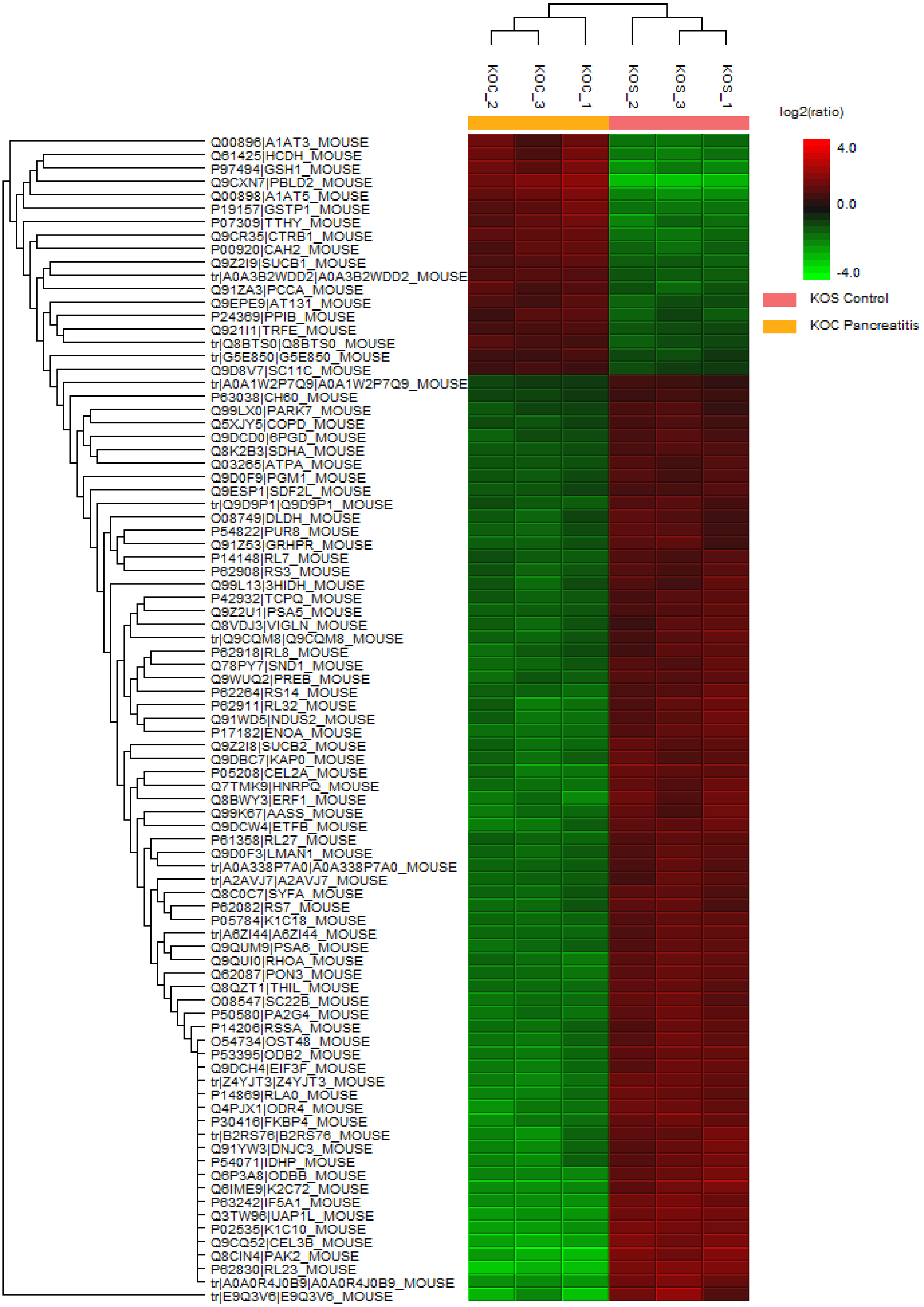

Appendix Figure S7. ***In vivo* proteomics.** Heat map showing protein levels in pancreas from wild type and TRP14 knockout mice with pancreatitis.

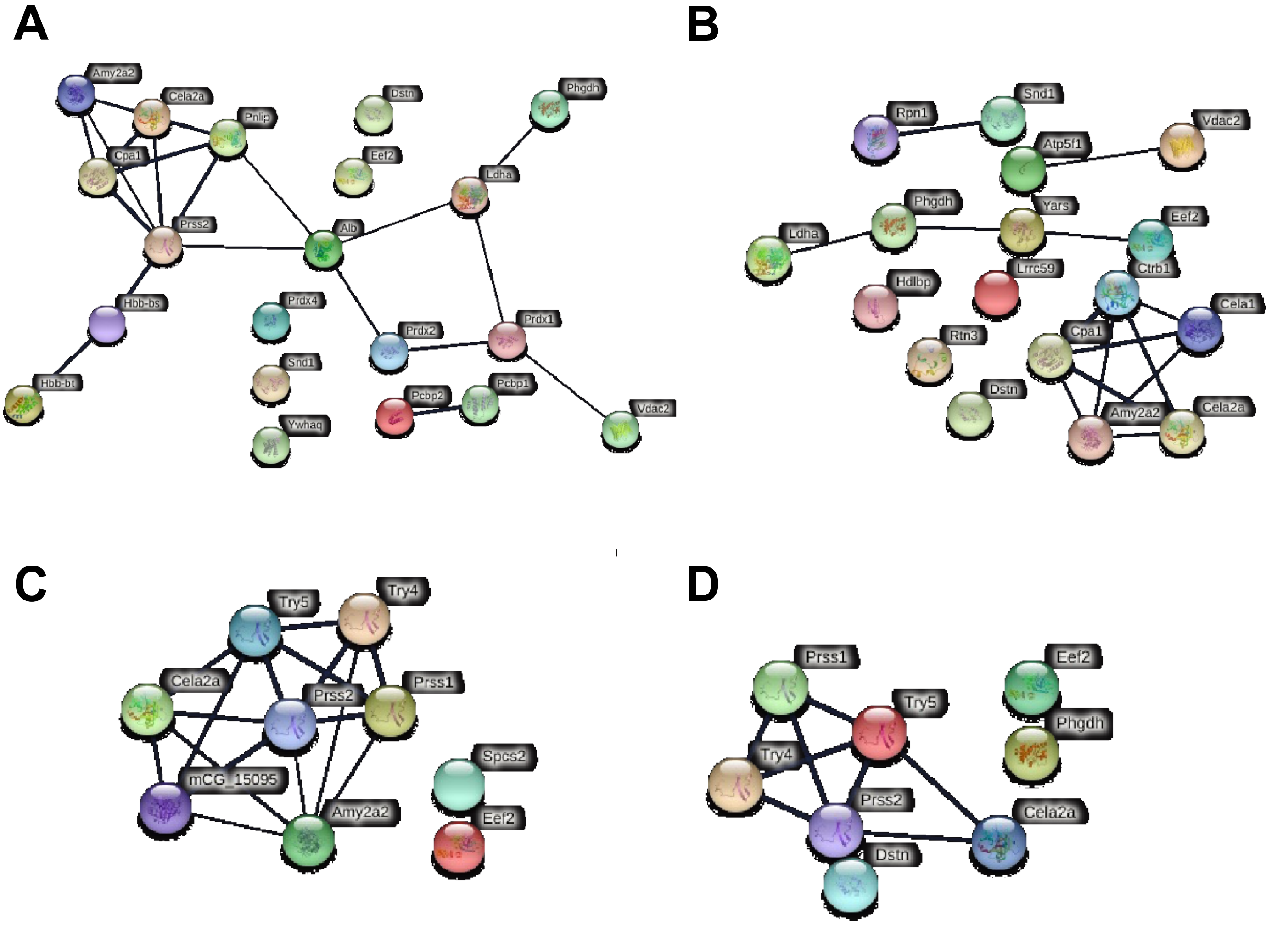

Appendix Figure S8. ***In vivo* proteomics. A)** String analysis showing protein-protein interactions among cysteinylated proteins identified in pancreas from wild type mice under basal conditions. **B)** String analysis showing protein-protein interactions among cysteinylated proteins identified in pancreas from TRP14 knockout mice under basal conditions. **C)** String analysis showing protein-protein interactions among cysteinylated proteins identified in pancreas from wild type mice with pancreatitis. **D)** String analysis showing protein-protein interactions among cysteinylated proteins identified in pancreas from TRP14 knockout mice upon acute pancreatitis induction.

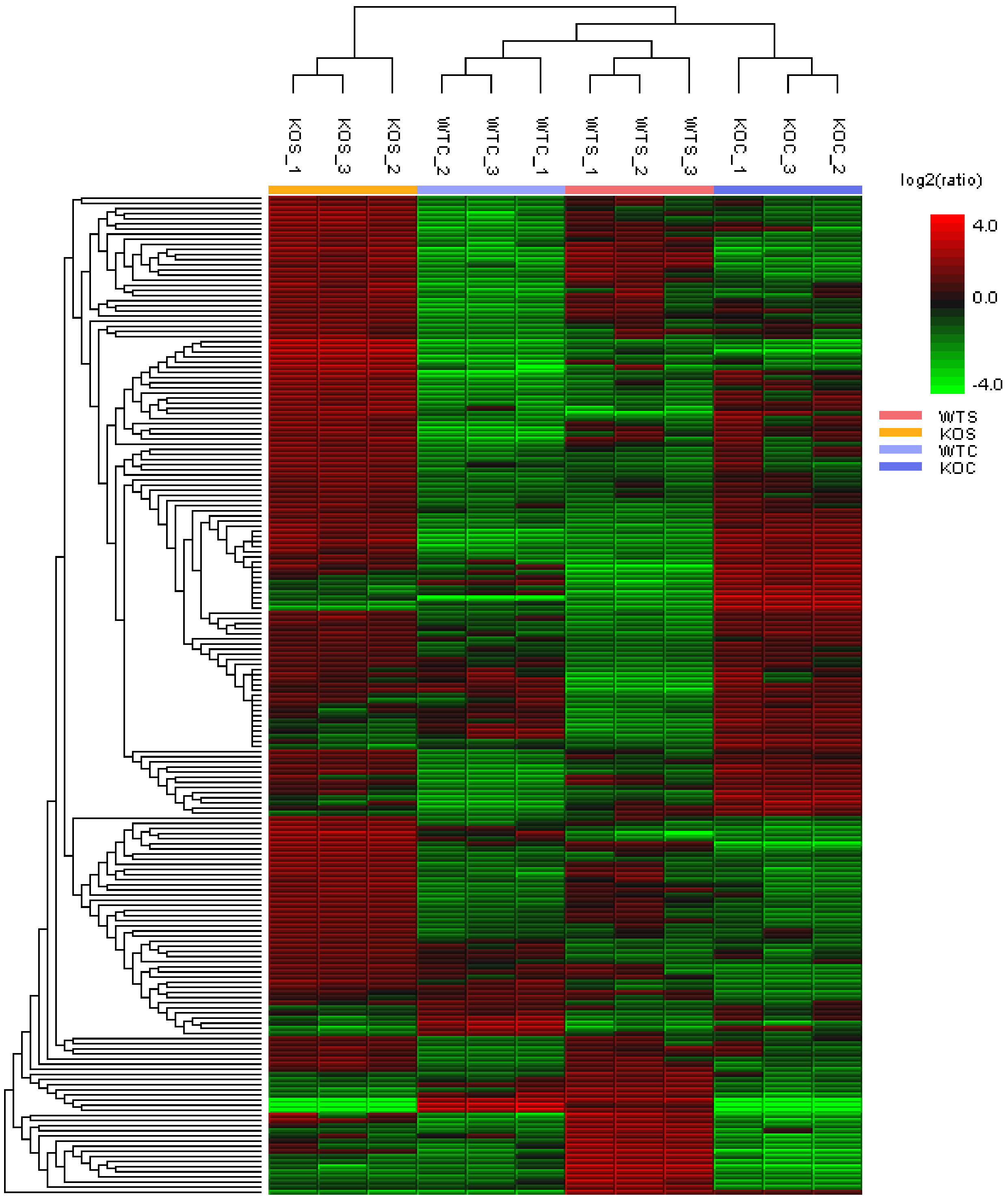

Appendix Figure S9. ***In vivo*** **proteomics: Differential protein expression**. Heat map showing protein levels in pancreas from sham wild type and TRP14 knockout mice and wild type and TRP14 knockout mice with pancreatitis.

APPENDIX TABLE S1

Cysteinylated proteins - WT animals with panceratitis

Protein code	Protein name
P05208	Chymotrypsin-like elastase family member 2A (EC 3.4.21.71) (Elastase-2) (Elastase-2A)
Q78PY7	Staphylococcal nuclease domain-containing protein 1 (EC 3.1.31.1) (100 kDa coactivator) (p100 co-activator)
P02088	Hemoglobin subunit beta-1 (Beta-1-globin) (Hemoglobin beta-1 chain) (Hemoglobin beta-major chain)
P58252	Elongation factor 2 (EF-2)
Q7TPZ8	Carboxypeptidase A1 (EC 3.4.17.1)
P07724	Albumin
Q61753	D-3-phosphoglycerate dehydrogenase (3-PGDH) (EC 1.1.1.95) (A10)
Q9R0P5	Destrin (Actin-depolymerizing factor) (ADF) (Sid 23)
Q60930	Voltage-dependent anion-selective channel protein 2 (VDAC-2) (mVDAC2) (Outer mitochondrial membrane protein porin 2) (Voltage-dependent anion-selective channel protein 6) (VDAC-6) (mVDAC6)
P68254	14-3-3 protein theta (14-3-3 protein tau)
P06151	L-lactate dehydrogenase A chain (LDH-A) (EC 1.1.1.27) (LDH muscle subunit) (LDH-M)
P60335	Poly(rC)-binding protein 1 (Alpha-CP1) (Heterogeneous nuclear ribonucleoprotein E1) (hnRNP E1)
Q61990	Poly(rC)-binding protein 2 (Alpha-CP2) (CTBP) (CBP) (Putative heterogeneous nuclear ribonucleoprotein X) (hnRNP X)
B2M1R7	Poly(rC)-binding protein 2
P00688	Pancreatic alpha-amylase (PA) (EC 3.2.1.1) (1,4-alpha-D-glucan glucanohydrolase)
Q6P8U6	Pancreatic triacylglycerol lipase (PL) (PTL) (Pancreatic lipase) (EC 3.1.1.3)
A8DUK4	Beta-globin (Globin a1) (Hemoglobin, beta adult s chain) (Hemoglobin, beta adult t chain)
P07146	Anionic trypsin-2 (EC 3.4.21.4) (Anionic trypsin II) (Pretrypsinogen II) (Serine protease 2)
O08807	Peroxiredoxin-4 (EC 1.11.1.24) (Antioxidant enzyme AOE372) (Peroxiredoxin IV) (Prx-IV) (Thioredoxin peroxidase AO372) (Thioredoxin-dependent peroxide reductase A0372) (Thioredoxin-dependent peroxiredoxin 4)
P35700	Peroxiredoxin-1 (EC 1.11.1.24) (Macrophage 23 kDa stress protein) (Osteoblast-specific factor 3) (OSF-3) (Thioredoxin peroxidase 2) (Thioredoxin-dependent peroxide reductase 2) (Thioredoxin-dependent peroxiredoxin 1)
Q61171	Peroxiredoxin-2 (EC 1.11.1.24) (Thiol-specific antioxidant protein) (TSA) (Thioredoxin peroxidase 1) (Thioredoxin-dependent peroxide reductase 1) (Thioredoxin-dependent peroxiredoxin 2)
A0A1B0GSX0	L-lactate dehydrogenase

APPENDIX TABLE S2

Cysteinylated proteins - TRP14 knockout animals with panceratitis

Protein code	Protein name
P00688	Pancreatic alpha-amylase (PA) (EC 3.2.1.1) (1,4-alpha-D-glucan glucanohydrolase)
Q8VDJ3	Vigilin (High density lipoprotein-binding protein) (HDL-binding protein)
P05208	Chymotrypsin-like elastase family member 2A (EC 3.4.21.71) (Elastase-2) (Elastase-2A)
Q78PY7	Staphylococcal nuclease domain-containing protein 1 (EC 3.1.31.1) (100 kDa coactivator) (p100 co-activator)
P58252	Elongation factor 2 (EF-2)
Q7TPZ8	Carboxypeptidase A1 (EC 3.4.17.1)
Q91YQ5	Dolichyl-diphosphooligosaccharide--protein glycosyltransferase subunit 1 (Dolichyl-diphosphooligosaccharide--protein glycosyltransferase 67 kDa subunit) (Ribophorin I) (RPN-I) (Ribophorin-1)
Q9CR35	Chymotrypsinogen B (EC 3.4.21.1) [Cleaved into: Chymotrypsin B chain A; Chymotrypsin B chain B; Chymotrypsin B chain C]
Q61753	D-3-phosphoglycerate dehydrogenase (3-PGDH) (EC 1.1.1.95) (A10)
A2A7S7	Tyrosine--tRNA ligase (EC 6.1.1.1) (Tyrosyl-tRNA synthetase)
Q9R0P5	Destrin (Actin-depolymerizing factor) (ADF) (Sid 23)
Q60930	Voltage-dependent anion-selective channel protein 2 (VDAC-2) (mVDAC2) (Outer mitochondrial membrane protein porin 2) (Voltage-dependent anion-selective channel protein 6) (VDAC-6) (mVDAC6)
P06151	L-lactate dehydrogenase A chain (LDH-A) (EC 1.1.1.27) (LDH muscle subunit) (LDH-M)
Q922Q8	Leucine-rich repeat-containing protein 59 [Cleaved into: Leucine-rich repeat-containing protein 59, N-terminally processed]
Q91X79	Chymotrypsin-like elastase family member 1 (EC 3.4.21.36) (Elastase-1)
Q9CQQ7	ATP synthase F(0) complex subunit B1, mitochondrial (ATP synthase peripheral stalk-membrane subunit b) (ATP synthase subunit b) (ATPase subunit b)
Q9ES97	Reticulon-3
A0A1B0GSX0	L-lactate dehydrogenase

APPENDIX TABLE S3

Cysteinylated proteins - WT control animals

Protein code	Protein name
**P00688**	Pancreatic alpha-amylase (PA) (EC 3.2.1.1) (1,4-alpha-D-glucan glucanohydrolase)
**P05208**	Chymotrypsin-like elastase family member 2A (EC 3.4.21.71) (Elastase-2) (Elastase-2A)
**P58252**	Elongation factor 2 (EF-2)
**Q9CYN2**	Signal peptidase complex subunit 2 (EC 3.4.-.-) (Microsomal signal peptidase 25 kDa subunit) (SPase 25 kDa subunit)
**Q9CPN9**	RIKEN cDNA 2210010C04 gene (Trypsinogen 7)
**P07146**	Anionic trypsin-2 (EC 3.4.21.4) (Anionic trypsin II) (Pretrypsinogen II) (Serine protease 2)
**Q9R0T7**	Pancreatic trypsin (Trypsin 4) (Trypsinogen 8)
**Q9QUK9**	TESP4 (Trypsin 5) (Trypsinogen 9)
**Q9Z1R9**	Protease, serine 1 (trypsin 1) (Protease, serine, 1 (Trypsin 1)) (Trypsinogen 16)
**A0A140LHG8**	Microsomal signal peptidase 25 kDa subunit
**A0A140LJ01**	Microsomal signal peptidase 25 kDa subunit

APPENDIX TABLE S4

Cysteinylated proteins - TRP14 knockout animals

Protein code	Protein name
P05208	Chymotrypsin-like elastase family member 2A (EC 3.4.21.71) (Elastase-2) (Elastase-2A)
P58252	Elongation factor 2 (EF-2)
Q61753	D-3-phosphoglycerate dehydrogenase (3-PGDH) (EC 1.1.1.95) (A10)
Q9R0P5	Destrin (Actin-depolymerizing factor) (ADF) (Sid 23)
P07146	Anionic trypsin-2 (EC 3.4.21.4) (Anionic trypsin II) (Pretrypsinogen II) (Serine protease 2)
Q9R0T7	Pancreatic trypsin (Trypsin 4) (Trypsinogen 8)
Q9QUK9	TESP4 (Trypsin 5) (Trypsinogen 9)
Q9Z1R9	Protease, serine 1 (trypsin 1) (Protease, serine, 1 (Trypsin 1)) (Trypsinogen 16)

Appendix Table S5: *C. elegans* strains used in this study

Strain name	Genotype	Strain origin/construction
N2	*Wild type, DR subclone of CB original (Tc1 pattern I)*	[a]CGC
PHX4767	*txdc-17(syb4767) III*	This study, CRISPR-Cas9 editing on N2
VZ1053	*txdc-17(syb4767) III*	This study, PHX4767 outcrosed 2x with N2
VC4718	*cbs-1(gk5787[loxP + Pmyo-2::GFP::unc-54 3' UTR + Prps-27::neoR::unc-54 3' UTR + loxP]) X*	[a]CGC
MRF06	*cbs-2(ok666) II*	Outcrossed 6x with N2. Milos Filipovic gift
MRF03	*cth-1(ok3319) V*	Outcrossed 7x with N2. Milos Filipovic gift
WLSD968	*cth-2(mg599) II*	[a]Warnhoff and Ruvkun (2019) Nat. Chem. Biol. 15: 480-488
VB2616	*gcs-1(ok436) / mIn1 [dpy-10(e128) mIs14(Pmyo-2::gfp)] II*	Outcrossed 8x with N2. Simon Tuck gift
MT20108	*dpy-17(e164) unc-32(e189) / qC1 [dpy-19(e1259) glp-1(q339) nIs281[Pmyo-2::rfp] III*	[a]CGC
VZ1077	*cbs-2(ok666) II; cbs-1(gk5787[loxP + Pmyo-2::GFP::unc-54 3' UTR + Prps-27::neoR::unc-54 3' UTR + loxP]) X*	This study, MRF06 x VC4718
VZ1090	*txdc-17(syb4767) III; cth-1(ok3319) V*	This study, VZ1053 x MRF03
VZ1091	*cth-2(mg599) II; txdc-17(syb4767) III*	This study, WLSD968 x VZ1053
VZ1068	*cth-2(mg599) II; cth-1(ok3319) V*	This study, WLSD968 x MRF03
VZ1084	*cth-2(mg599) II; txdc-17(syb4767) III; cth-1(ok3319) V*	This study, VZ1053 x VZ1068
AM141	*rmIs133 [Punc-54::Q40::yfp] X*	[a]Morley *et al.* (2002) Proc. Natl. Acad. Sci. USA 99: 10417-10422
VZ1058	*txdc-17(syb4767) III; rmIs133 [Punc-54::Q40::yfp] X*	This study, VZ1053 x AM141
VZ1057	*cth-1(ok3319) V; rmIs133 [Punc-54::Q40::yfp] X*	This study, MRF03 x AM141
VZ1069	*cth-2(mg599) II; rmIs133 [Punc-54::Q40::yfp] X*	This study, WLSD968 x AM141
VZ1089	*txdc-17(syb4767) III; cth-1(ok3319) V; rmIs133 [Punc-54::Q40::yfp] X*	This study, VZ1053 x VZ1057
VZ1088[b]	*cth-2(mg599) II; txdc-17(syb4767) III; rmIs133 [Punc-54::Q40::yfp] / + X*	This study, VZ1053 x VZ1069
VZ1107[b]	*cth-2(mg599) / mIn1 [dpy-10(e128) mIs14(Pmyo-2::gfp)] II; txdc-17(syb4767) III; rmIs133 [Punc-54::Q40::yfp] / + X*	This study, VZ1088 x VB2616
VZ1108[b]	*cth-2(mg599) II; txdc-17(syb4767) / qC1 [dpy-19(e1259) glp-1(q339) nIs281[Pmyo-2::rfp] III; rmIs133 [Punc-54::Q40::yfp] / + [illegible]*	This study, VZ1088 x MT20108
HE250	*unc-52(e669su250) II*	[a]Mackenzie *et al.* (1978) Cell 15: 751-762
VZ1116	*unc-52(e669su250) II; txdc-17(syb4767) III; cth-1(ok3319) V*	This study, VZ1090 x HE250
VZ1100	*cth-2(mg599) unc-52(e669su250)*	This study, WLSD968 x HE250
Not filed strain[c]	*cth-2(mg599) unc-52(e669su250) / mIn1 [dpy-10(e128) mIs14(Pmyo-2::gfp)] II; txdc-17(syb4767) / + III*	This study, VZ1100 x VB2616 x VZ1053
Not filed strain[d]	*cth-2(mg599) unc-52(e669su250) / + II; txdc-17(syb4767) / qC1 [dpy-19(e1259) glp-1(q339) nIs281[Pmyo-2::rfp] III*	This study, VZ1053 x MT20108 x VZ1100

[a] CGC: *Caenorhaditis* Genetics Center

[b] Segregants with the *rmIs133* transgene in homozygosis die during embryogenesis or L1 larval stage

[c] Unable to isolate segregants with *txdc-17(syb4767)* in homozygosis

[d] Unable to isolate segregants with *cth-2(mg599) unc-52(e669su250)* in homozygosis

Appendix Table S6: protein and synthetic DNA sequence (codon optimized for *E.coli*)

Gene	Protein Sequence	Synthetic DNA sequence
C. elegans **TXNRD1**	MGSSHHHHHHGTMSDSEVN<br>QEAKPEVKPEVKPETHINL<br>KVSDGSSEIFFKIKKTTPL<br>RRLMEAFAKRQGKEMDSLR<br>FLYDGIRIQADQTPEDLDM<br>EDNDIIEAHREQIGGMKSL<br>TELFGCFKRQPRQQEASSP<br>ANPHVSDTLSMGVAASGMP<br>PPKRPAPAESPTLPGETLV<br>DAPGIPLKEALKEAANSKI<br>VIFYNSSDEEKQLVEFETY<br>LNSLKEPADAEKPLEIPEI<br>KKLQVSRASQKVIQYLTLH<br>TSWPLMYIKGNAVGGLKEL<br>KALKQDYLKEWLRDHTYDL<br>IVIGGGSGGLAAAKEASRL<br>GKKVACLDFVKPSPQGTSW<br>GLGGTCVNVGCIPKKLMHQ<br>ASLLGHSIHDAKKYGWKLP<br>EGKVEHQWNHLRDSVQDHI<br>ASLNWGYRVQLREKTVTYI<br>NSYGEFTGPFEISATNKKK<br>KVEKLTADRFLISTGLRPK<br>YPEIPGVKEYTITSDDLFQ<br>LPYSPGKTLCVGASYVSLE<br>CAGFLHGFGFDVTVMVRSI<br>LLRGFDQDMAERIRKHMIA<br>YGMKFEAGVPTRIEQIDEK<br>TDEKAGKYRVFWPKKNEET<br>GEMQEVSEEYNTILMAIGR<br>EAVTDDVGLTTIGVERAKS<br>KKVLGRREQSTTIPWVYAI<br>GDVLEGTPELTPVAIQAGR<br>VLMRRIFDGANELTEYDQI<br>PTTVFTPLEYGCCGLSEED<br>AMMKYGKDNIIIYHNVFNP<br>LEYTISERMDKDHCYLKMI<br>CLRNEEEKVVGFHILTPNA<br>GEVTQGFGIALKLAAKKAD<br>FDRLIGIHPTVAENFTTLT<br>LEKKEGDEELQASGCUG-	ATGGGCAGCAGCCATCATCATCATCATCACGGTACCATGTCGGACTCAGAAGTCAATC<br>AAGAAGCTAAGCCAGAGGTCAAGCCAGAAGTCAAGCCTGAGACTCACATCAATTTAAA<br>GGTGTCCGATGGATCTTCAGAGATCTTCTTCAAGATCAAAAAGACCACTCCTTTAAGA<br>AGGCTGATGGAAGCGTTCGCTAAAAGACAGGGTAAGGAAATGGACTCCTTAAGATTCT<br>TGTACGACGGTATTAGAATTCAAGCTGATCAGACCCCTGAAGATTTGGACATGGAGGA<br>TAACGATATTATTGAGGCTCACAGAGAACAGATTGGTGGTATGAAGTCGTTAACGGAG<br>TTATTCGGGTGTTTCAAACGTCAACCTCGCCAACAGGAGGCAAGTAGCCCTGCTAATC<br>CTCATGTTTCAGATACACTGAGCATGGGAGTGGCCGCTTCGGGTATGCCTCCCCCAAA<br>ACGCCCTGCACCCGCGGAATCACCAACCTTGCCGGGAGAAACGCTGGTAGACGCCCCT<br>GGTATTCCGCTTAAGGAAGCTCTTAAAGAAGCTGCGAACTCTAAGATCGTGATTTTCT<br>ATAATAGTAGTGACGAAGAGAAACAGTTAGTGGAATTTGAAACCTACCTGAACTCACT<br>TAAGGAACCTGCGGATGCCGAGAAGCCCTTGGAGATTCCTGAAATTAAAAAATTGCAG<br>GTGAGCCGTGCTAGTCAGAAGGTAATCCAGTACTTGACGTTGCACACCAGCTGGCCTT<br>TAATGTACATCAAAGGTAATGCGGTAGGAGGCTTGAAGGAACTGAAGGCACTTAAACA<br>AGATTATTTGAAGGAGTGGCTGCGCGACCACACCTATGATCTTATTGTAATCGGAGGC<br>GGAAGCGGGGGGTTAGCTGCCGCGAAAGAAGCATCCCGCCTTGGCAAAAAGGTAGCGT<br>GTTTAGATTTTGTCAAACCTAGCCCACAAGGGACCAGCTGGGGGTTGGGGGGTACGTG<br>CGTAAACGTGGGGTGCATCCCCAAAAAACTTATGCACCAGGCTAGCCTTTTAGGGCAT<br>TCCATCCACGACGCGAAAAAATACGGCTGGAAATTACCCGAAGGAAAAGTTGAACATC<br>AATGGAACCATCTTCGTGATTCAGTGCAGGACCACATTGCTAGCTTAAACTGGGGTTA<br>TCGTGTGCAGCTTCGTGAAAAGACAGTGACGTATATCAATTCGTATGGAGAGTTCACC<br>GGTCCCTTCGAGATTAGCGCCACCAACAAGAAGAAAAAAGTAGAAAAGTTGACCGCGG<br>ATCGTTTCTTAATTTCTACAGGTCTGCGTCCCAAATACCCTGAGATCCCGGGAGTGAA<br>AGAGTACACAATTACCTCGGATGATTTGTTCCAGTTACCTTACAGTCCTGGAAAGACT<br>CTGTGCGTAGGTGCGAGTTATGTTTCCTTAGAATGTGCGGGATTCCTGCACGGATTCG<br>GCTTTGACGTAACTGTGATGGTTCGTTCTATCTTGTTACGTGGCTTCGACCAGGATAT<br>GGCAGAGCGTATTCGTAAGCACATGATCGCCTACGGGATGAAGTTCGAAGCAGGTGTT<br>CCGACTCGCATTGAGCAAATCGACGAAAAGACTGACGAAAAAGCTGGCAAATATCGTG<br>TGTTCTGGCCGAAGAAGAATGAAGAAACTGGCGAGATGCAGGAGGTGAGCGAAGAATA<br>TAACACAATTCTTATGGCGATTGGACGCGAGGCAGTGACCGACGATGTTGGGTTGACG<br>ACAATCGGGGTAGAGCGCGCTAAATCAAAAAAAGTACTTGGGCGCCGCGAACAATCAA<br>CTACTATTCCTTGGGTTTATGCTATCGGTGATGTTCTGGAGGGGACTCCTGAACTGAC<br>GCCAGTCGCTATCCAGGCTGGGCGTGTTCTTATGCGCCGTATCTTTGACGGAGCCAAT<br>GAATTGACCGAATACGATCAAATTCCTACAACGGTCTTTACGCCTTTGGAGTACGGCT<br>GCTGTGGATTAAGTGAGGAGGATGCTATGATGAAATACGGCAAAGACAATATTATTAT<br>TTACCATAATGTTTTCAATCCTTTGGAGTATACAATTAGCGAGCGCATGGACAAAGAC<br>CATTGTTATCTGAAGATGATCTGTTTGCGTAATGAGGAAGAGAAGGTCGTCGGTTTTC<br>ATATCTTGACGCCTAATGCAGGTGAAGTTACTCAGGGATTTGGGATTGCGTTAAAACT<br>TGCAGCCAAAAAGGCTGATTTTGACCGTTTAATCGGAATCCACCCCACAGTAGCAGAA<br>AATTTTACGACGCTGACACTGGAAAAGAAAGAGGGAGATGAGGAATTACAGGCCTCCG<br>GCTGTTAGGGCTAATAATCGGTTGCAGGTCTGCACC
C. elegans **TXNDC17**	MGSSHHHHHHGTMSDSEVN<br>QEAKPEVKPEVKPETHINL<br>KVSDGSSEIFFKIKKTTPL<br>RRLMEAFAKRQGKEMDSLR<br>FLYDGIRIQADQTPEDLDM<br>EDNDIIEAHREQIGGMTGL<br>KHYTAQGYEAFQETLKSIG<br>KGKRVVALFTGSKILTTGE<br>SWCPDCVVAEPVVEEVIKD<br>AAVAGLDVHFVTVFVGNRE<br>VWRDPAVGFRTDPTLKLTC<br>IPTLLEVGNKAKRLLERQI<br>ANKHLVKDFFTEED-	ATGGGCAGCAGCCATCATCATCATCATCACGGTACCATGTCGGACTCAGAAGTCAATC<br>AAGAAGCTAAGCCAGAGGTCAAGCCAGAAGTCAAGCCTGAGACTCACATCAATTTAAA<br>GGTGTCCGATGGATCTTCAGAGATCTTCTTCAAGATCAAAAAGACCACTCCTTTAAGA<br>AGGCTGATGGAAGCGTTCGCTAAAAGACAGGGTAAGGAAATGGACTCCTTAAGATTCT<br>TGTACGACGGTATTAGAATTCAAGCTGATCAGACCCCTGAAGATTTGGACATGGAGGA<br>TAACGATATTATTGAGGCTCACAGAGAACAGATTGGTGGTATGACGGGCCTTAAGCAT<br>TACACAGCTCAGGGATACGAGGCATTCCAAGAGACGCTGAAGTCCATCGGTAAGGGAA<br>AGCGCGTAGTAGCATTGTTTACGGGGAGCAAGATCCTGACGACAGGTGAAAGTTGGTG<br>CCCAGACTGTGTCGTAGCGGAACCCGTCGTGGAGGAAGTCATTAAAGATGCAGCGGTT<br>GCAGGTCTGGATGTCCACTTTGTTACCGTTTTCGTCGGTAACCGCGAGGTATGGCGCG<br>ACCCTGCGGTTGGGTTCCGCACAGATCCGACGCTTAAGCTGACGTGCATCCCTACATT<br>ACTGGAGGTAGGTAACAAGGCCAAGCGTTTGCTGGAACGTCAGATTGCTAATAAGCAT<br>TTAGTCAAGGACTTTTTCACTGAGGAGGATTAA

His-tag: underlined letter
SUMO sequence: green letter
Selenocysteine residue (U) and its codon (TAG): highlighted in yellow
Stop codon (TAA): highlighted in turquoise
SECIS element (only for TXNRD1 construct): highlighted in bright green
