# Supplementary figures and images for: TRP14 is the rate-limiting enzyme for intracellular cystine reduction and regulates proteome cysteinylation
Source: EMBO J. 2024 May 29;43(13):12. doi: 10.1038/s44318-024-00117-1 (PMC11217419; doi:10.1038/s44318-024-00117-1)

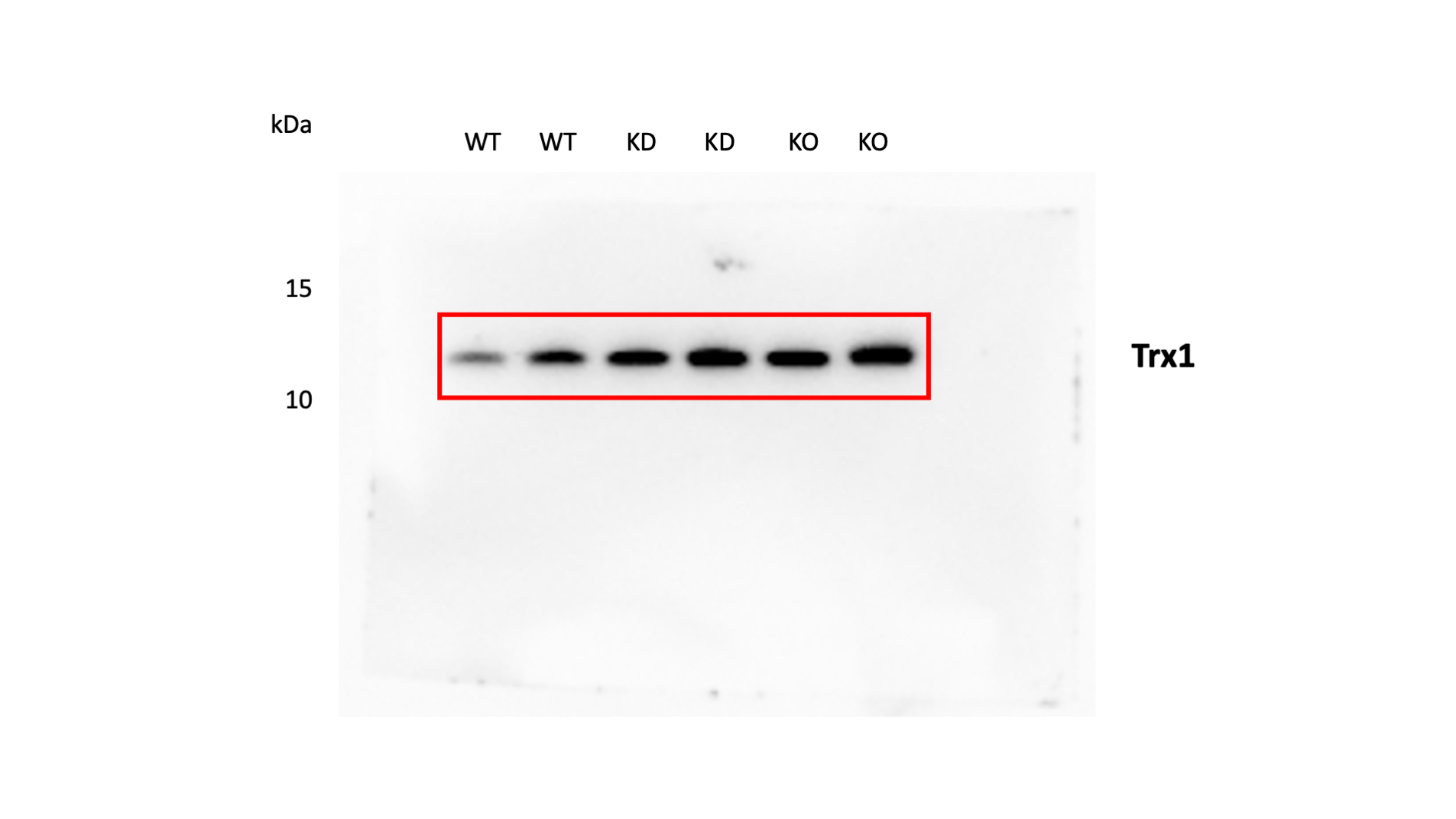

Supplement: Supplementary file 2 — Source data Fig. 1 [file 44318_2024_117_MOESM2_ESM.zip › Figure 1/1A/Western blot Trx1.tiff]

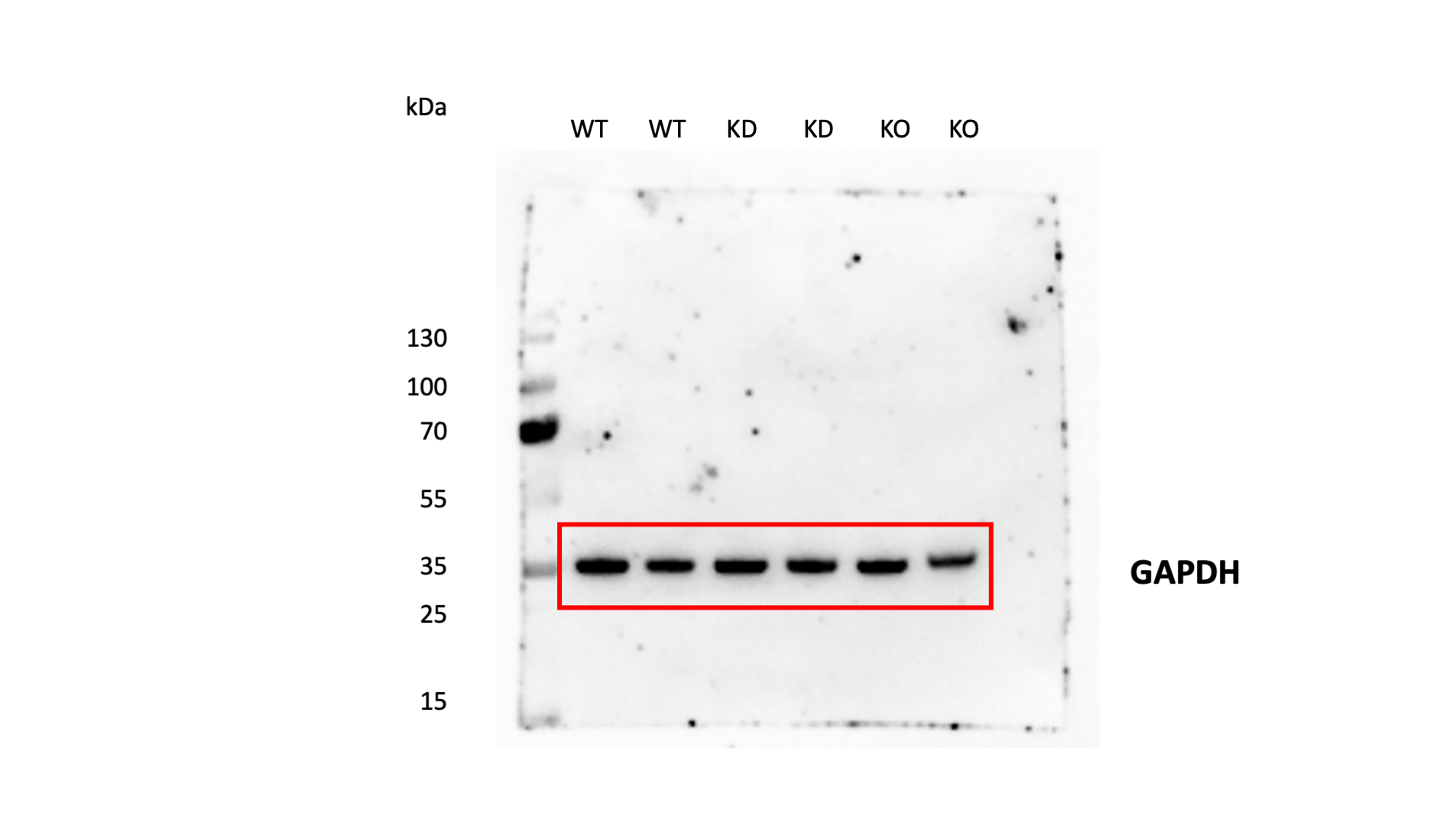

Supplement: Supplementary file 2 — Source data Fig. 1 [file 44318_2024_117_MOESM2_ESM.zip › Figure 1/1A/Western blot GAPDH.tiff]

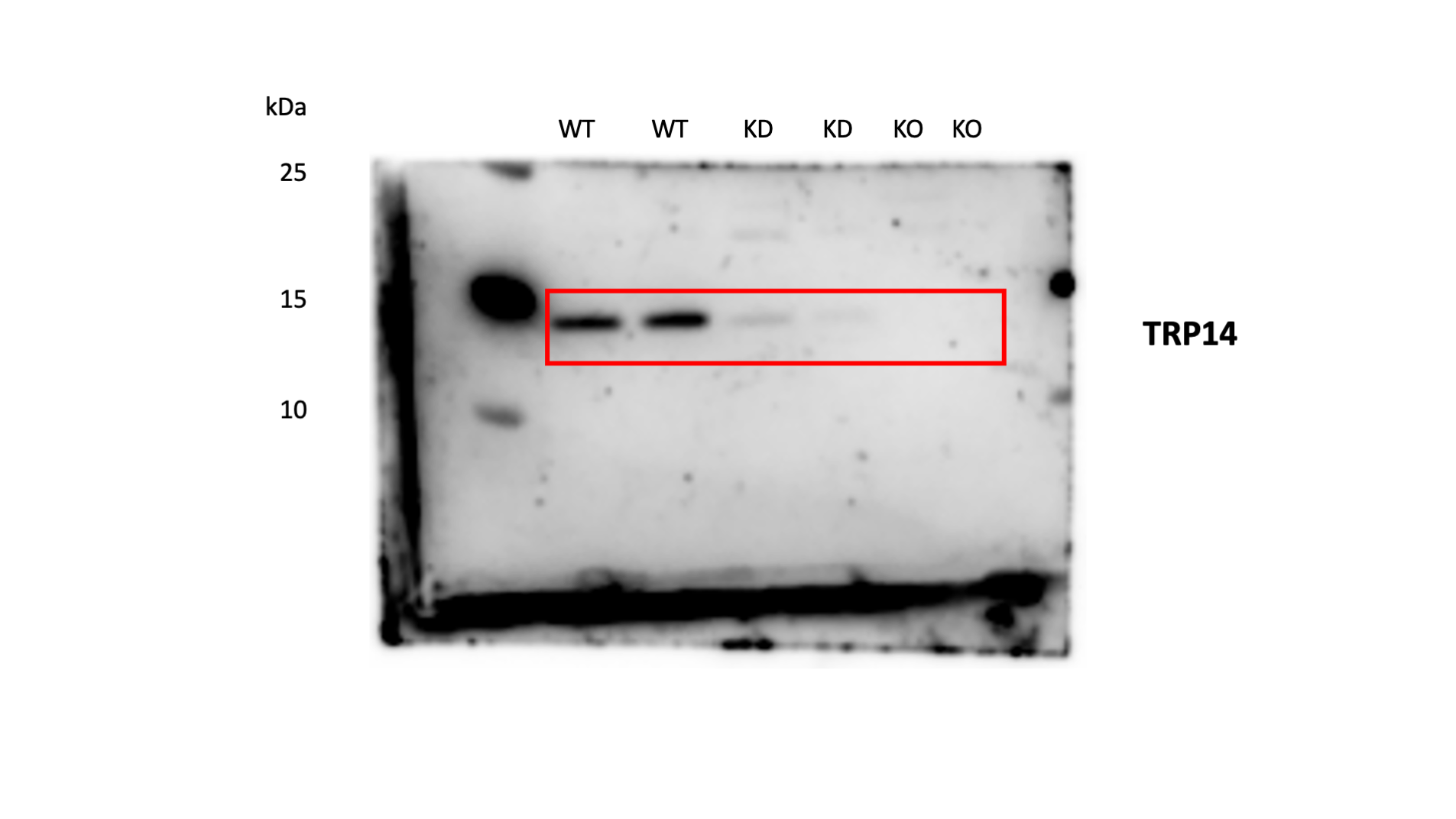

Supplement: Supplementary file 2 — Source data Fig. 1 [file 44318_2024_117_MOESM2_ESM.zip › Figure 1/1A/Western blot TRP14.tiff]

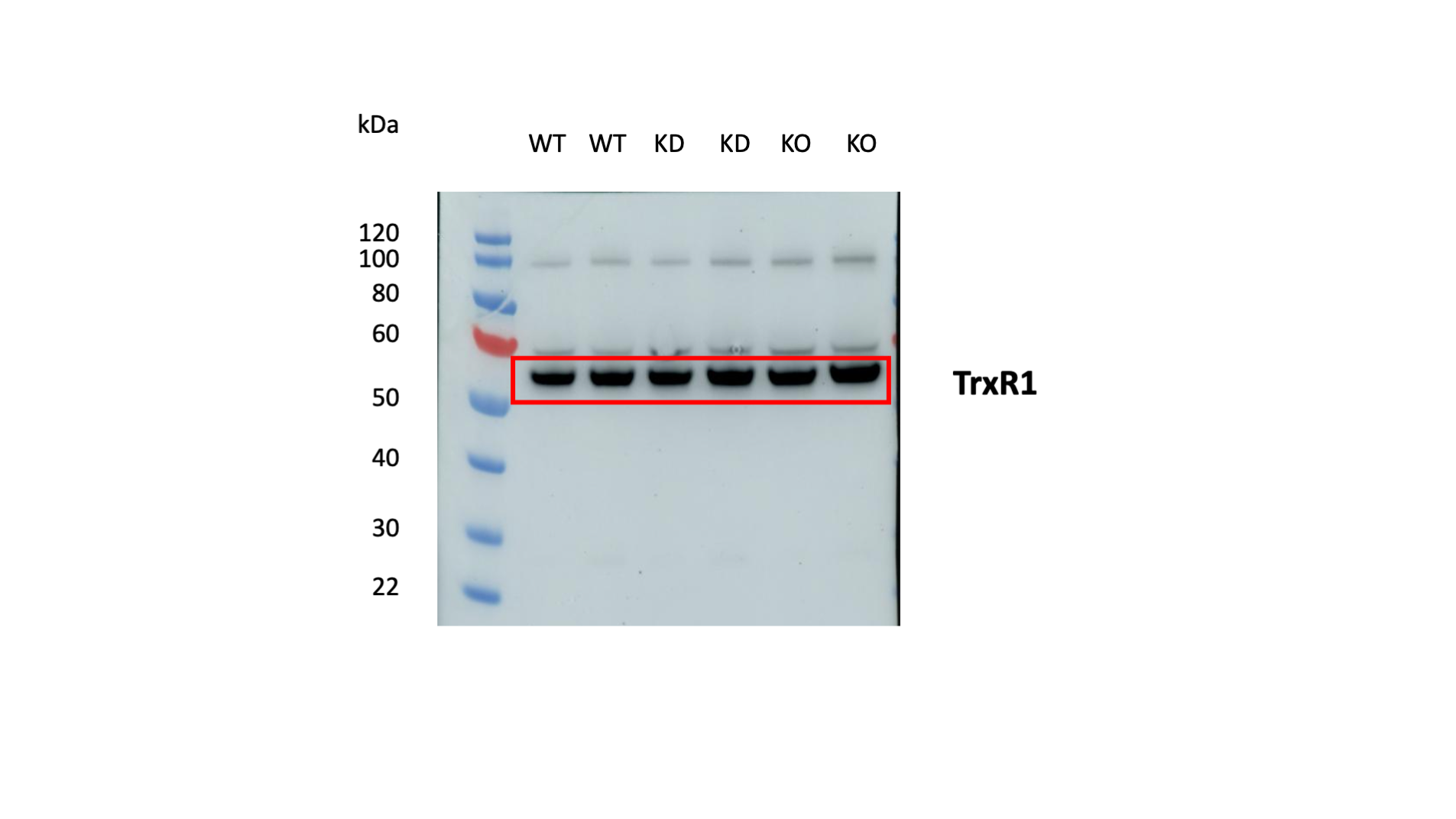

Supplement: Supplementary file 2 — Source data Fig. 1 [file 44318_2024_117_MOESM2_ESM.zip › Figure 1/1A/Western blot TrxR1.tiff]

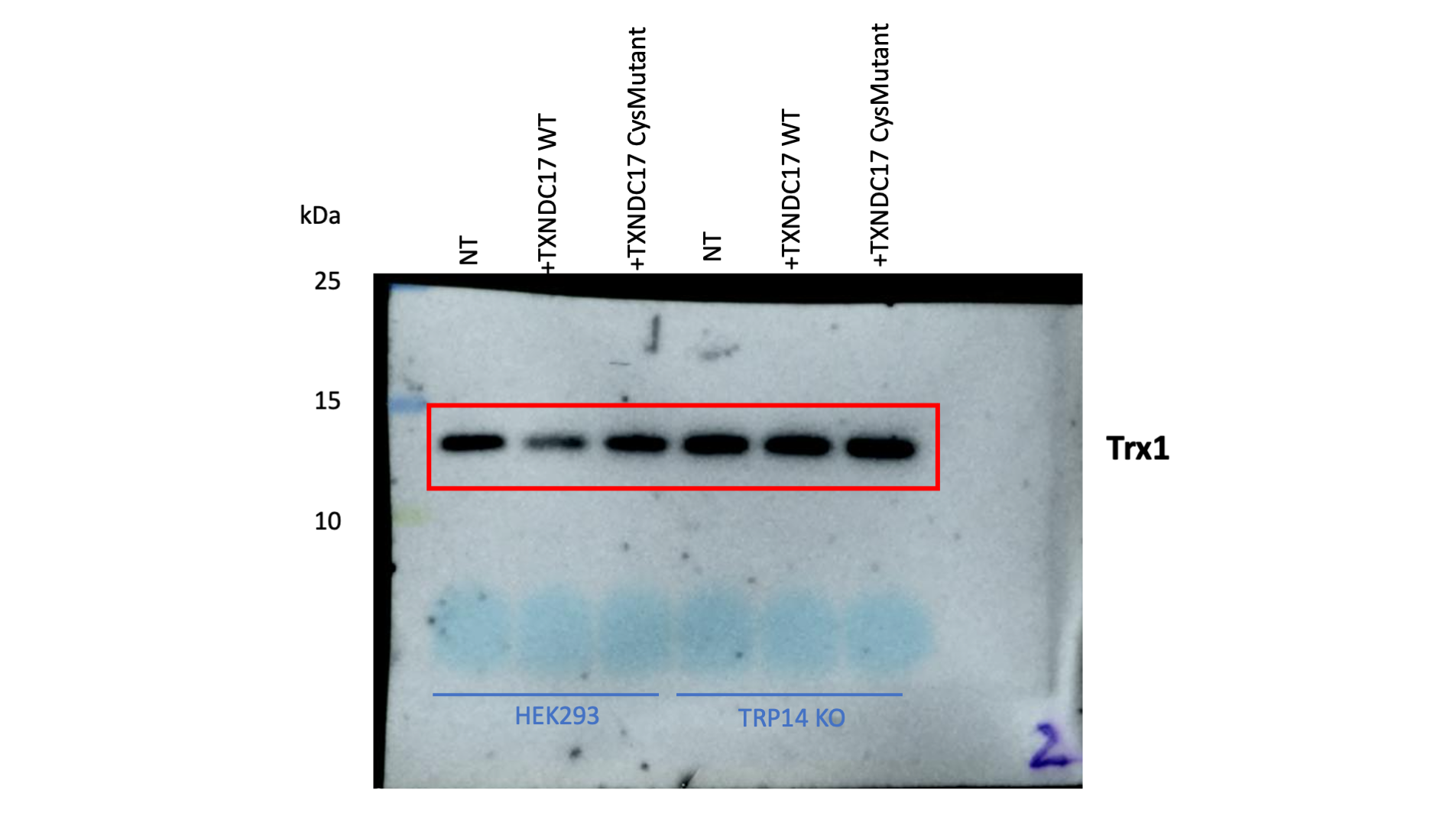

Supplement: Supplementary file 2 — Source data Fig. 1 [file 44318_2024_117_MOESM2_ESM.zip › Figure 1/1D/Western blot Trx1.tiff]

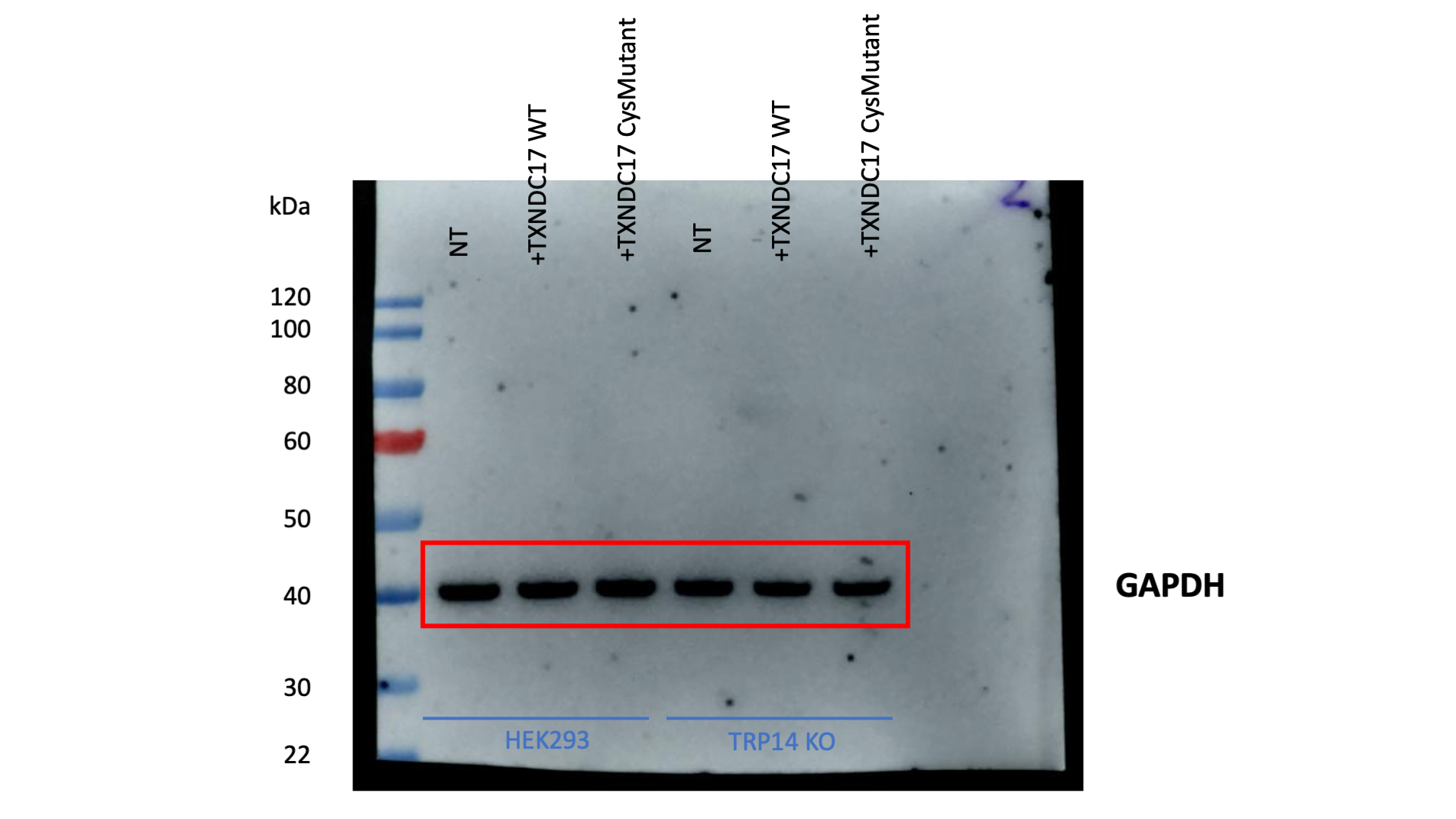

Supplement: Supplementary file 2 — Source data Fig. 1 [file 44318_2024_117_MOESM2_ESM.zip › Figure 1/1D/Western blot GAPDH.tiff]

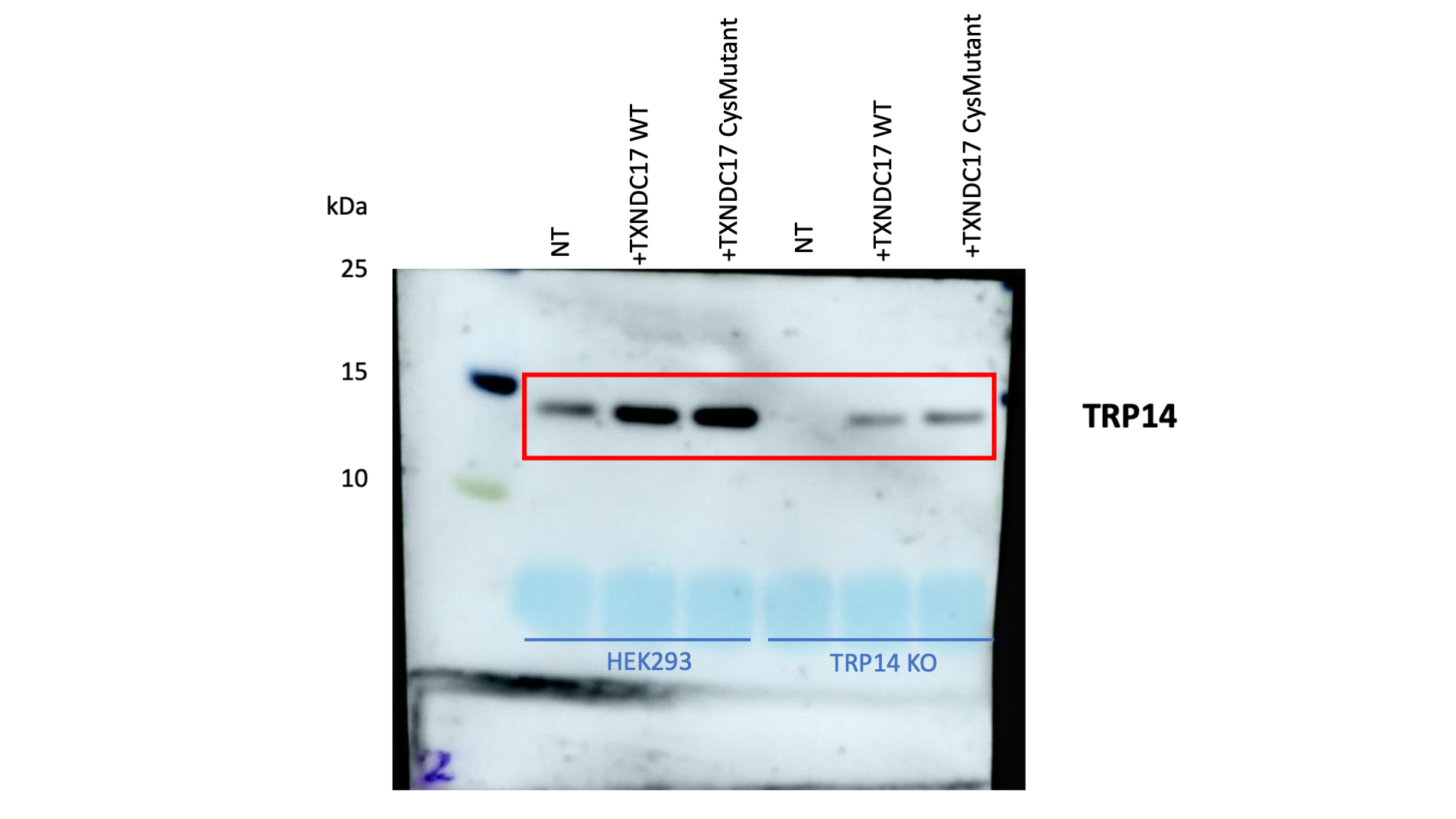

Supplement: Supplementary file 2 — Source data Fig. 1 [file 44318_2024_117_MOESM2_ESM.zip › Figure 1/1D/Western blot TRP14.tiff]

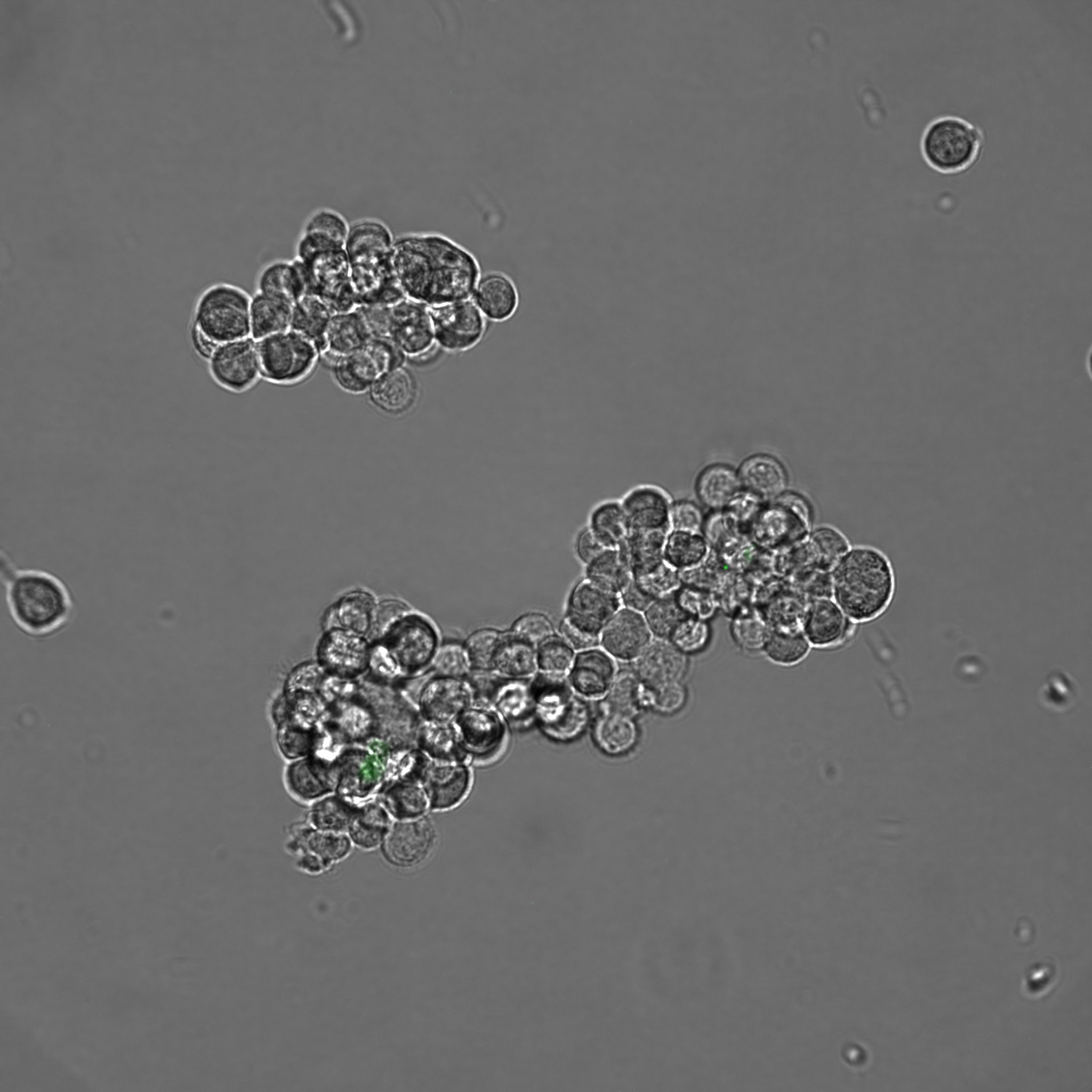

Supplement: Supplementary file 2 — Source data Fig. 1 [file 44318_2024_117_MOESM2_ESM.zip › Figure 1/1B/1B Microscopy images/40 X TRP14 KO 006 - MERGE.jpg]

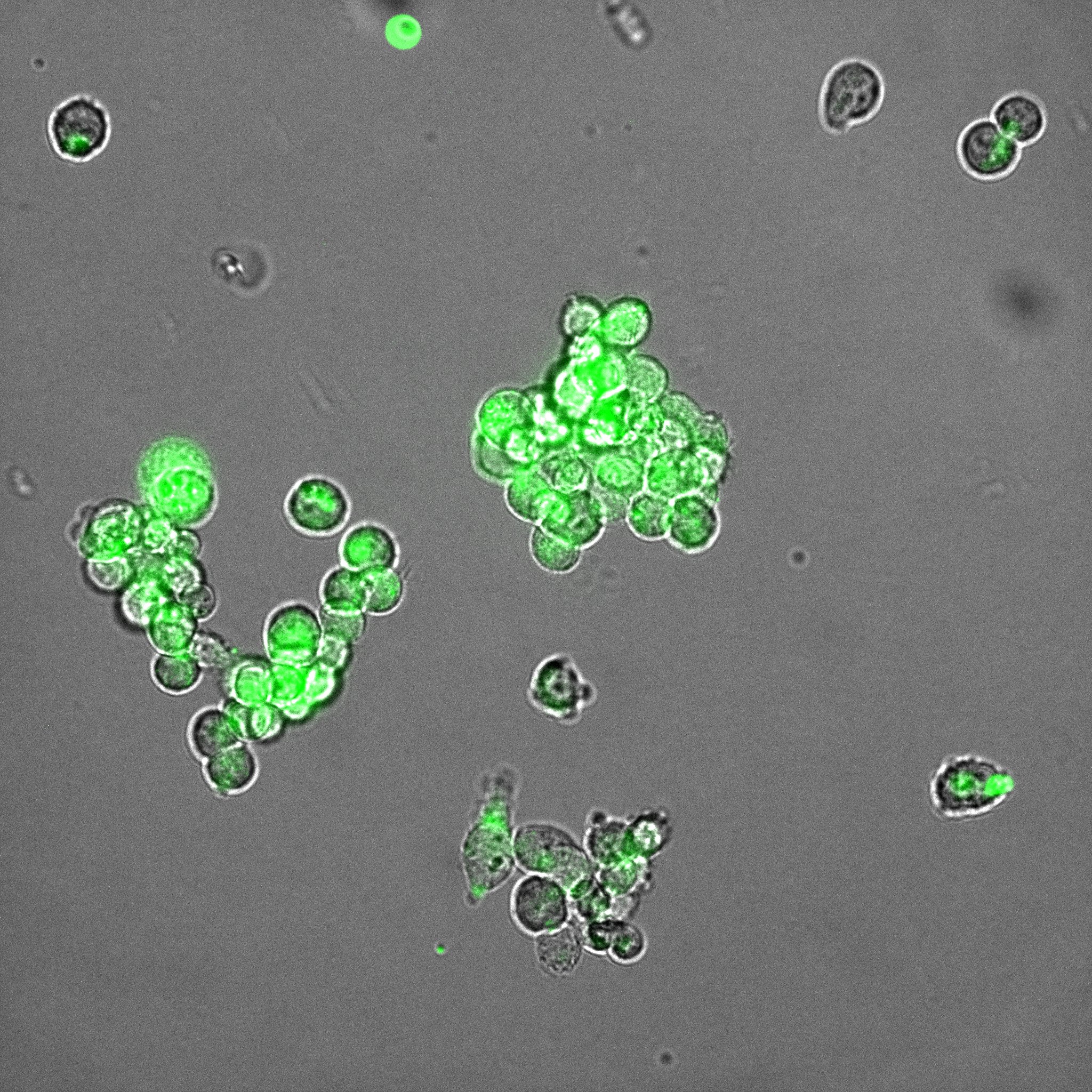

Supplement: Supplementary file 2 — Source data Fig. 1 [file 44318_2024_117_MOESM2_ESM.zip › Figure 1/1B/1B Microscopy images/40 X CT 005 - MERGE.jpg]

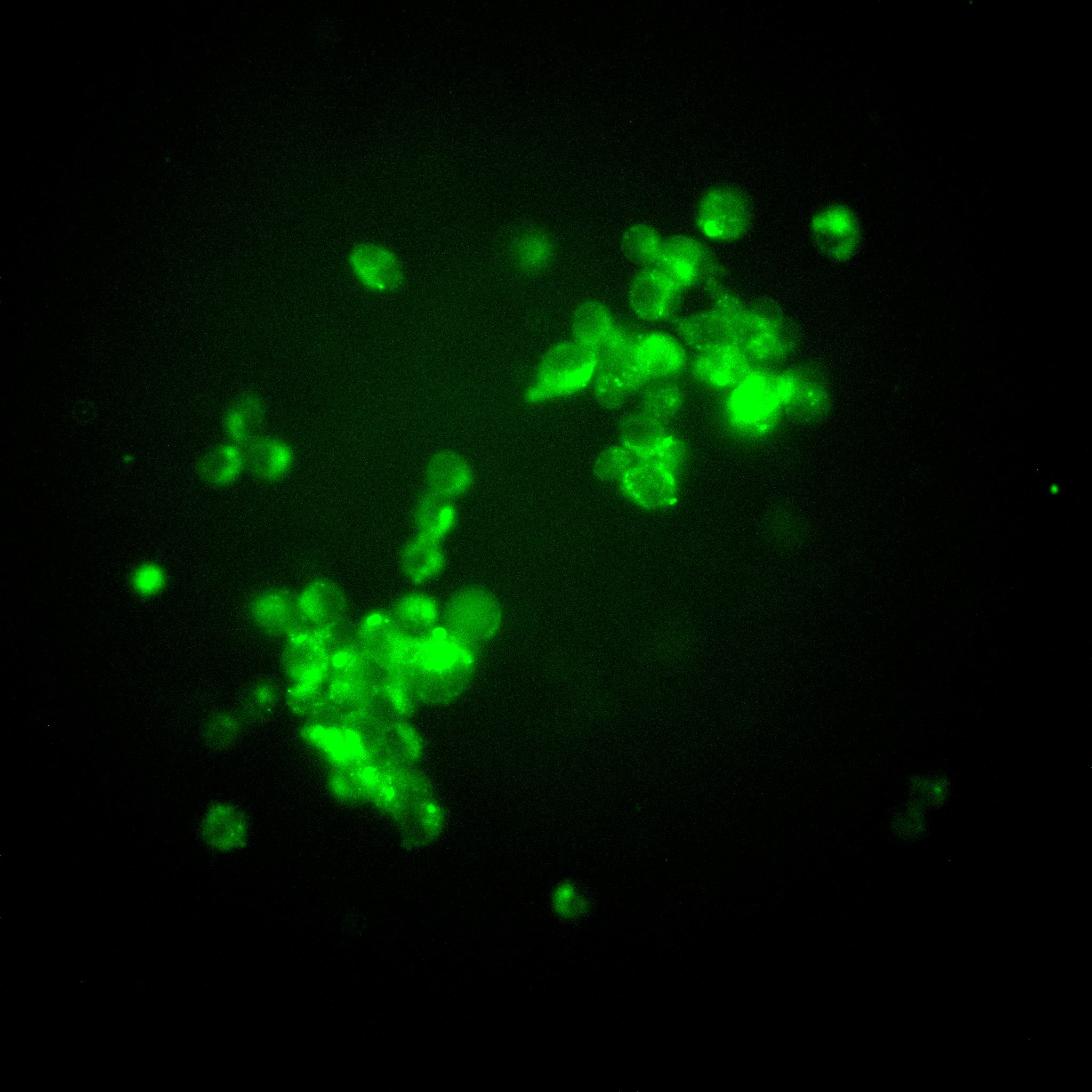

Supplement: Supplementary file 2 — Source data Fig. 1 [file 44318_2024_117_MOESM2_ESM.zip › Figure 1/1B/1B Microscopy images/40 X CT 004 - CySS.jpg]

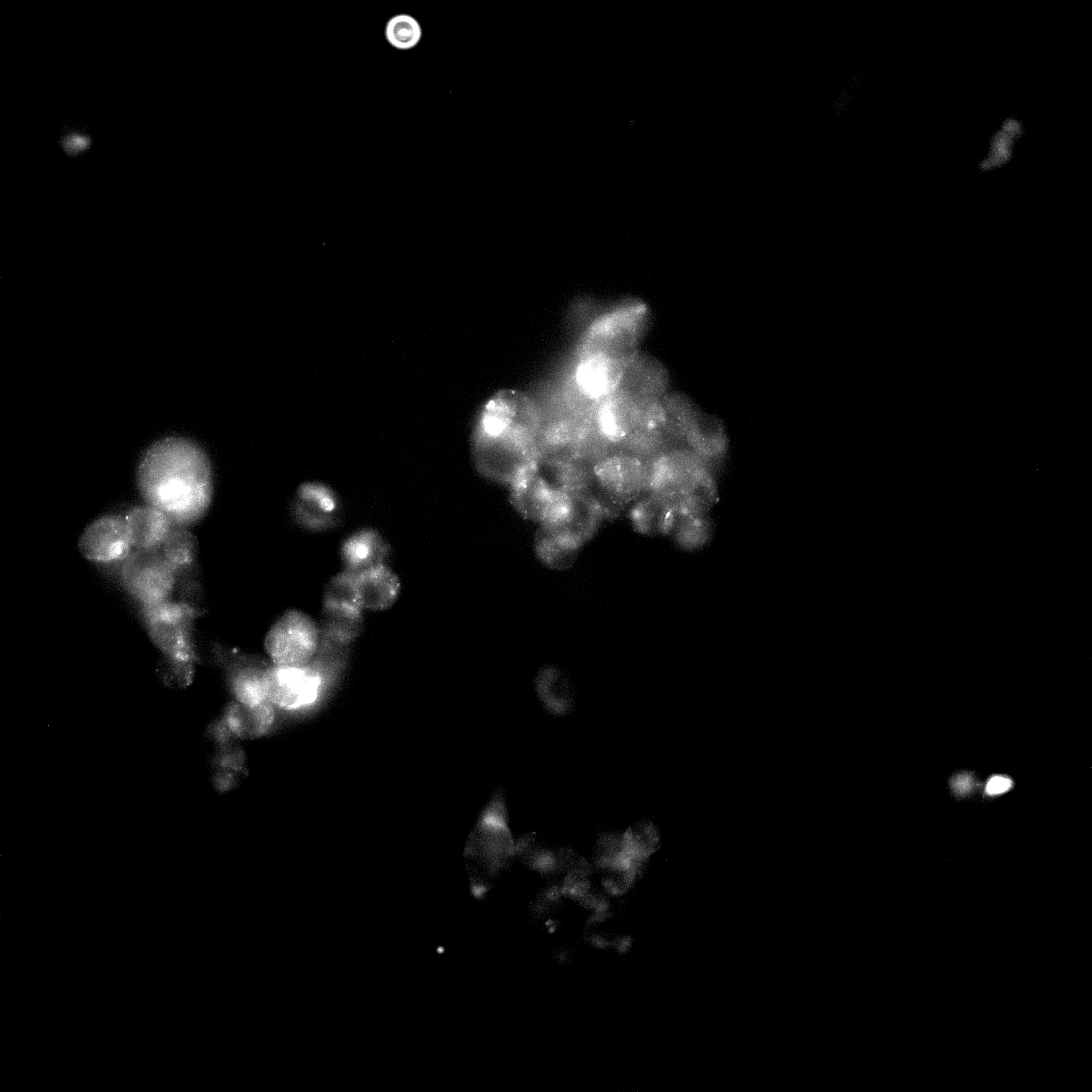

Supplement: Supplementary file 2 — Source data Fig. 1 [file 44318_2024_117_MOESM2_ESM.zip › Figure 1/1B/1B Microscopy images/40 X CT 005 - MERGE.tif]

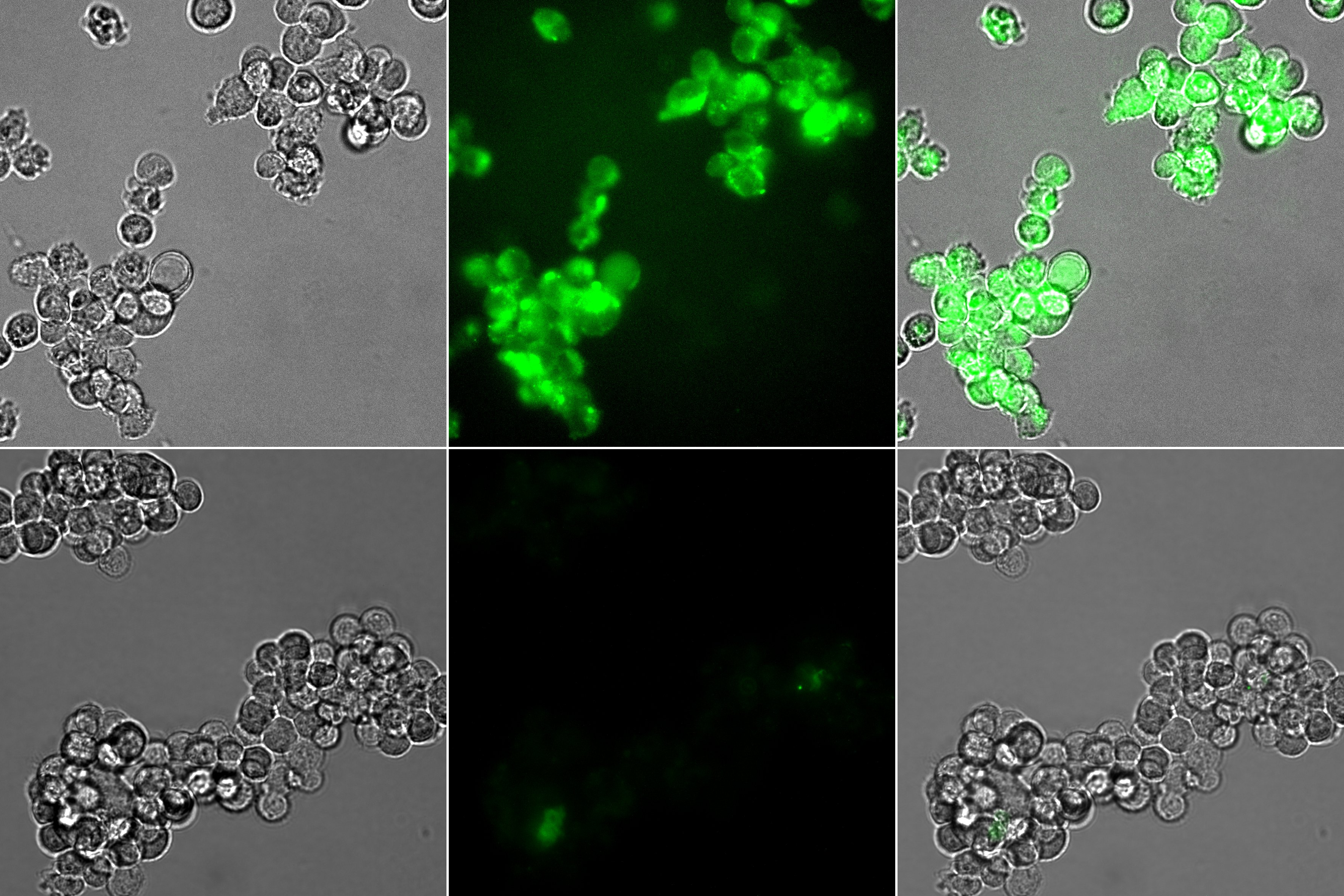

Supplement: Supplementary file 2 — Source data Fig. 1 [file 44318_2024_117_MOESM2_ESM.zip › Figure 1/1B/1B Microscopy images/Figure 1B CT TRP14 KO BODIPY CySS.tif]

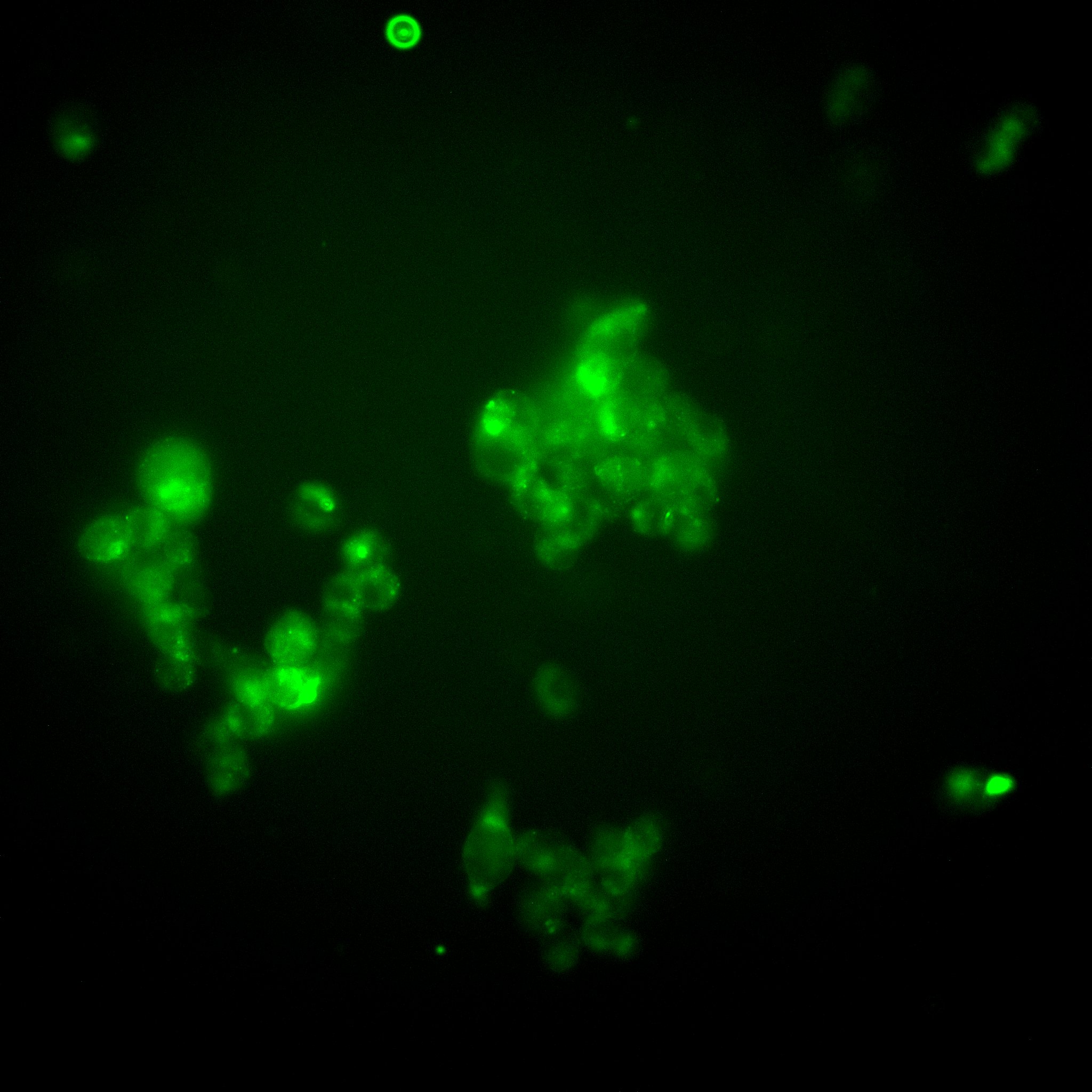

Supplement: Supplementary file 2 — Source data Fig. 1 [file 44318_2024_117_MOESM2_ESM.zip › Figure 1/1B/1B Microscopy images/40 X CT 005 - CySS.jpg]

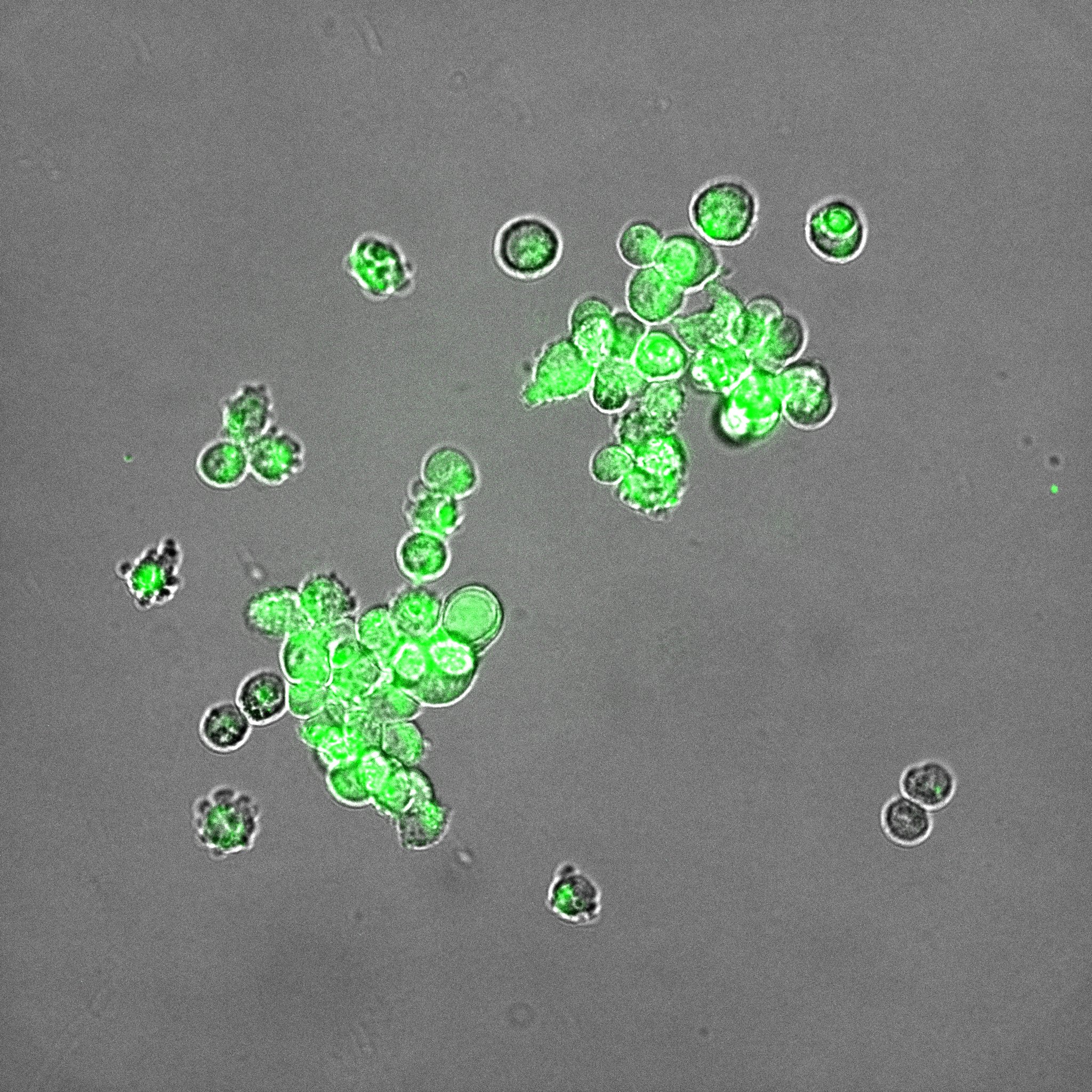

Supplement: Supplementary file 2 — Source data Fig. 1 [file 44318_2024_117_MOESM2_ESM.zip › Figure 1/1B/1B Microscopy images/40 X CT 004 - MERGE.jpg]

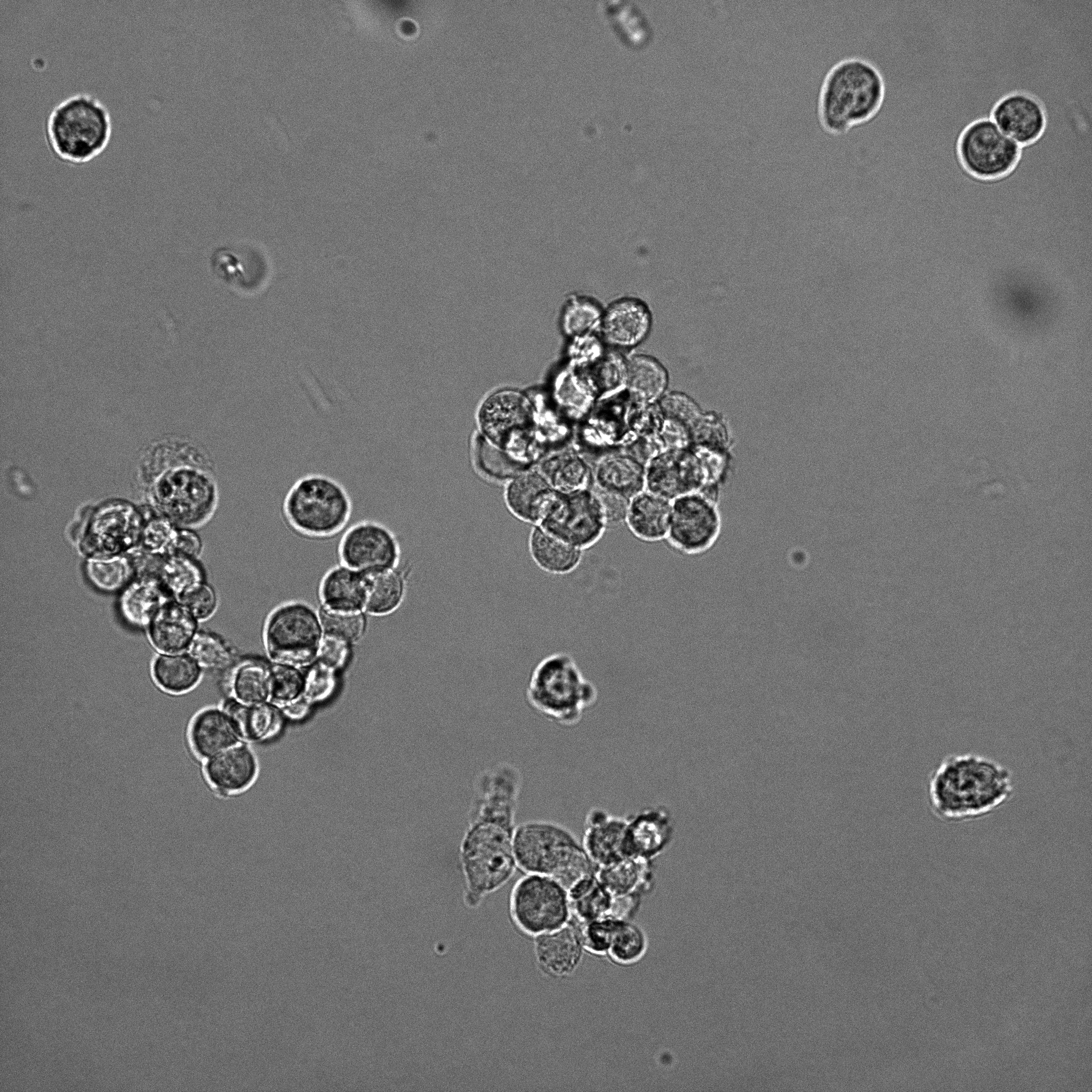

Supplement: Supplementary file 2 — Source data Fig. 1 [file 44318_2024_117_MOESM2_ESM.zip › Figure 1/1B/1B Microscopy images/40 X CT 005 - BF.tif]

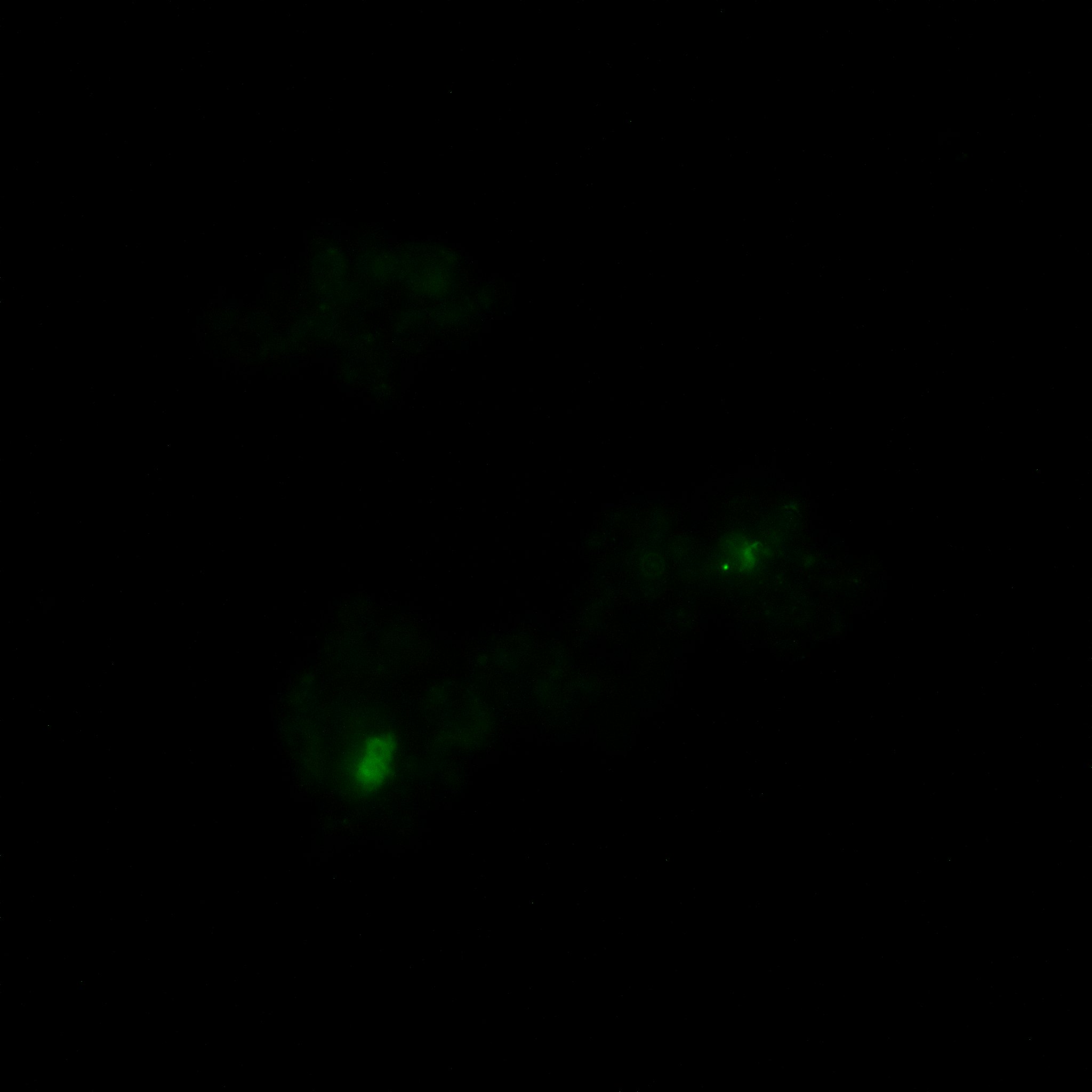

Supplement: Supplementary file 2 — Source data Fig. 1 [file 44318_2024_117_MOESM2_ESM.zip › Figure 1/1B/1B Microscopy images/40 X TRP14 KO 006 - CySS.jpg]

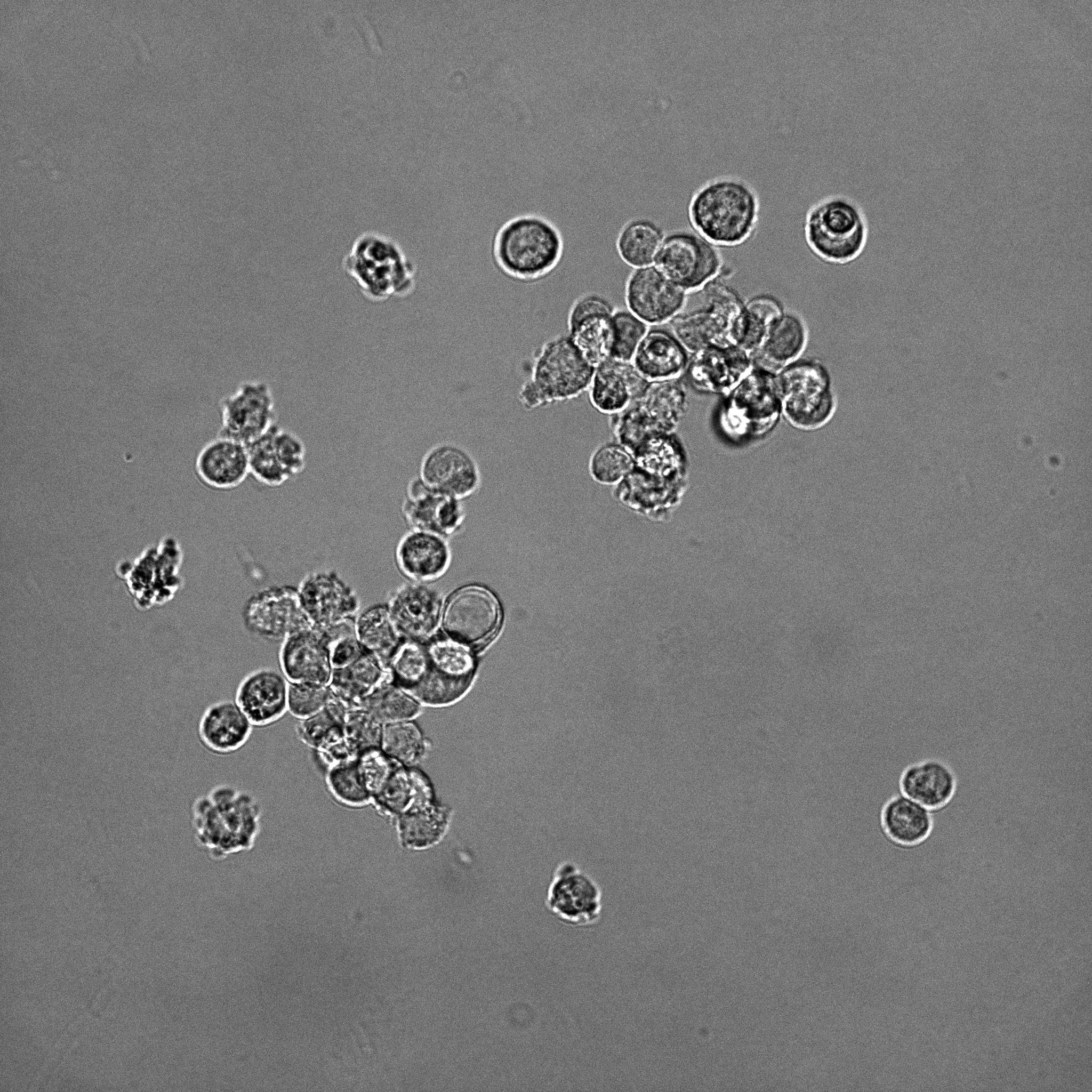

Supplement: Supplementary file 2 — Source data Fig. 1 [file 44318_2024_117_MOESM2_ESM.zip › Figure 1/1B/1B Microscopy images/40 X CT 004 - BF.jpg]

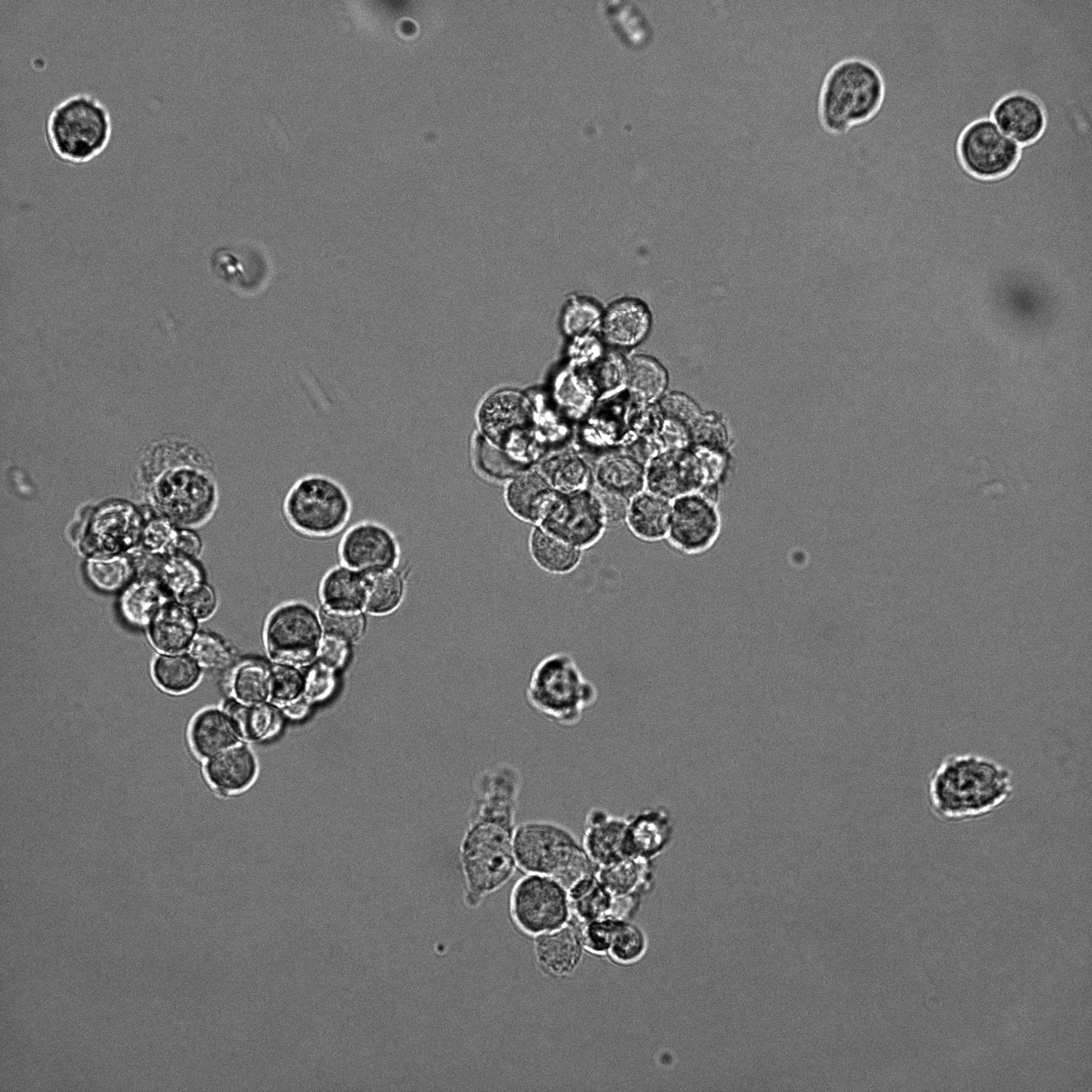

Supplement: Supplementary file 2 — Source data Fig. 1 [file 44318_2024_117_MOESM2_ESM.zip › Figure 1/1B/1B Microscopy images/40 X CT 005 - BF.jpg]

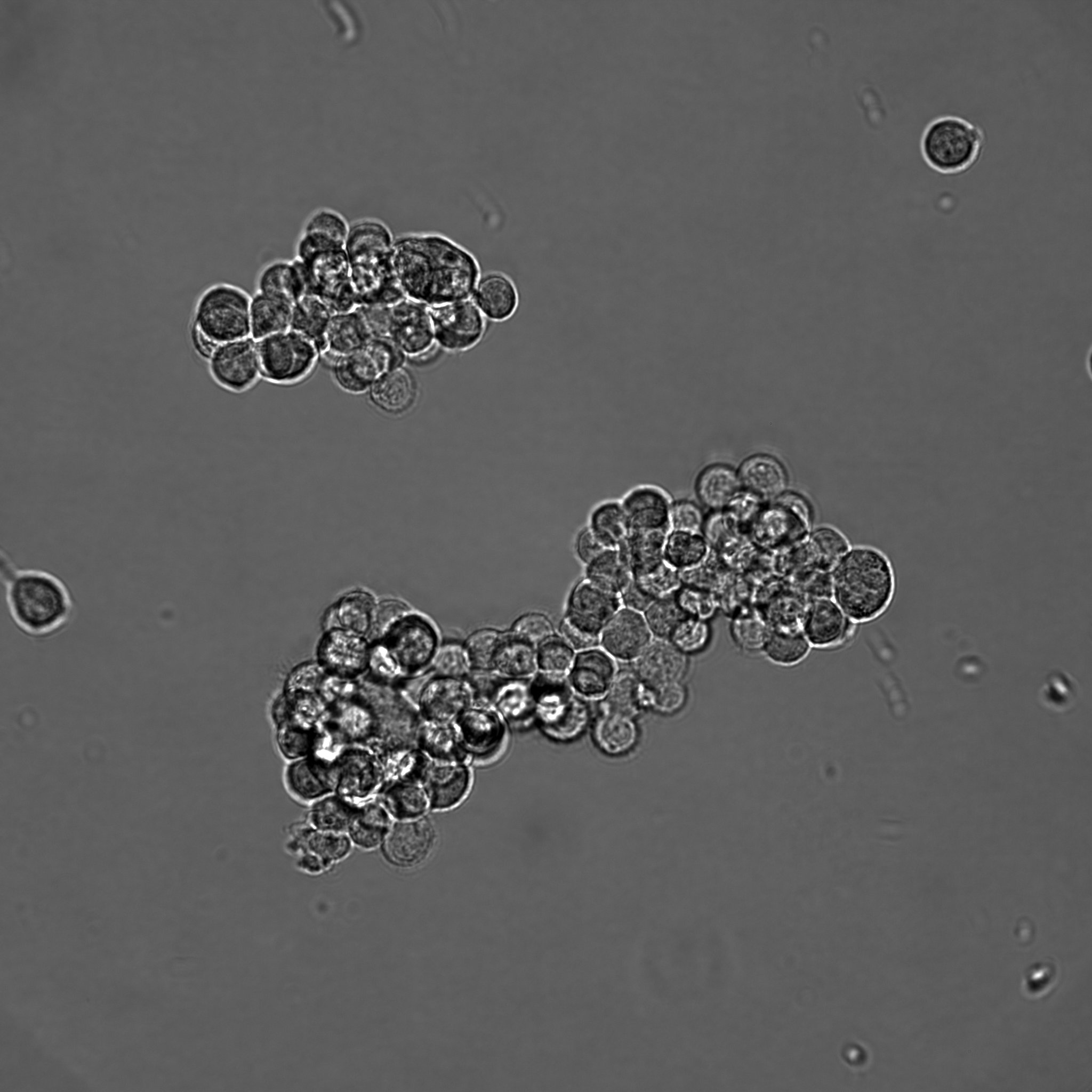

Supplement: Supplementary file 2 — Source data Fig. 1 [file 44318_2024_117_MOESM2_ESM.zip › Figure 1/1B/1B Microscopy images/40 X TRP14 KO 006 - BF.jpg]

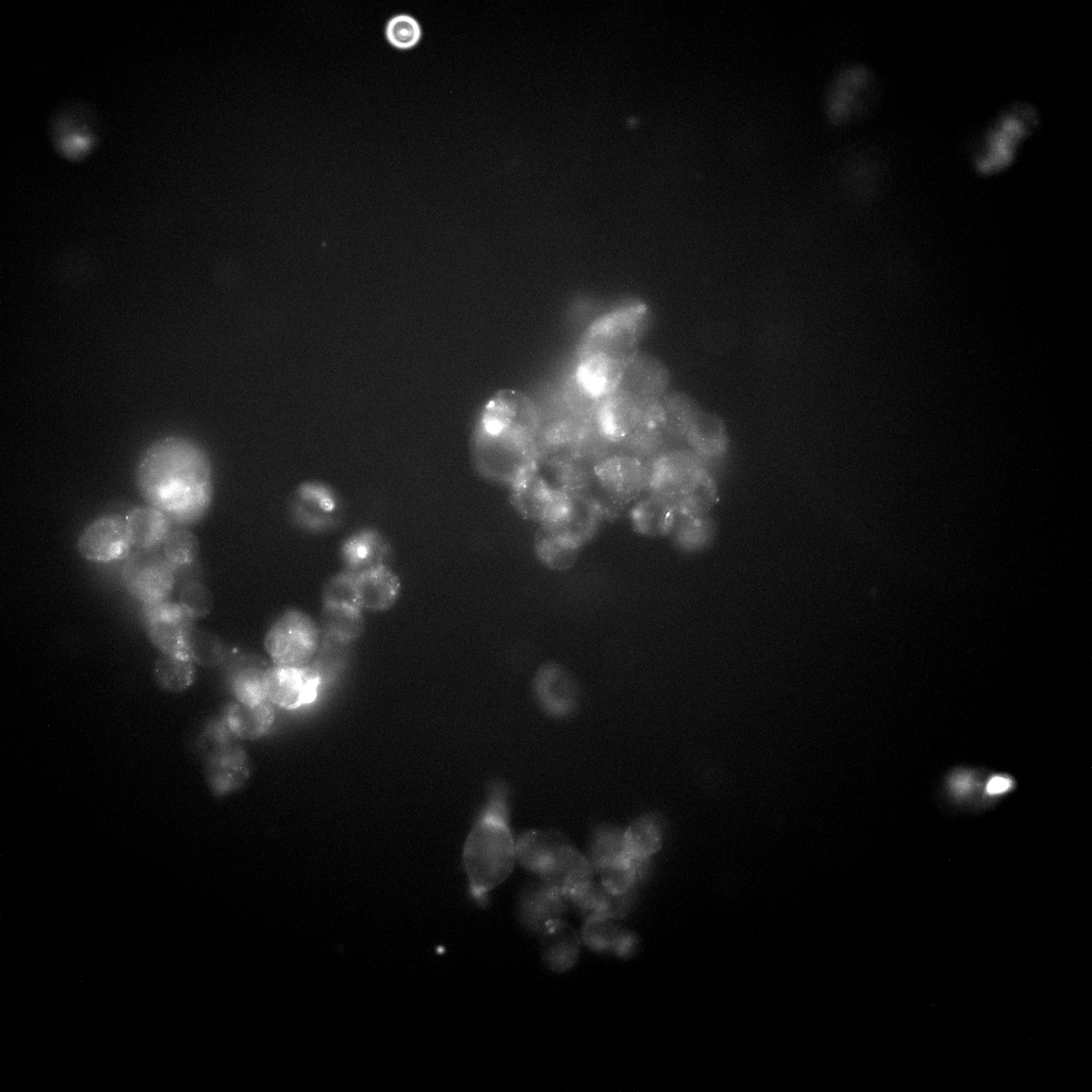

Supplement: Supplementary file 2 — Source data Fig. 1 [file 44318_2024_117_MOESM2_ESM.zip › Figure 1/1B/1B Microscopy images/40 X CT 005 - CySS.tif]

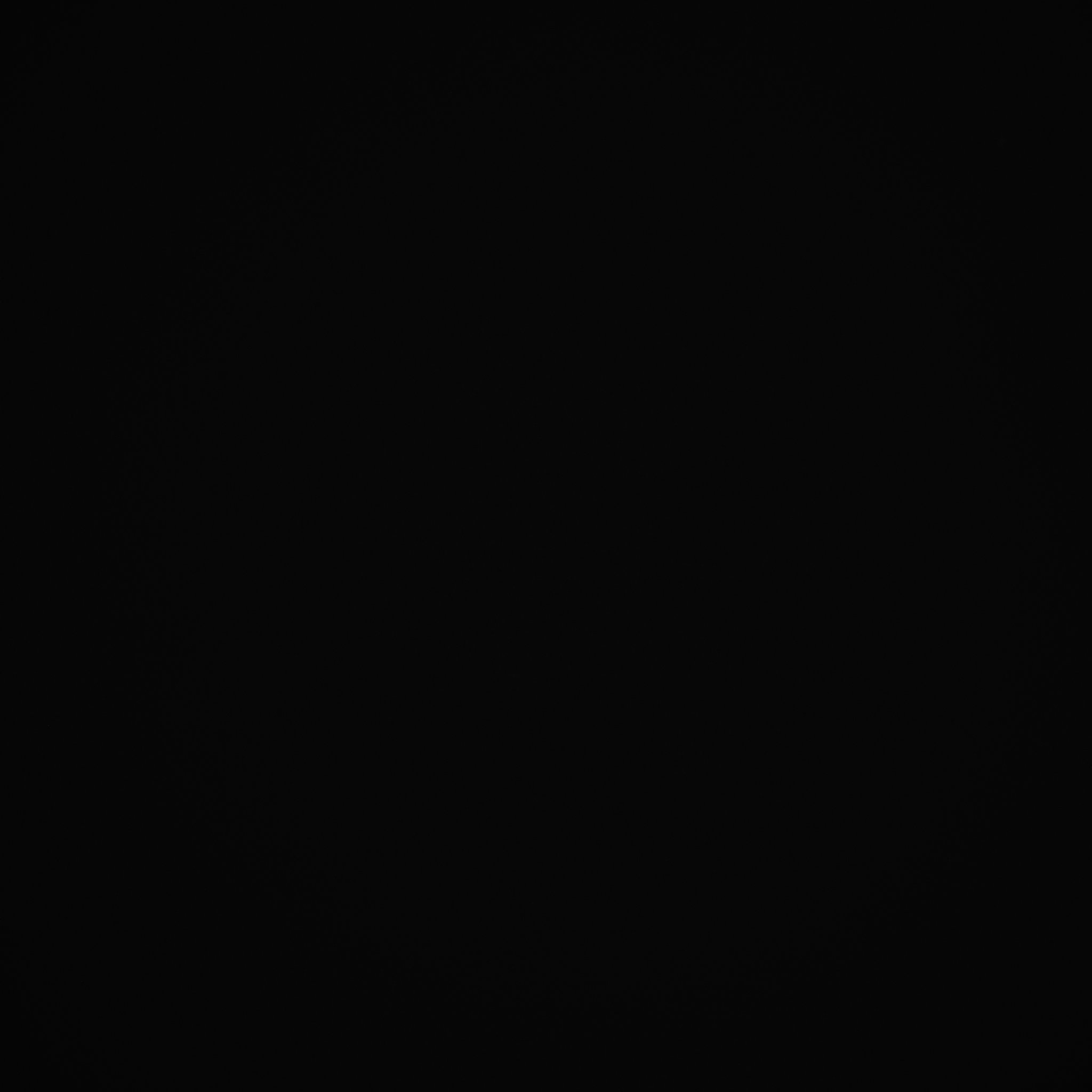

Supplement: Supplementary file 4 — Source data Fig. 3 [file 44318_2024_117_MOESM4_ESM.zip › Figure 3/3B/WT CT_CFP.jpg]

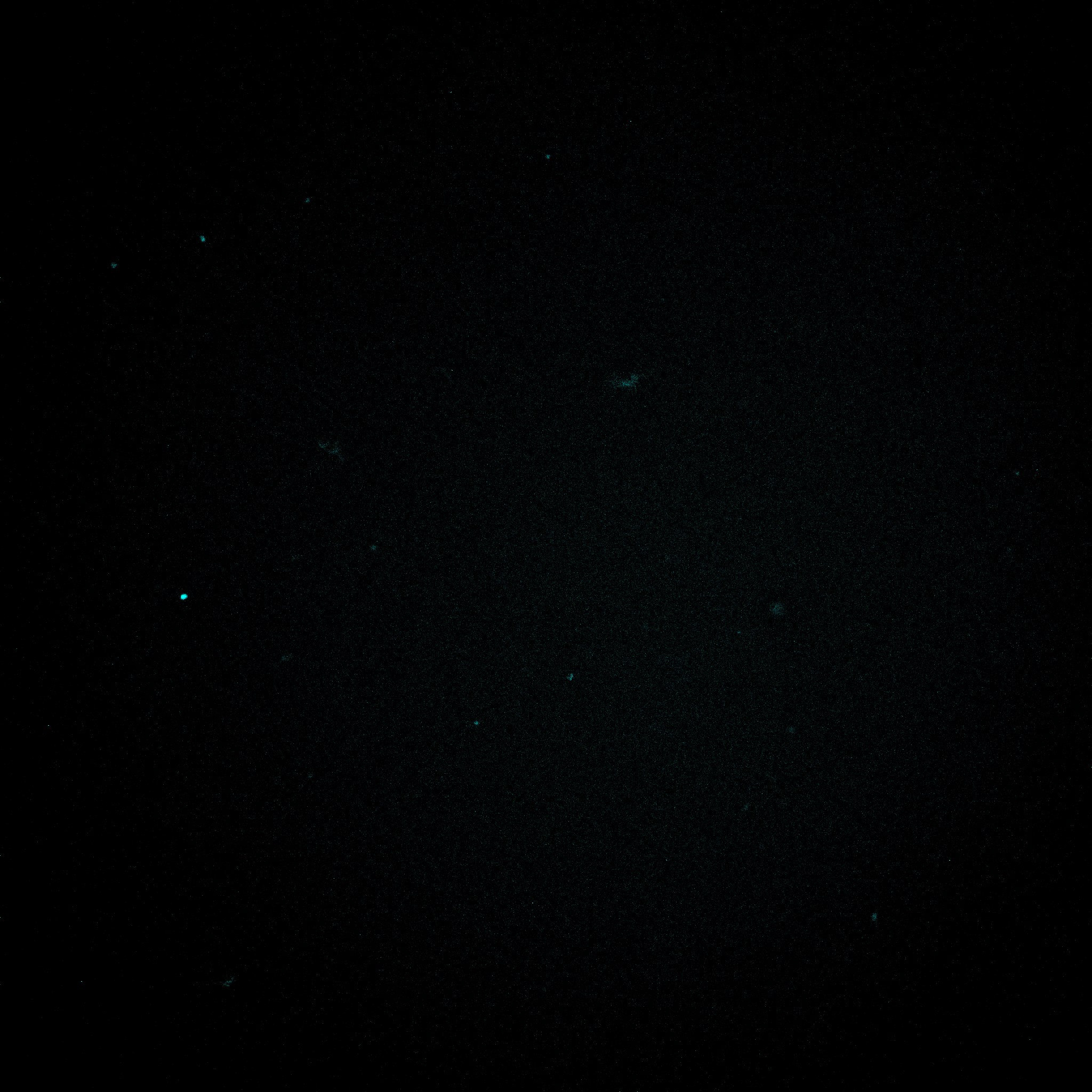

Supplement: Supplementary file 4 — Source data Fig. 3 [file 44318_2024_117_MOESM4_ESM.zip › Figure 3/3B/KO CySS_CFP.jpg]

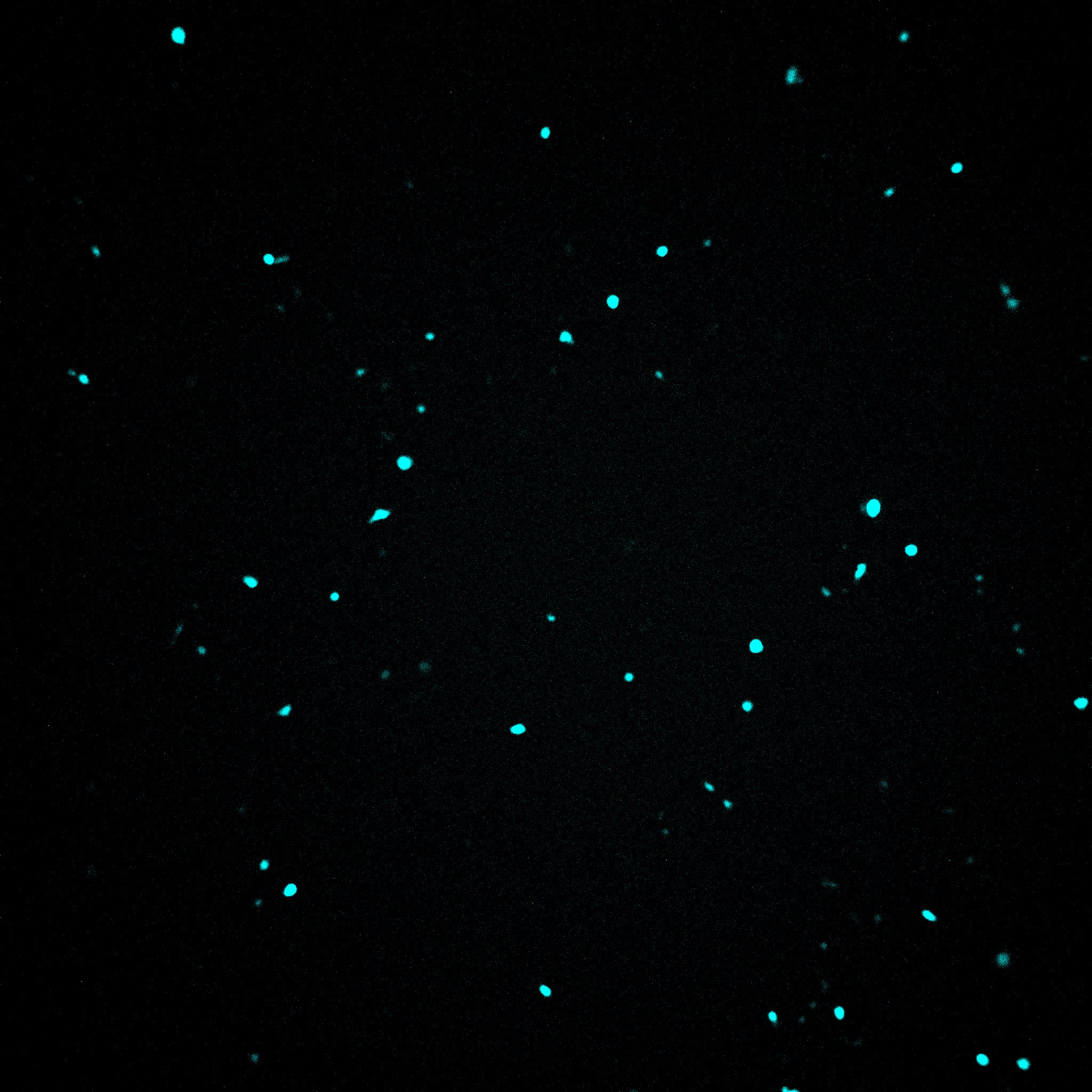

Supplement: Supplementary file 4 — Source data Fig. 3 [file 44318_2024_117_MOESM4_ESM.zip › Figure 3/3B/KO TNF_CFP.jpg]

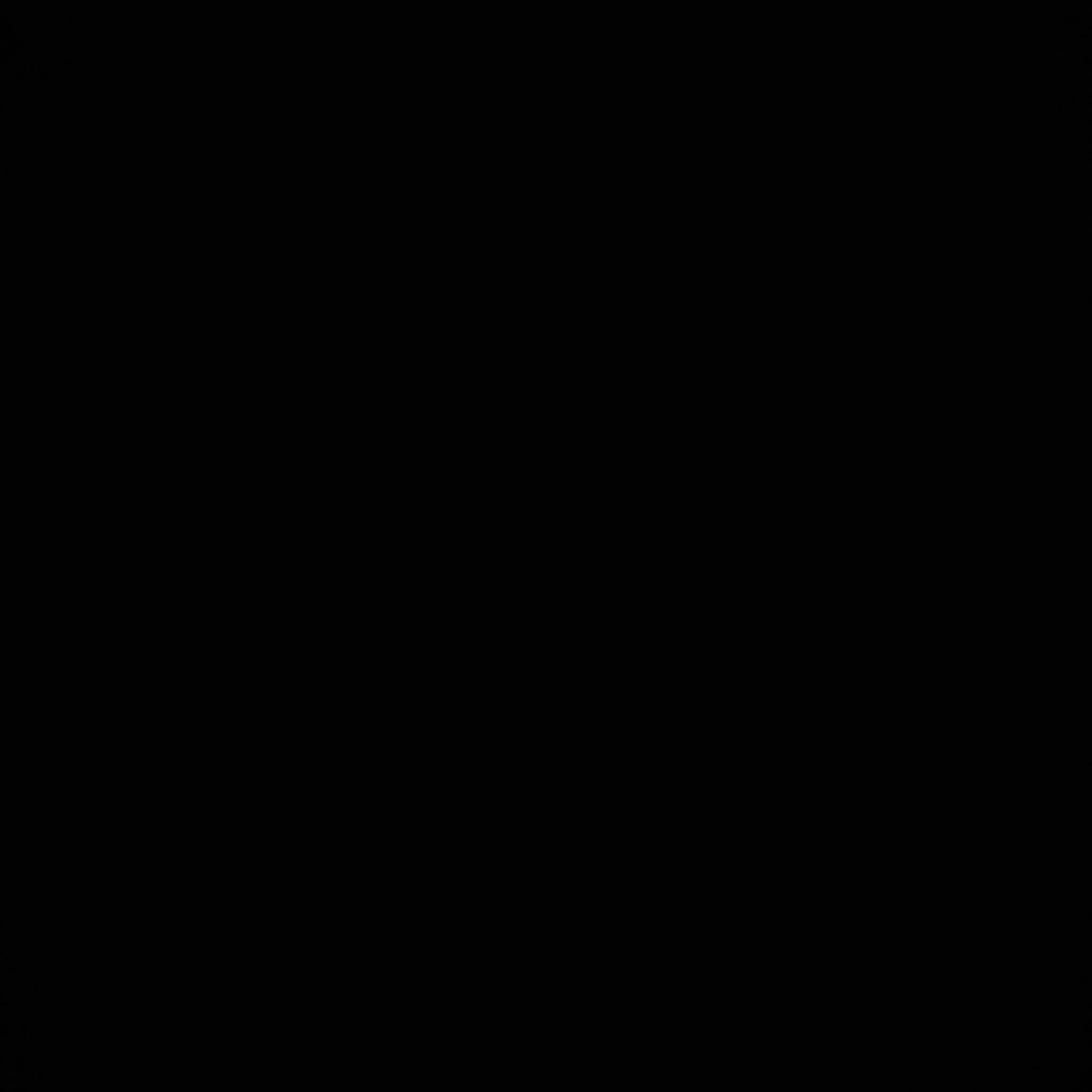

Supplement: Supplementary file 4 — Source data Fig. 3 [file 44318_2024_117_MOESM4_ESM.zip › Figure 3/3B/KO AUF_yPET.jpg]

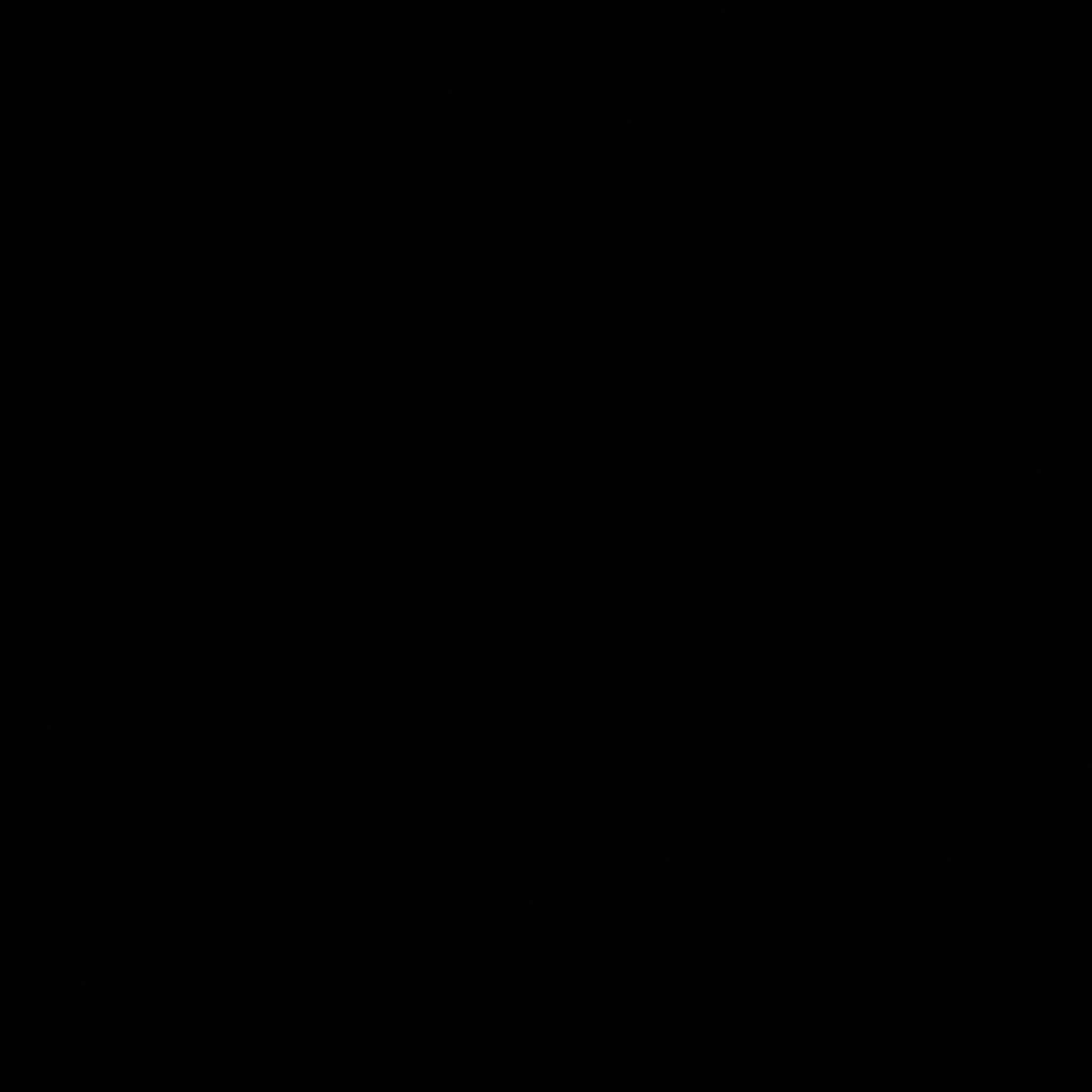

Supplement: Supplementary file 4 — Source data Fig. 3 [file 44318_2024_117_MOESM4_ESM.zip › Figure 3/3B/WT CT_yPET.jpg]

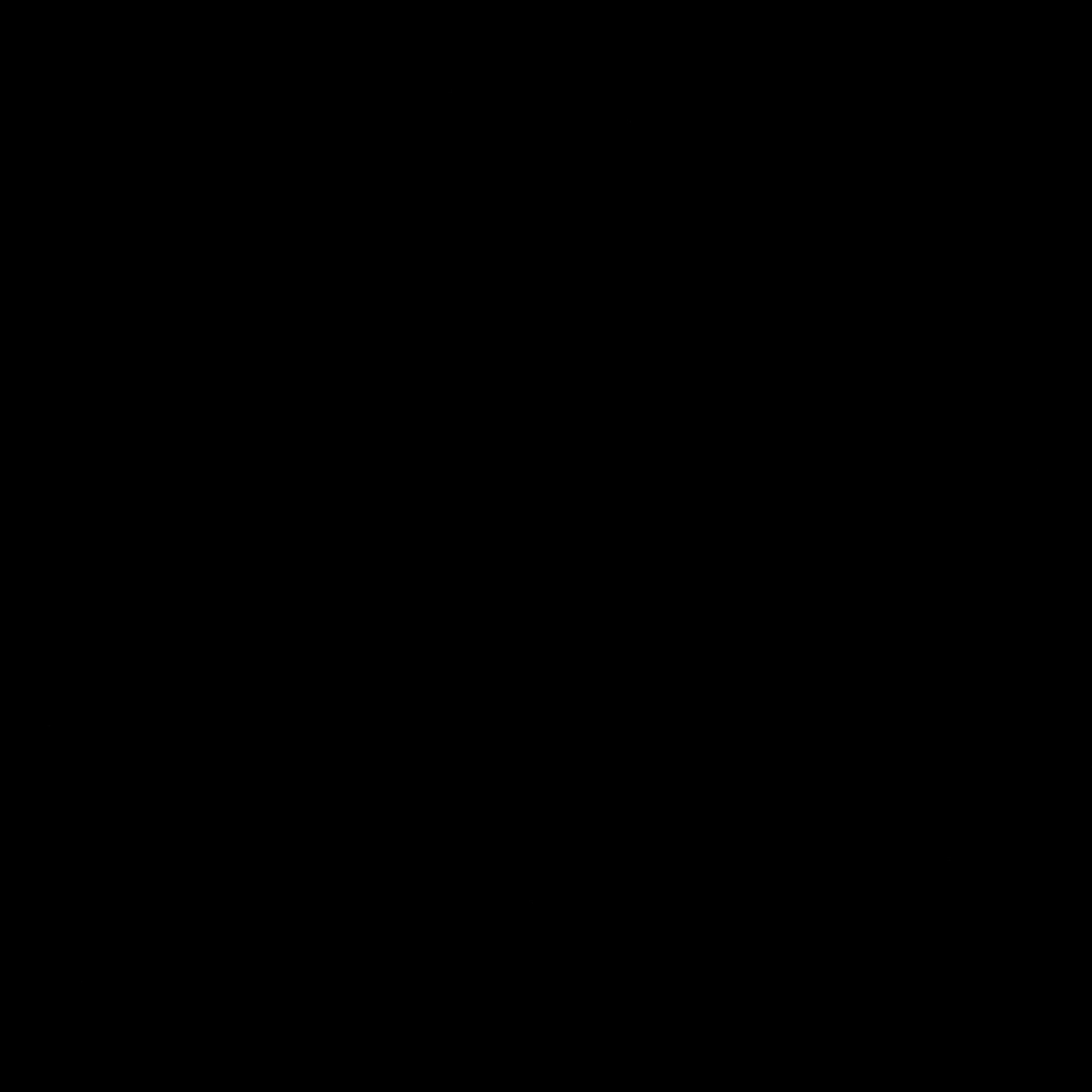

Supplement: Supplementary file 4 — Source data Fig. 3 [file 44318_2024_117_MOESM4_ESM.zip › Figure 3/3B/KO TNF_yPET.jpg]

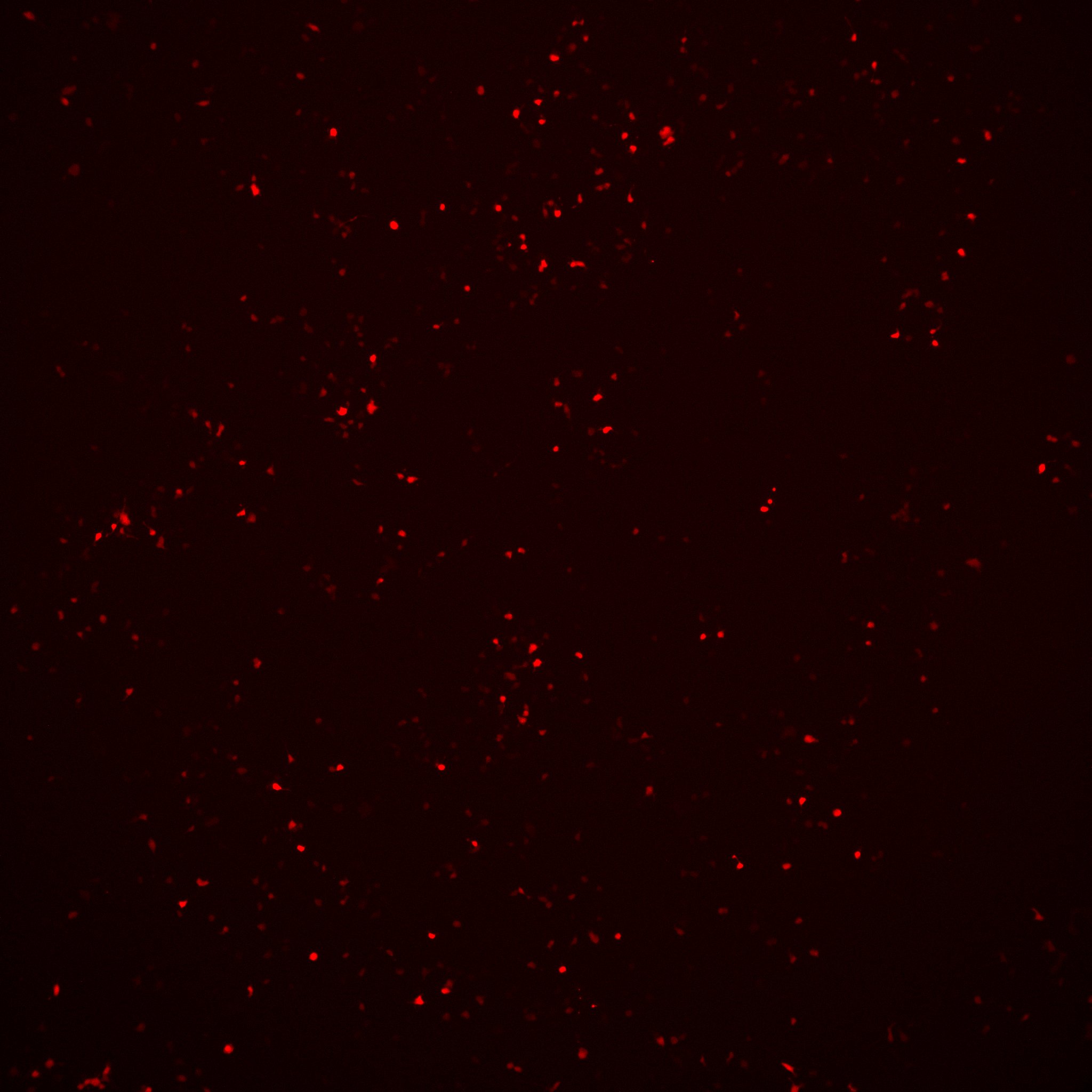

Supplement: Supplementary file 4 — Source data Fig. 3 [file 44318_2024_117_MOESM4_ESM.zip › Figure 3/3B/WT AUF_mCherry.jpg]

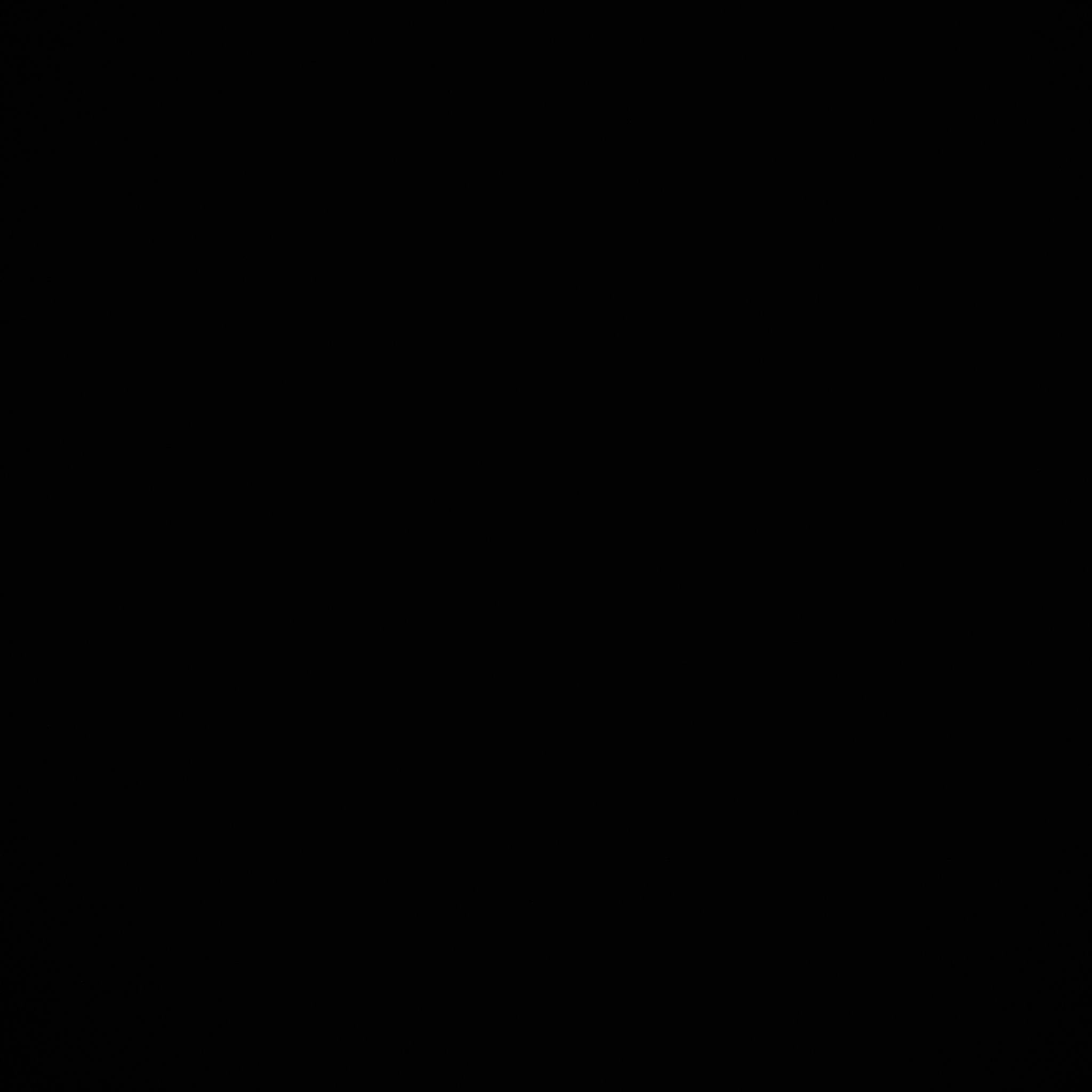

Supplement: Supplementary file 4 — Source data Fig. 3 [file 44318_2024_117_MOESM4_ESM.zip › Figure 3/3B/KO CySS_yPET.jpg]

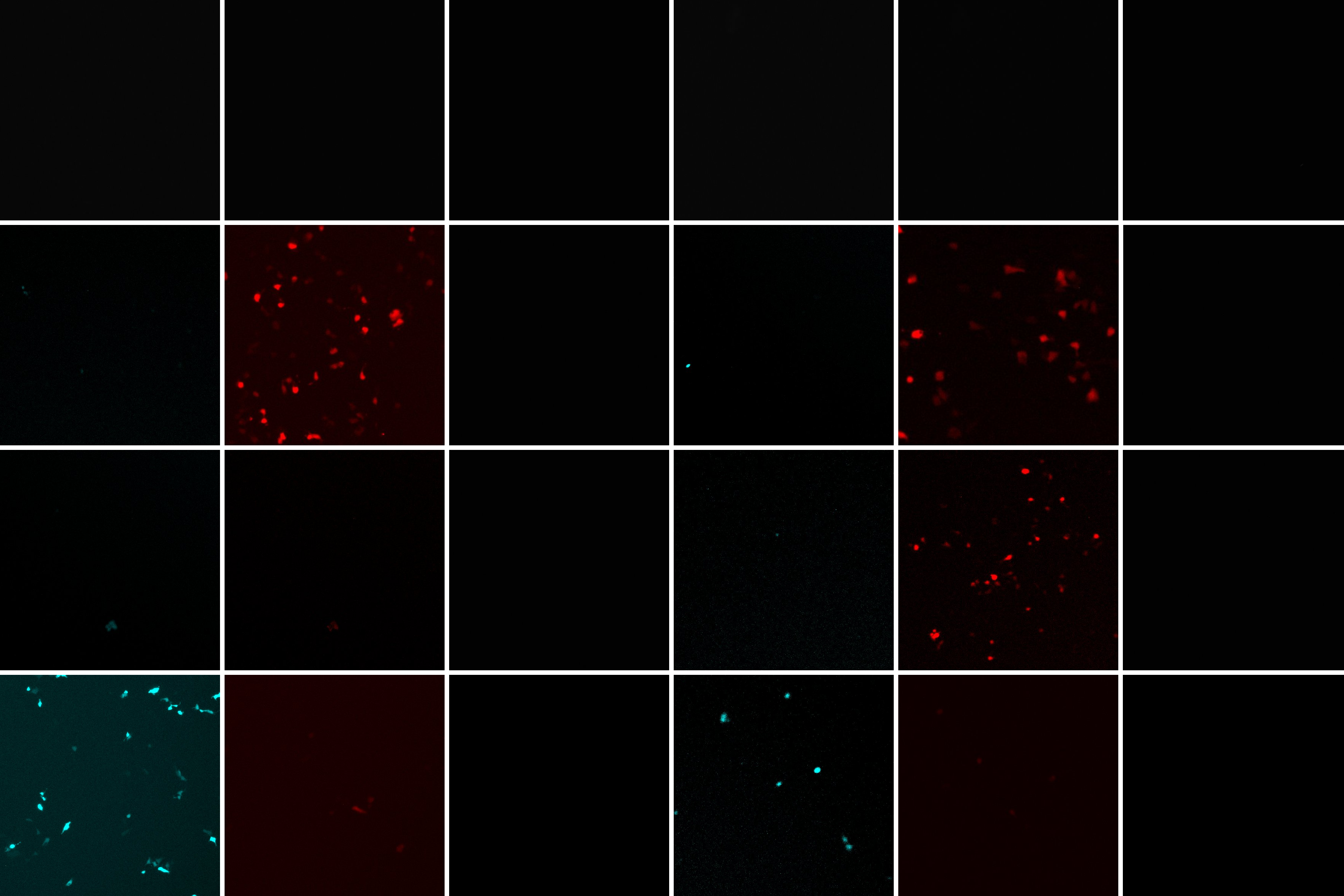

Supplement: Supplementary file 4 — Source data Fig. 3 [file 44318_2024_117_MOESM4_ESM.zip › Figure 3/3B/3B - pTRAF.tif]

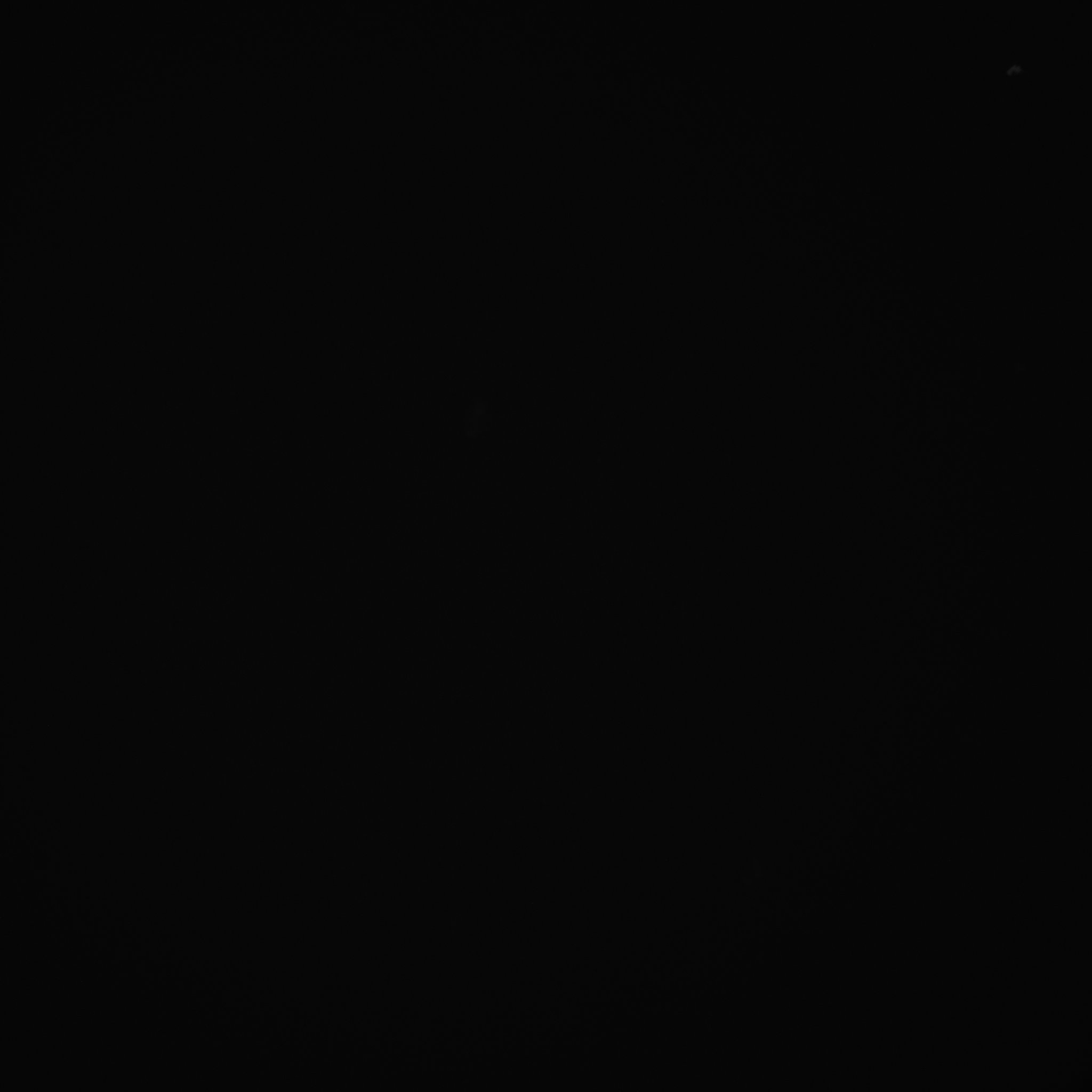

Supplement: Supplementary file 4 — Source data Fig. 3 [file 44318_2024_117_MOESM4_ESM.zip › Figure 3/3B/KO CT_CFP.jpg]

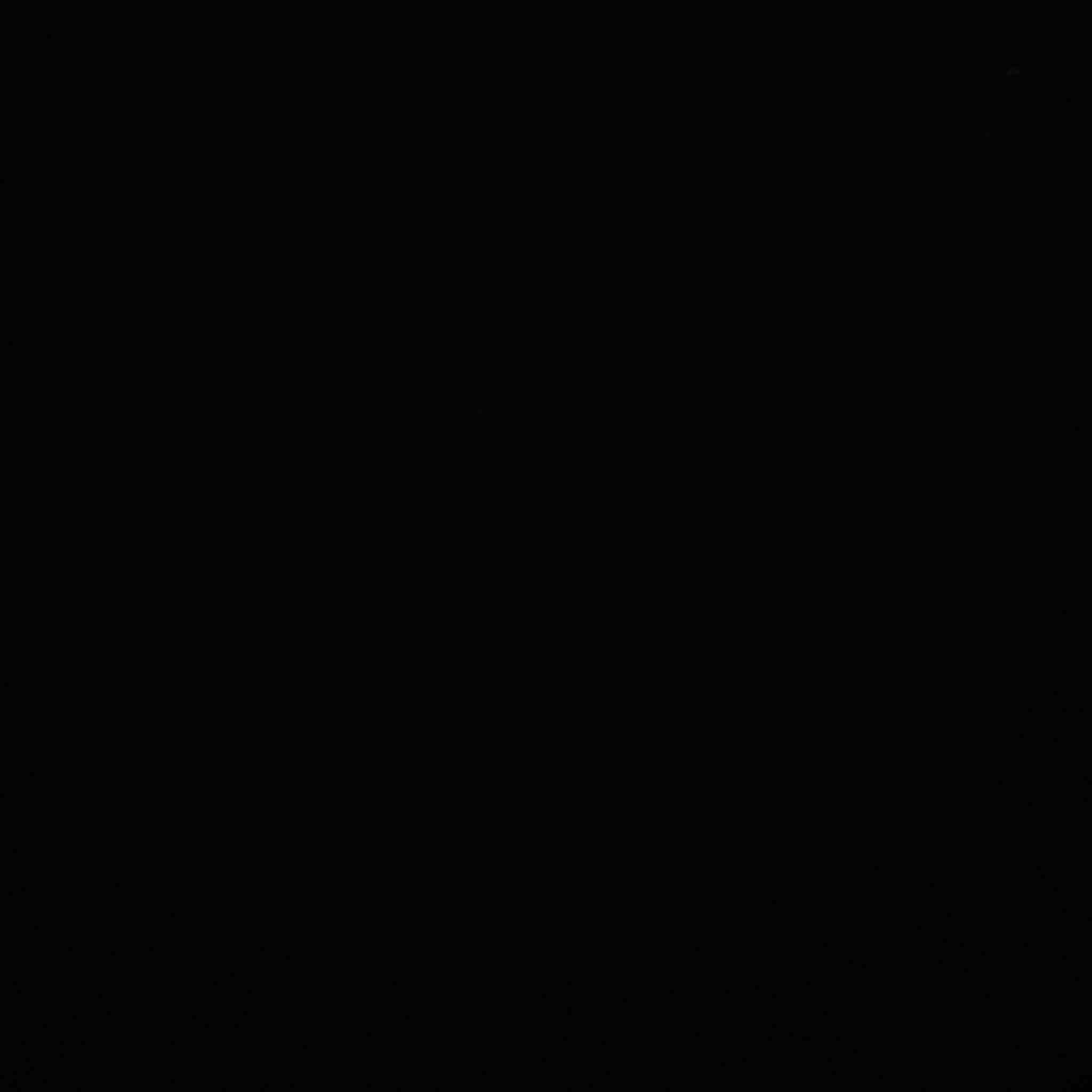

Supplement: Supplementary file 4 — Source data Fig. 3 [file 44318_2024_117_MOESM4_ESM.zip › Figure 3/3B/KO CT_mCherry.jpg]

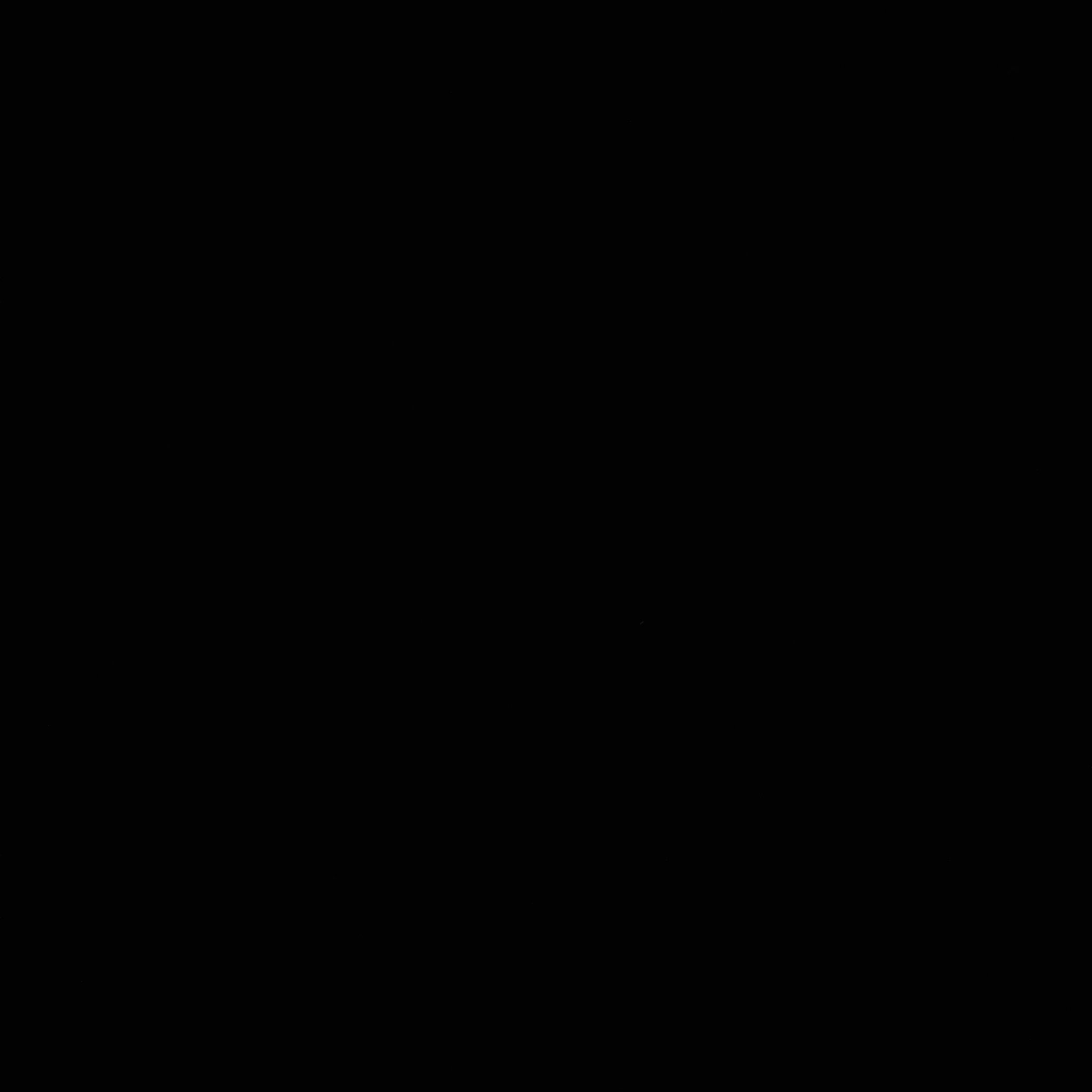

Supplement: Supplementary file 4 — Source data Fig. 3 [file 44318_2024_117_MOESM4_ESM.zip › Figure 3/3B/KO CT_yPET.jpg]

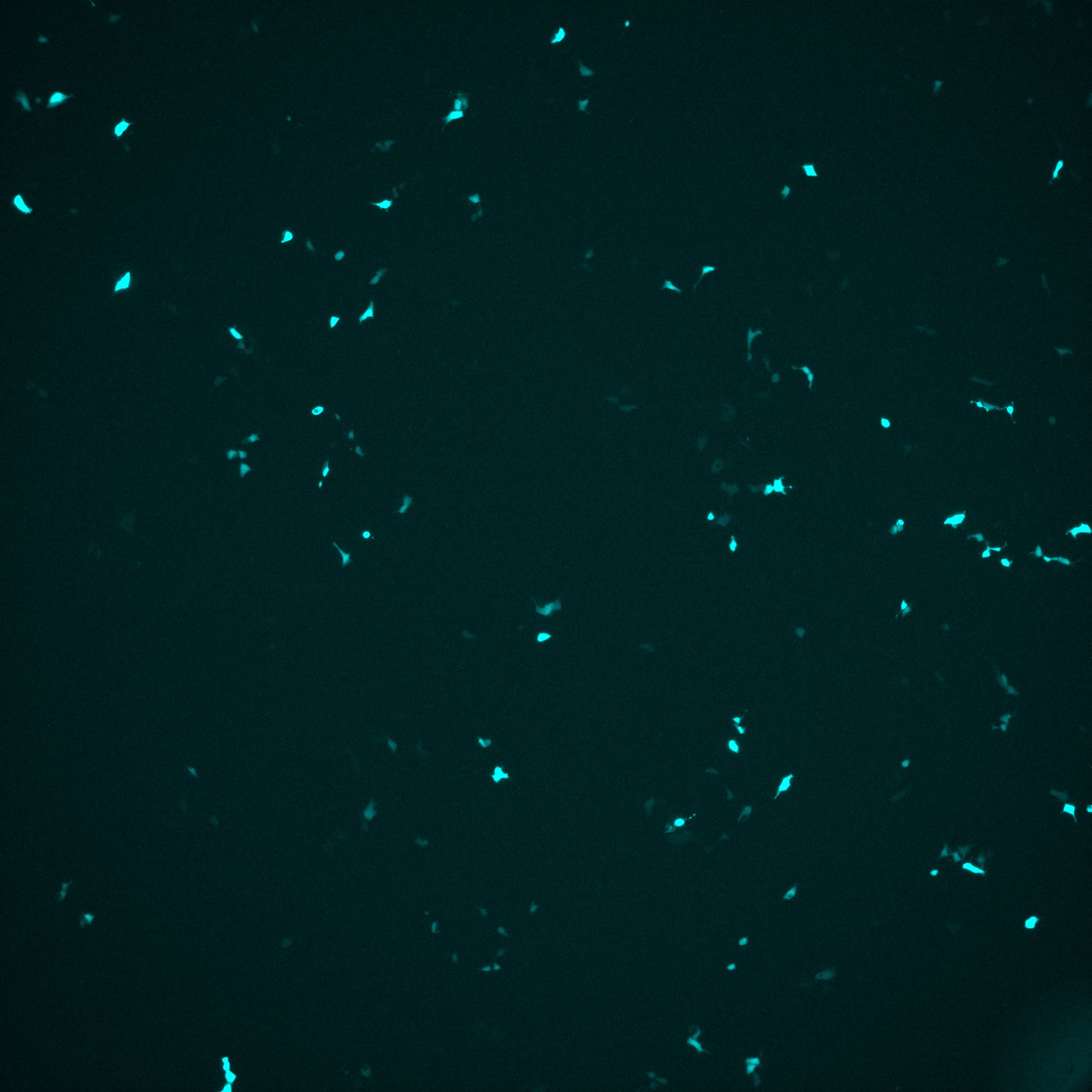

Supplement: Supplementary file 4 — Source data Fig. 3 [file 44318_2024_117_MOESM4_ESM.zip › Figure 3/3B/WT TNF_CFP.jpg]

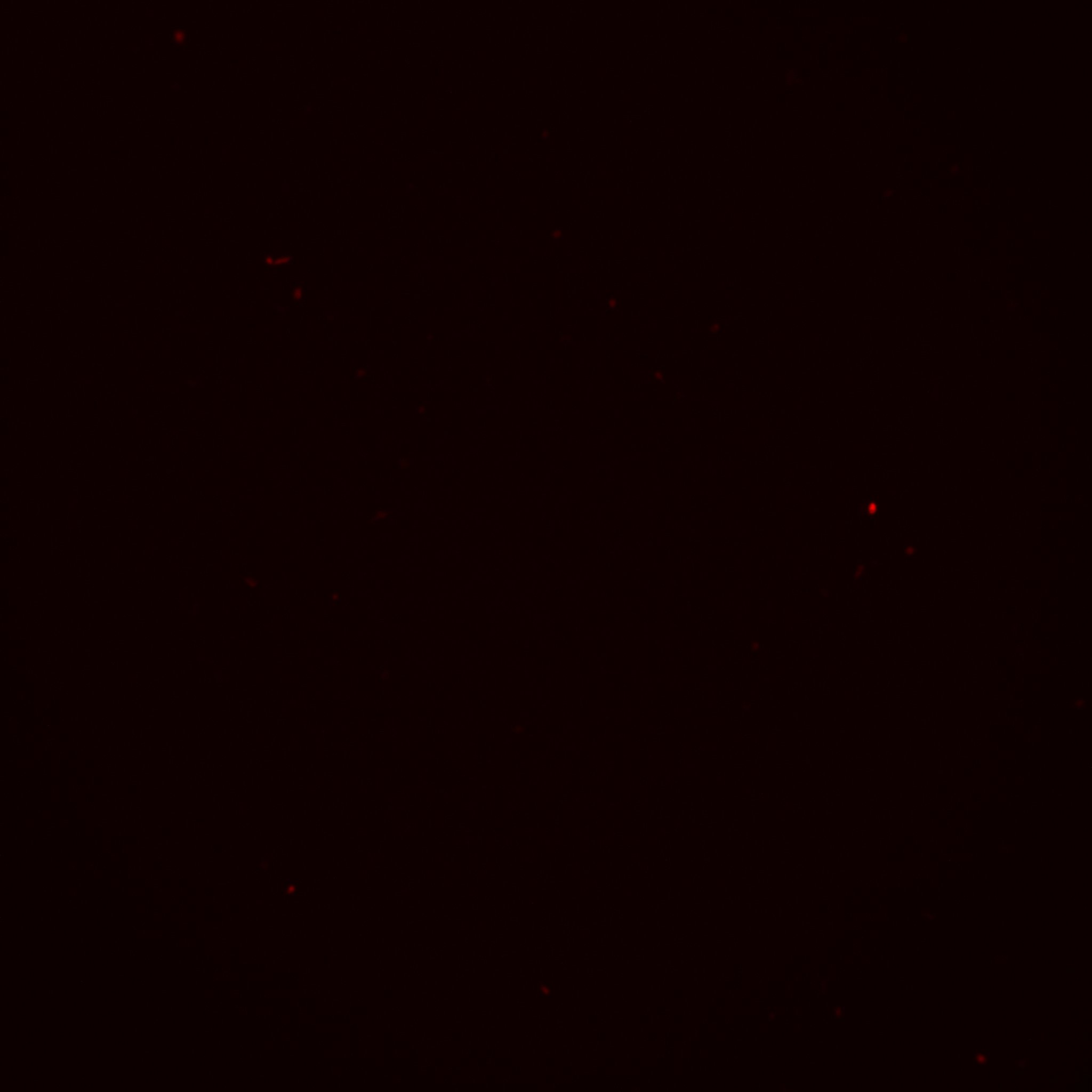

Supplement: Supplementary file 4 — Source data Fig. 3 [file 44318_2024_117_MOESM4_ESM.zip › Figure 3/3B/KO TNF_mCherry.jpg]

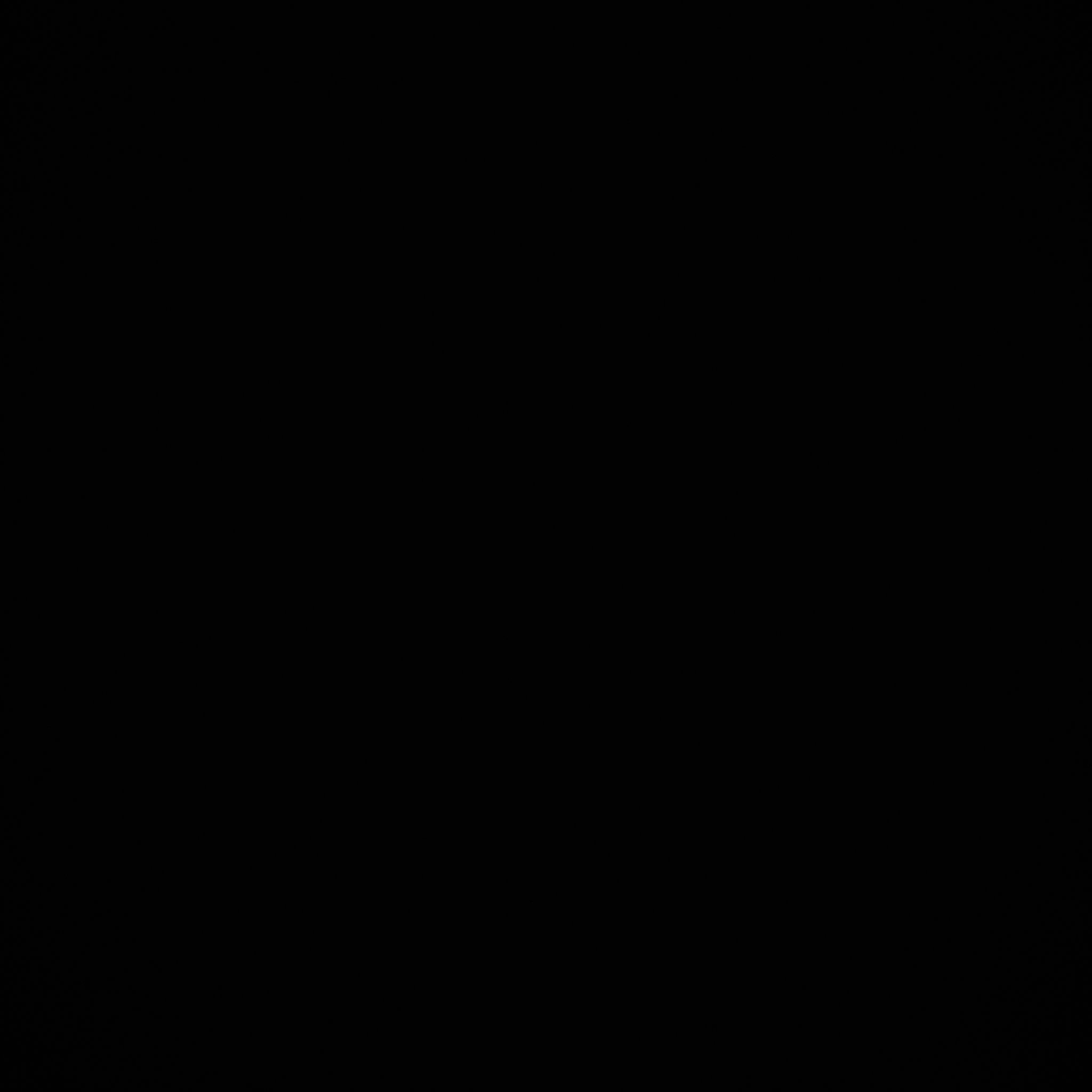

Supplement: Supplementary file 4 — Source data Fig. 3 [file 44318_2024_117_MOESM4_ESM.zip › Figure 3/3B/WT AUF_yPET.jpg]

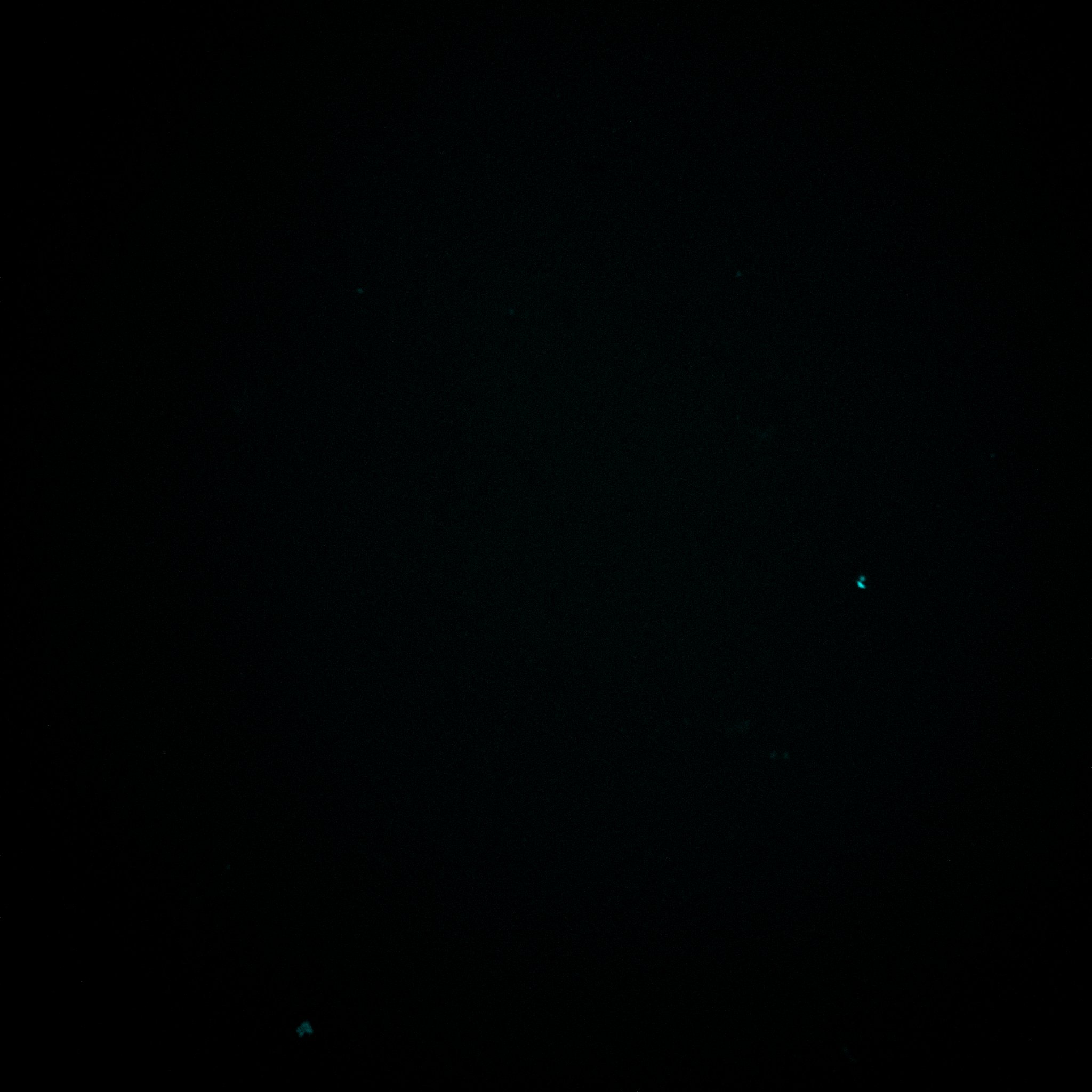

Supplement: Supplementary file 4 — Source data Fig. 3 [file 44318_2024_117_MOESM4_ESM.zip › Figure 3/3B/WT CySS_CFP.jpg]

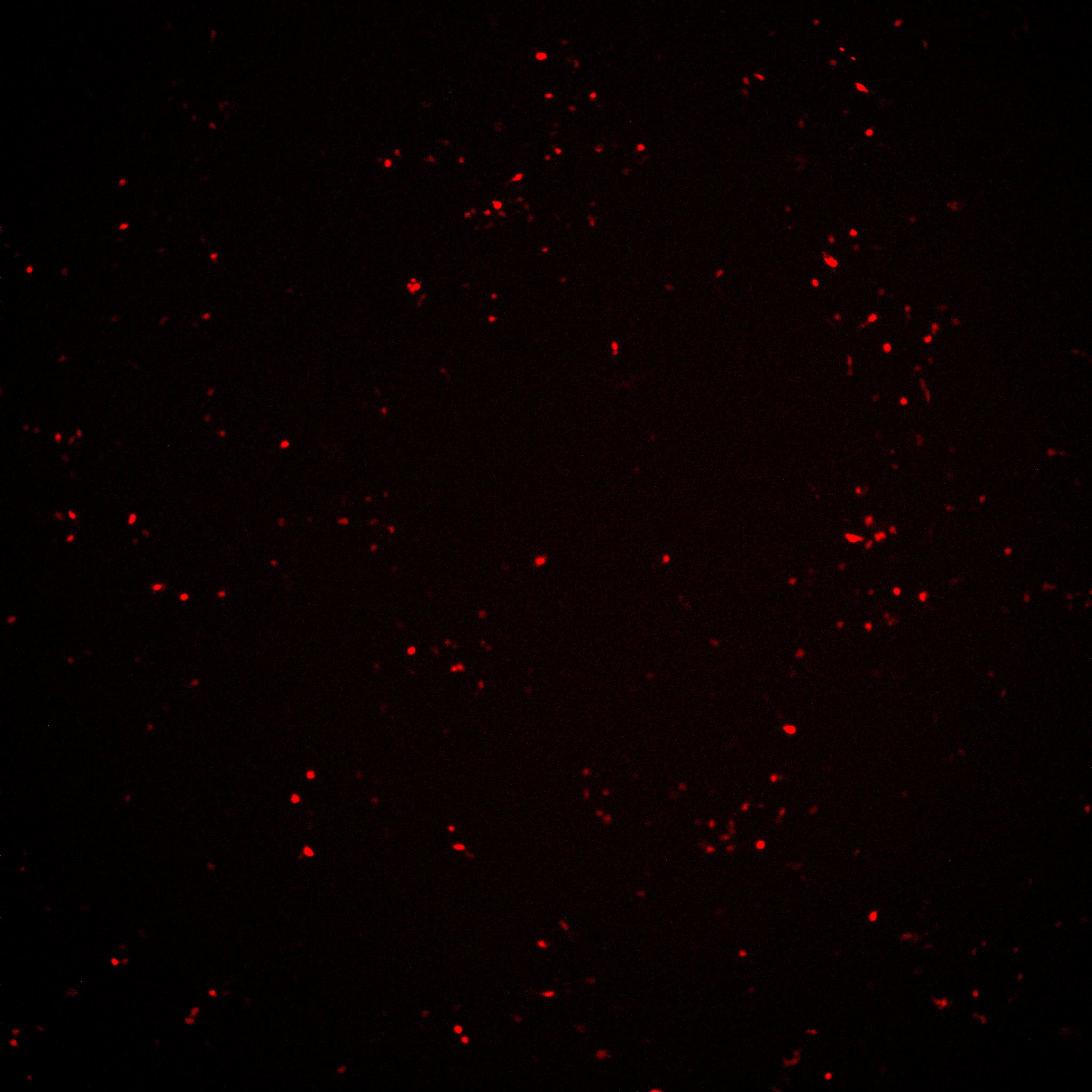

Supplement: Supplementary file 4 — Source data Fig. 3 [file 44318_2024_117_MOESM4_ESM.zip › Figure 3/3B/KO CySS_mCherry.jpg]

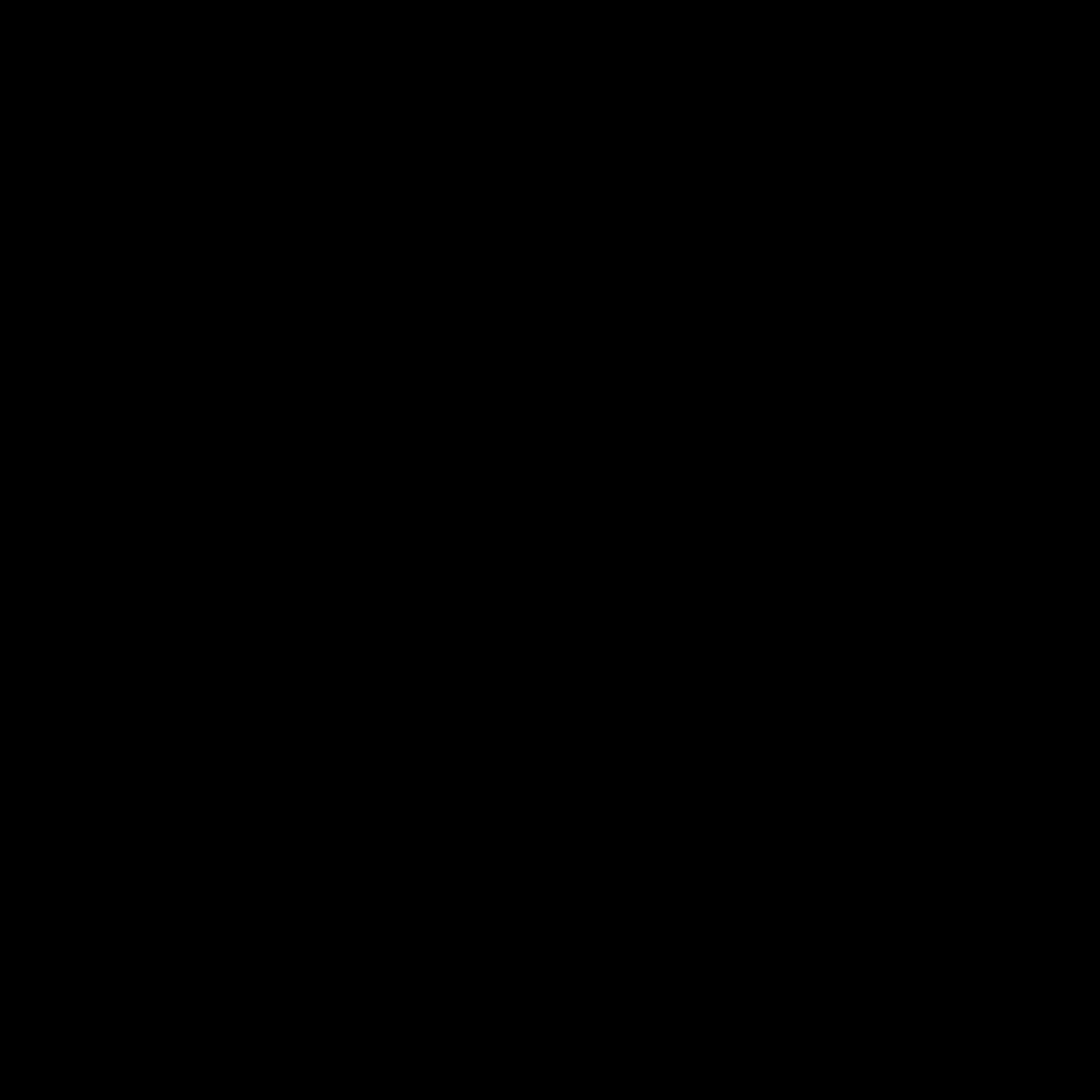

Supplement: Supplementary file 4 — Source data Fig. 3 [file 44318_2024_117_MOESM4_ESM.zip › Figure 3/3B/WT TNF_yPET.jpg]

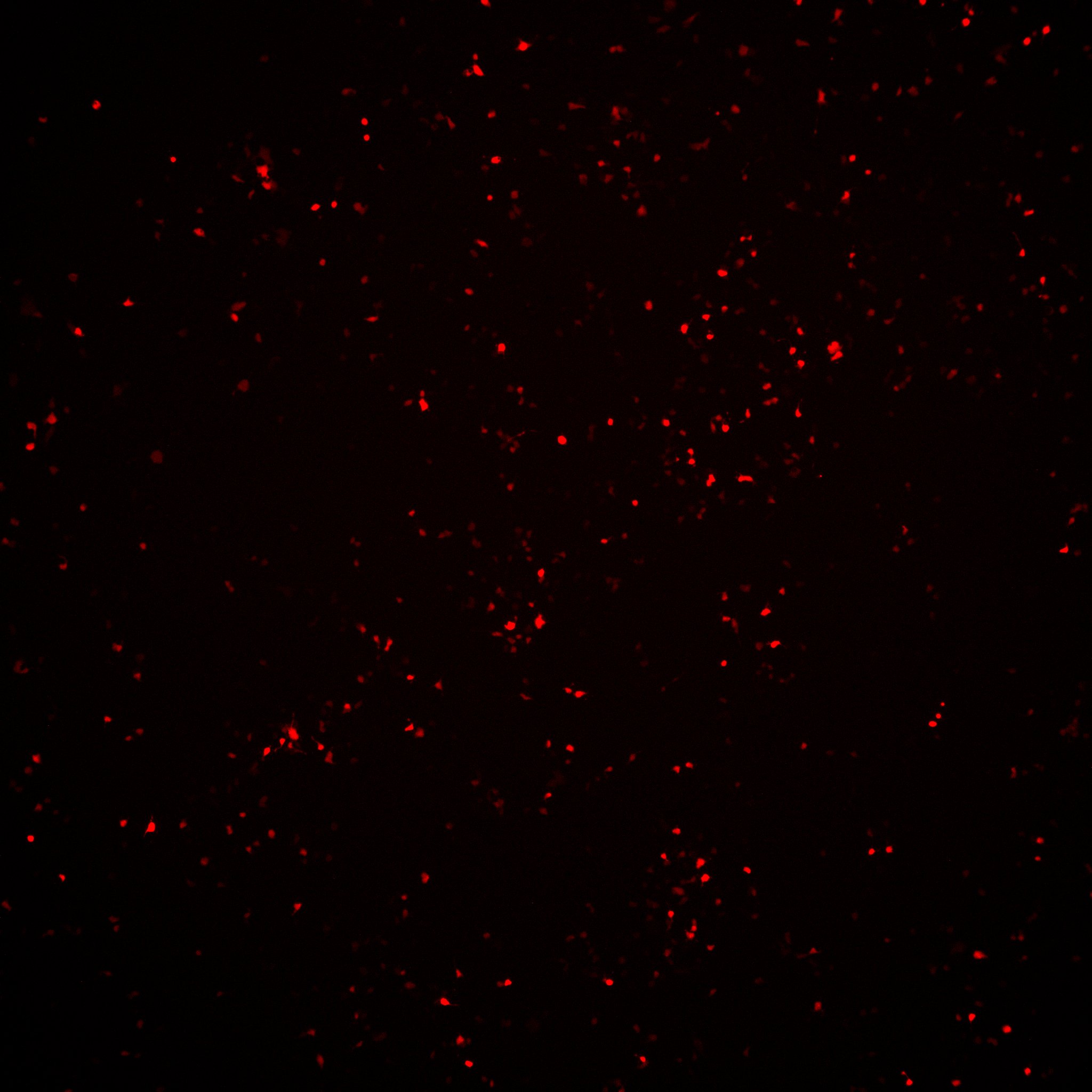

Supplement: Supplementary file 4 — Source data Fig. 3 [file 44318_2024_117_MOESM4_ESM.zip › Figure 3/3B/KO AUF_mCherry.jpg]

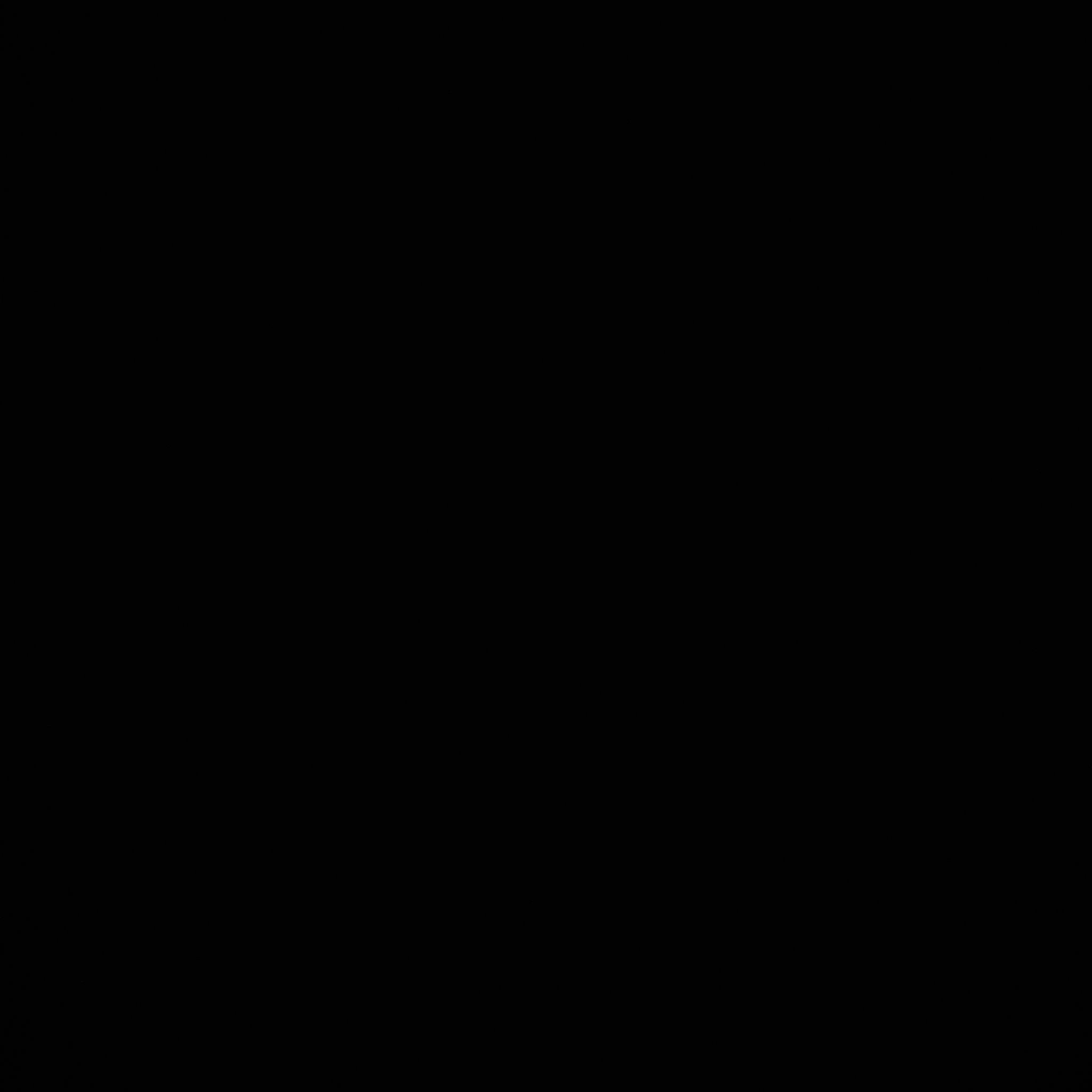

Supplement: Supplementary file 4 — Source data Fig. 3 [file 44318_2024_117_MOESM4_ESM.zip › Figure 3/3B/WT CySS_yPET.jpg]

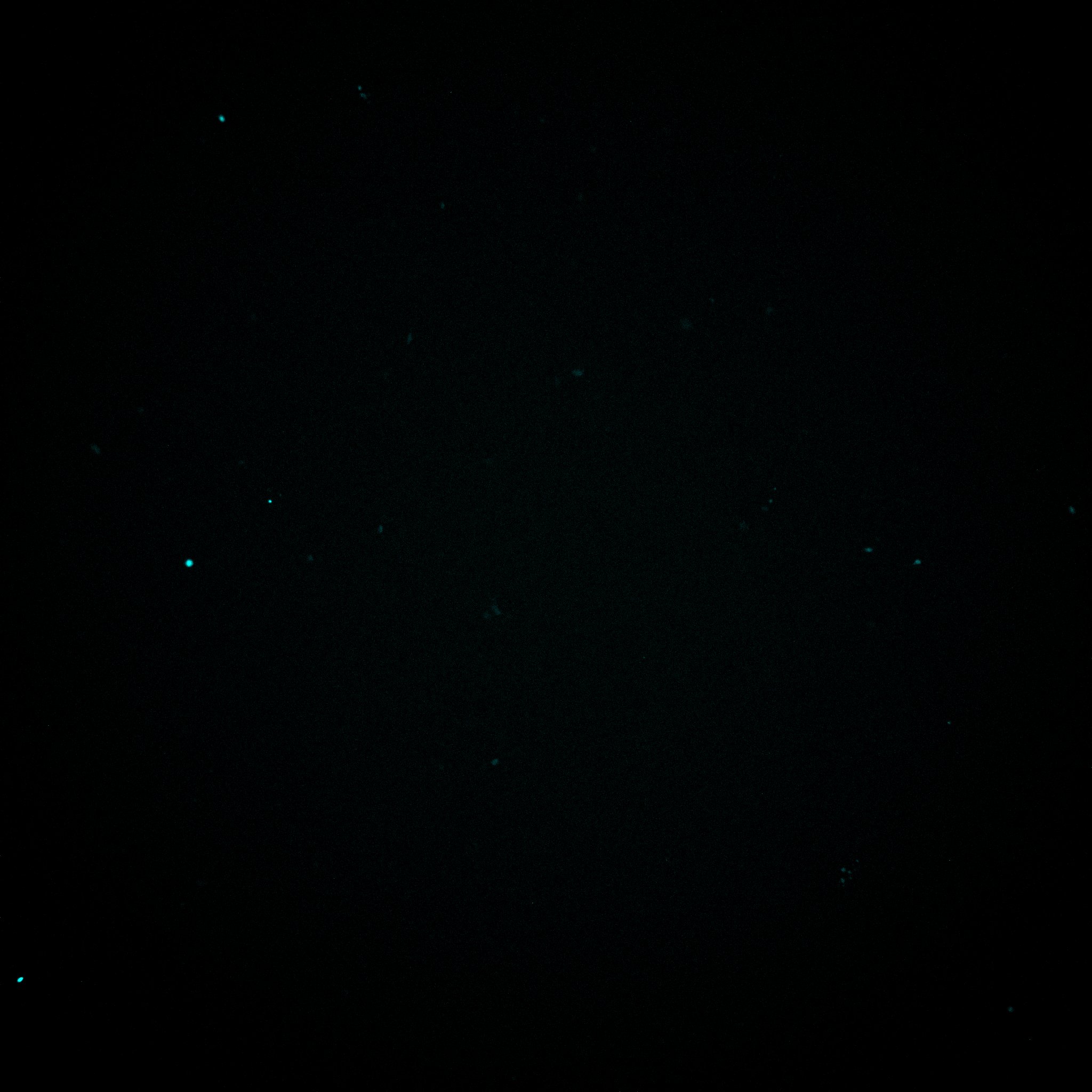

Supplement: Supplementary file 4 — Source data Fig. 3 [file 44318_2024_117_MOESM4_ESM.zip › Figure 3/3B/WT AUF_CFP.jpg]

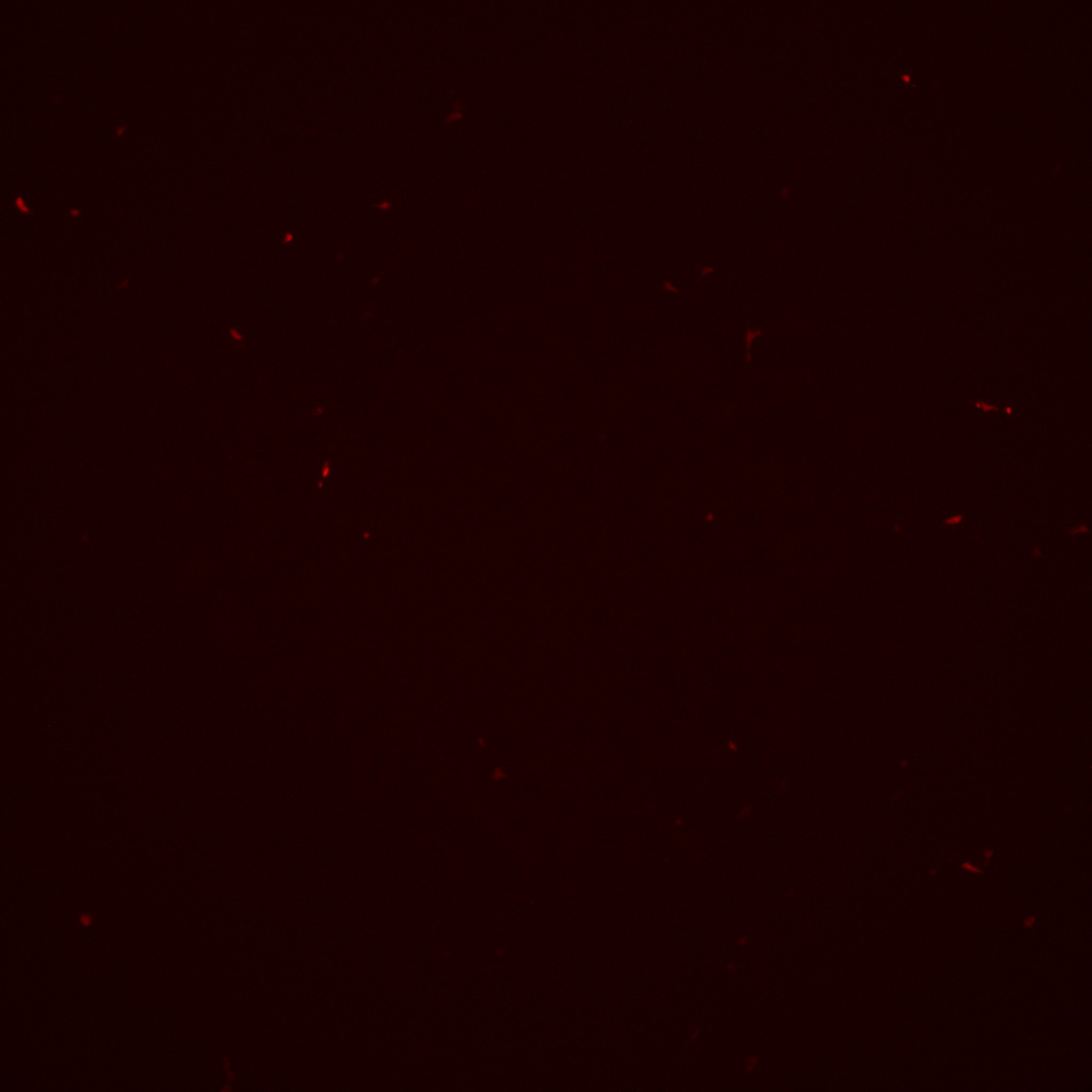

Supplement: Supplementary file 4 — Source data Fig. 3 [file 44318_2024_117_MOESM4_ESM.zip › Figure 3/3B/WT TNF_mCherry.jpg]

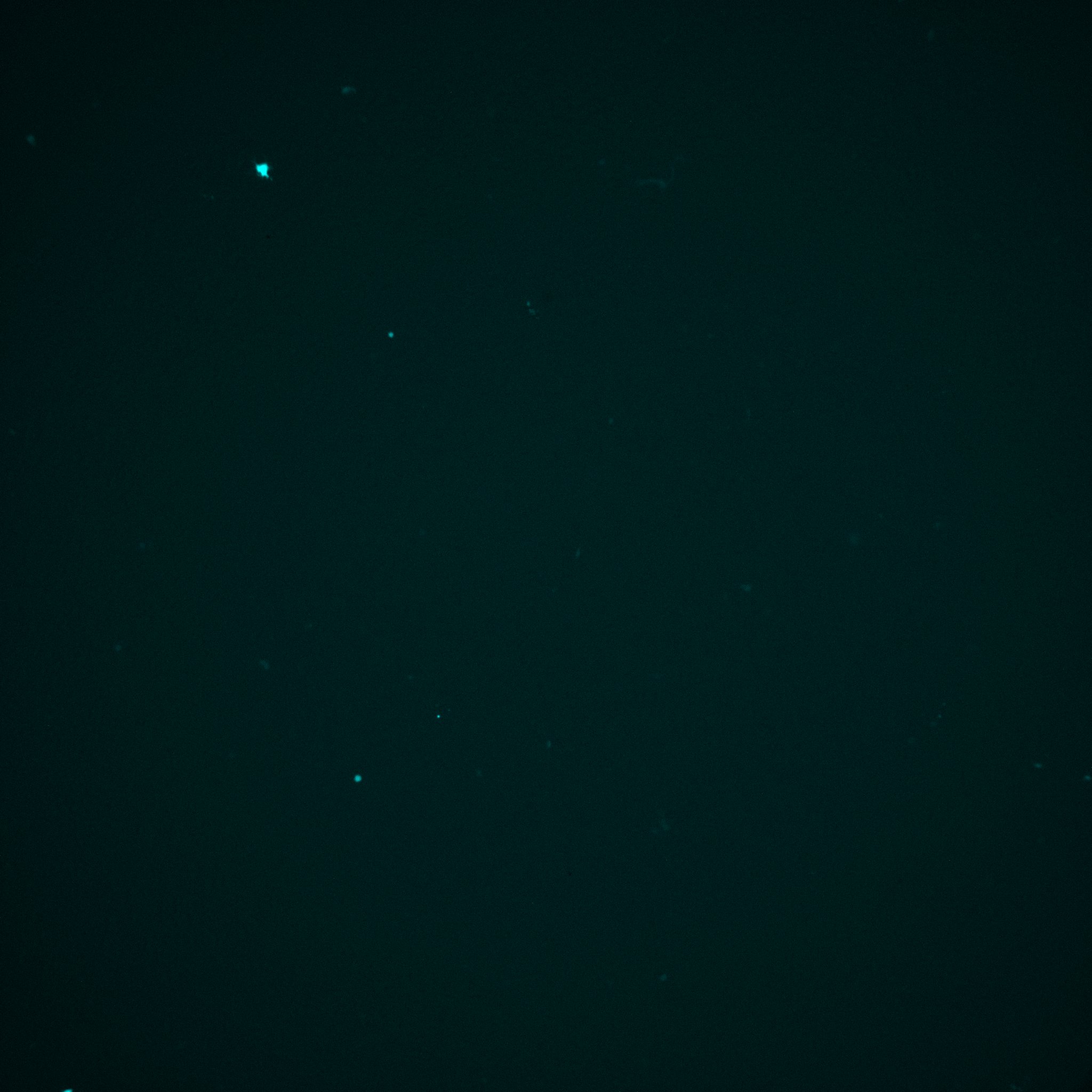

Supplement: Supplementary file 4 — Source data Fig. 3 [file 44318_2024_117_MOESM4_ESM.zip › Figure 3/3B/KO AUF_CFP.jpg]

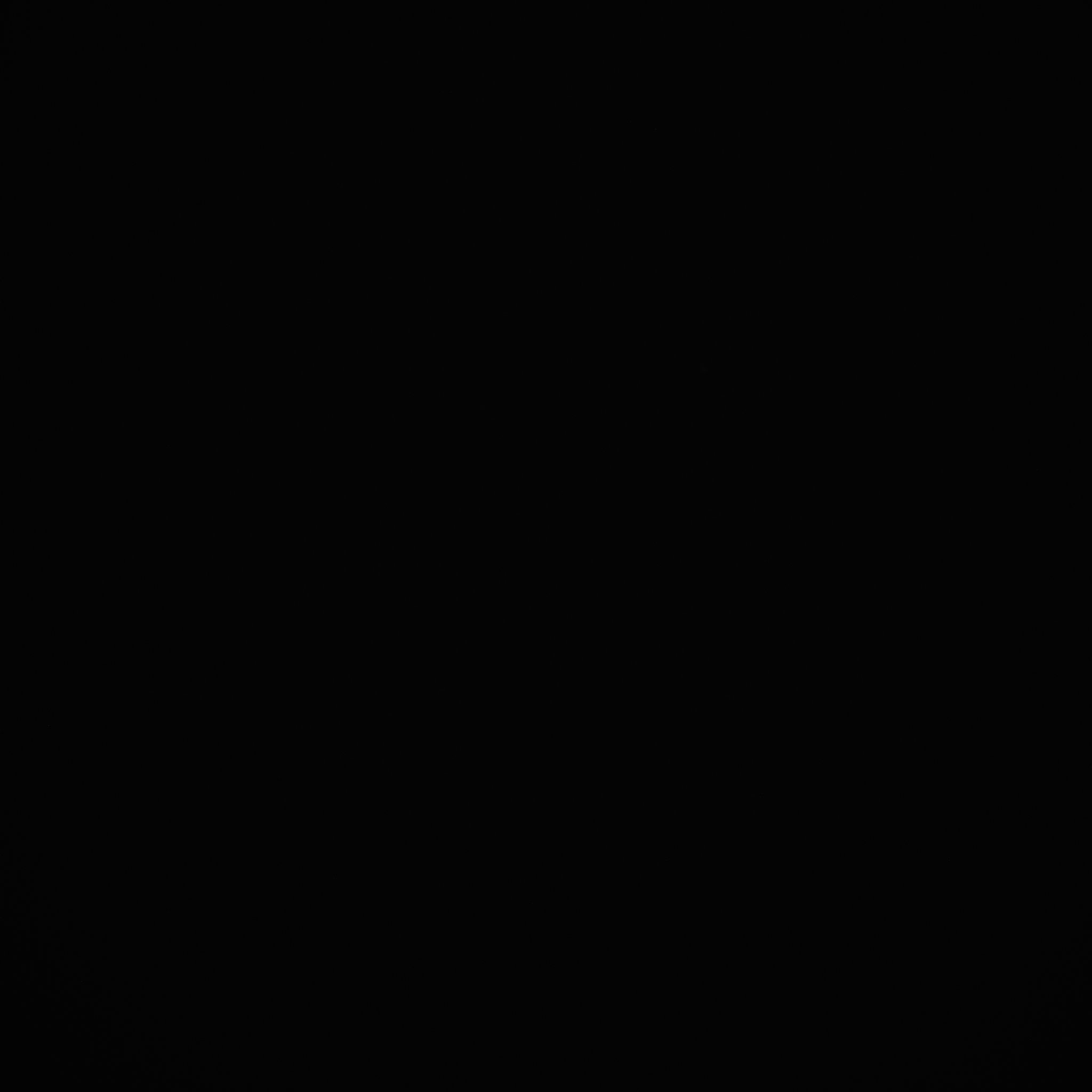

Supplement: Supplementary file 4 — Source data Fig. 3 [file 44318_2024_117_MOESM4_ESM.zip › Figure 3/3B/WT CT_mCherry.jpg]

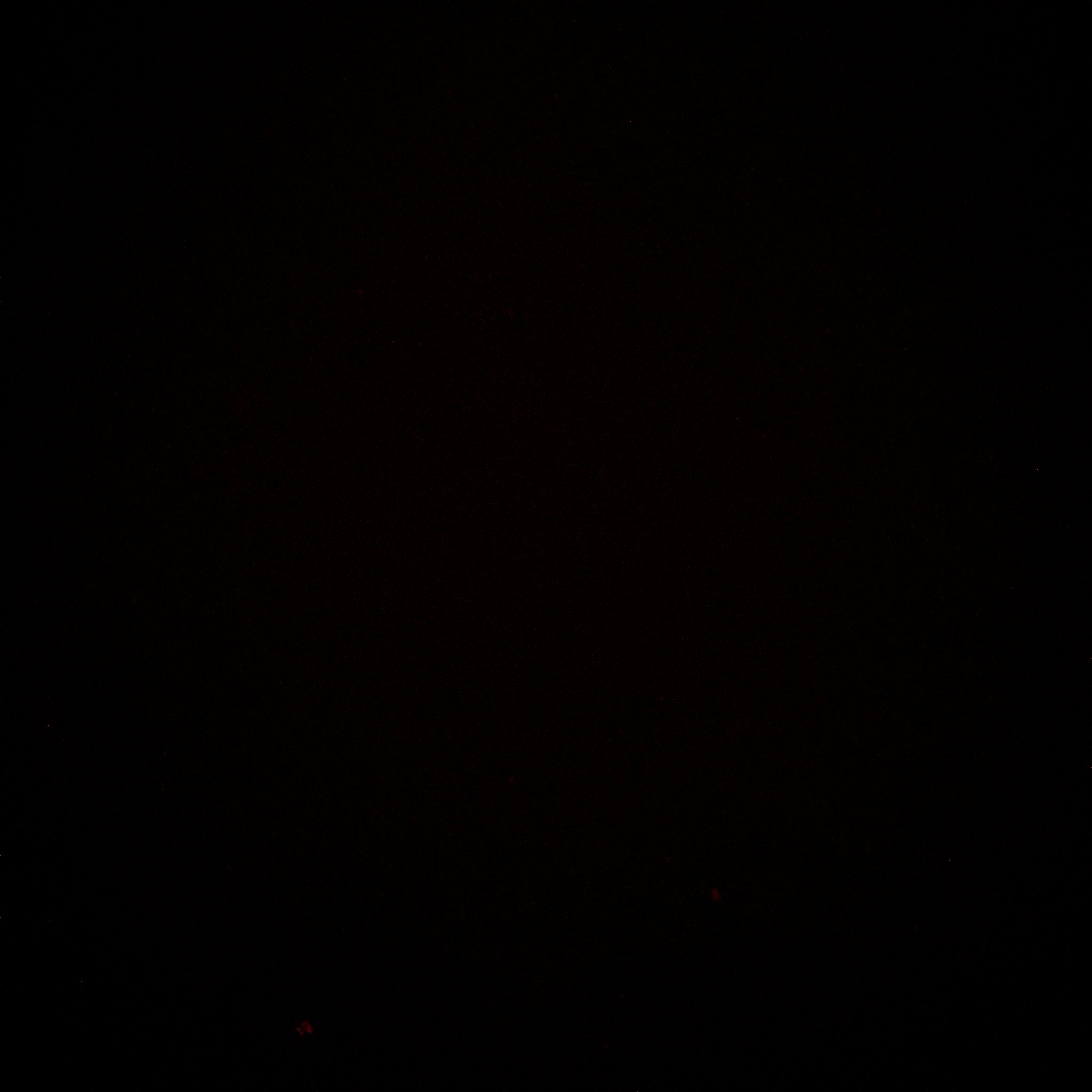

Supplement: Supplementary file 4 — Source data Fig. 3 [file 44318_2024_117_MOESM4_ESM.zip › Figure 3/3B/WT CySS_mCherry.jpg]

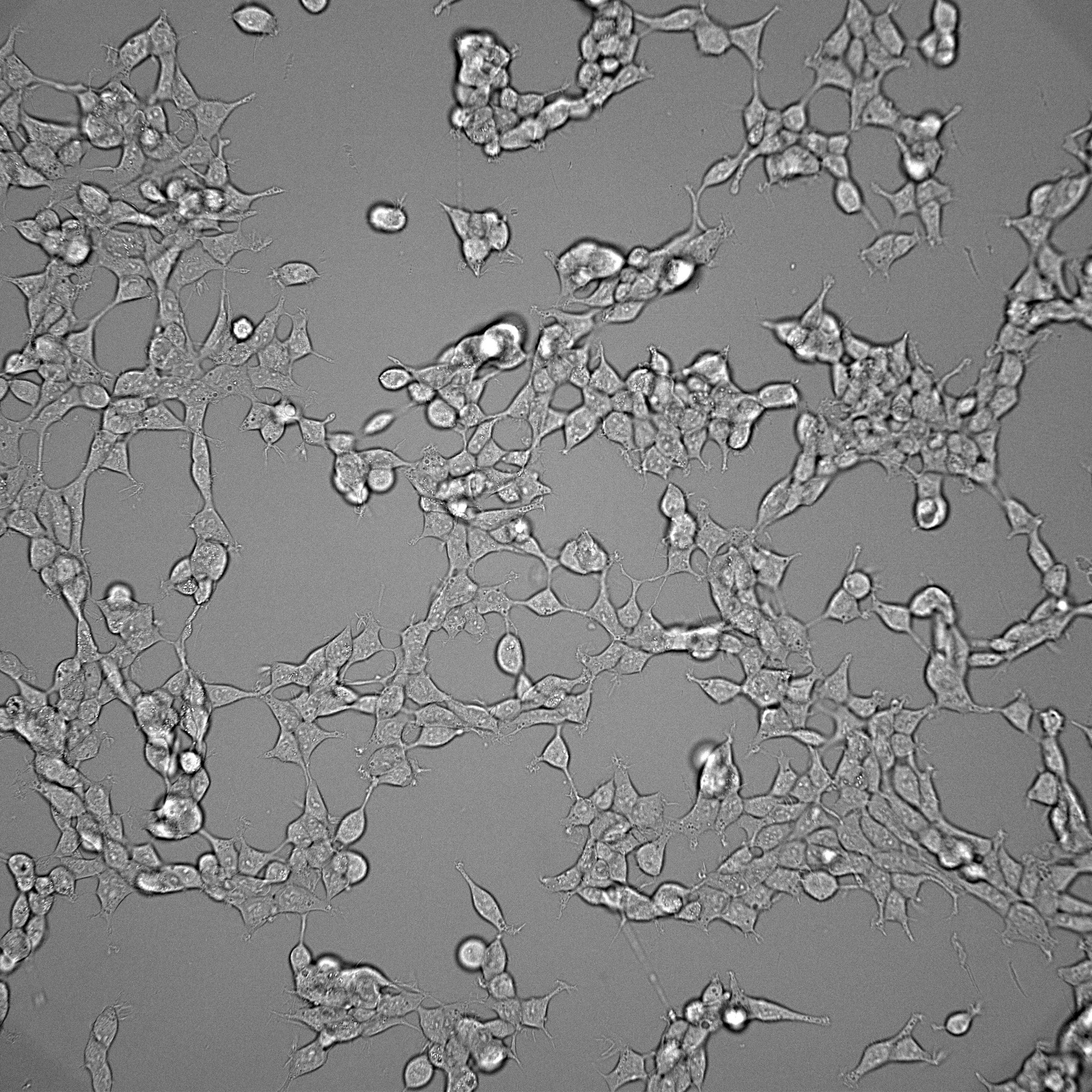

Supplement: Supplementary file 4 — Source data Fig. 3 [file 44318_2024_117_MOESM4_ESM.zip › Figure 3/3A/TRP14 KO 500 uM CySS Cell viability.jpg]

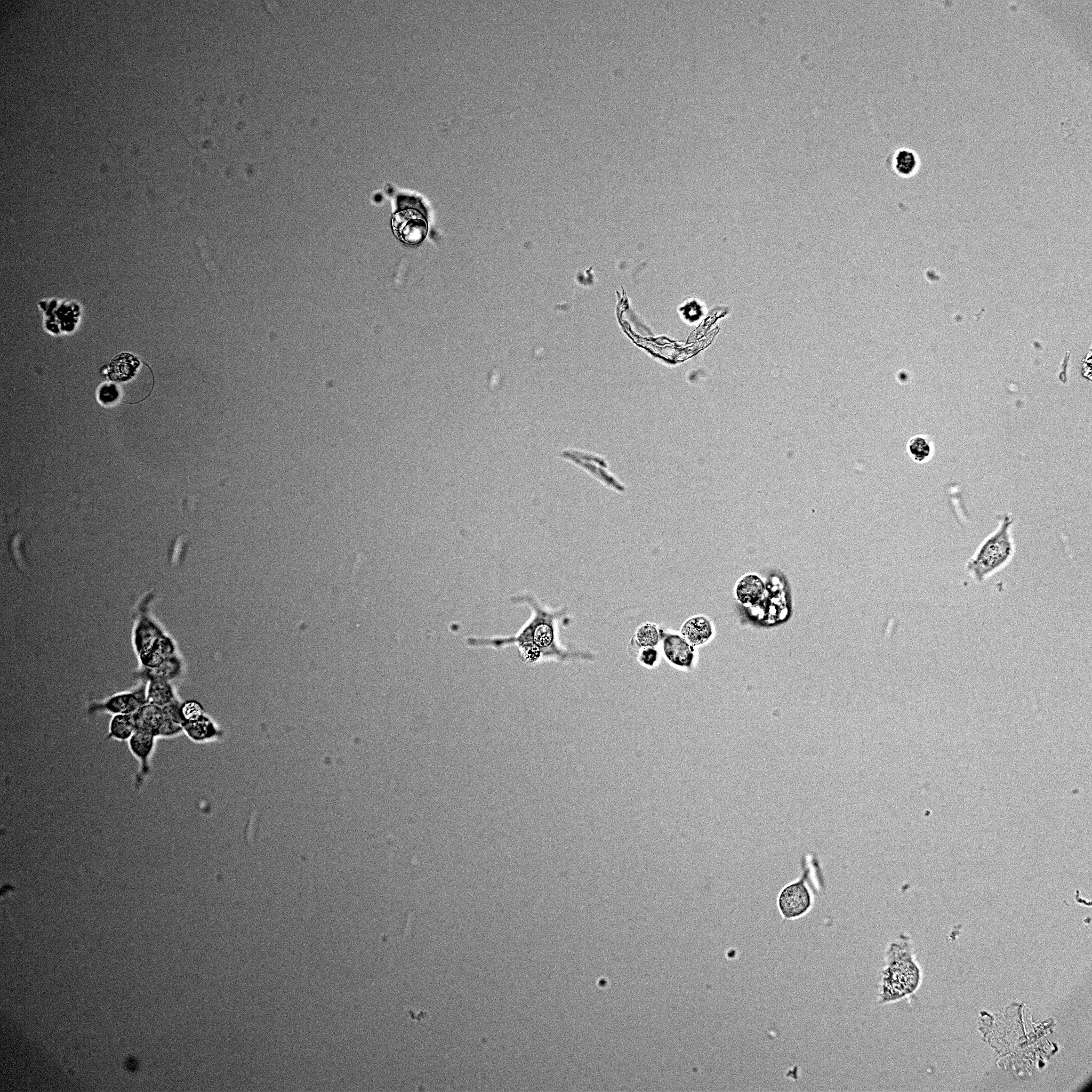

Supplement: Supplementary file 4 — Source data Fig. 3 [file 44318_2024_117_MOESM4_ESM.zip › Figure 3/3A/WT 500 uM CySS Cell viability.jpg]

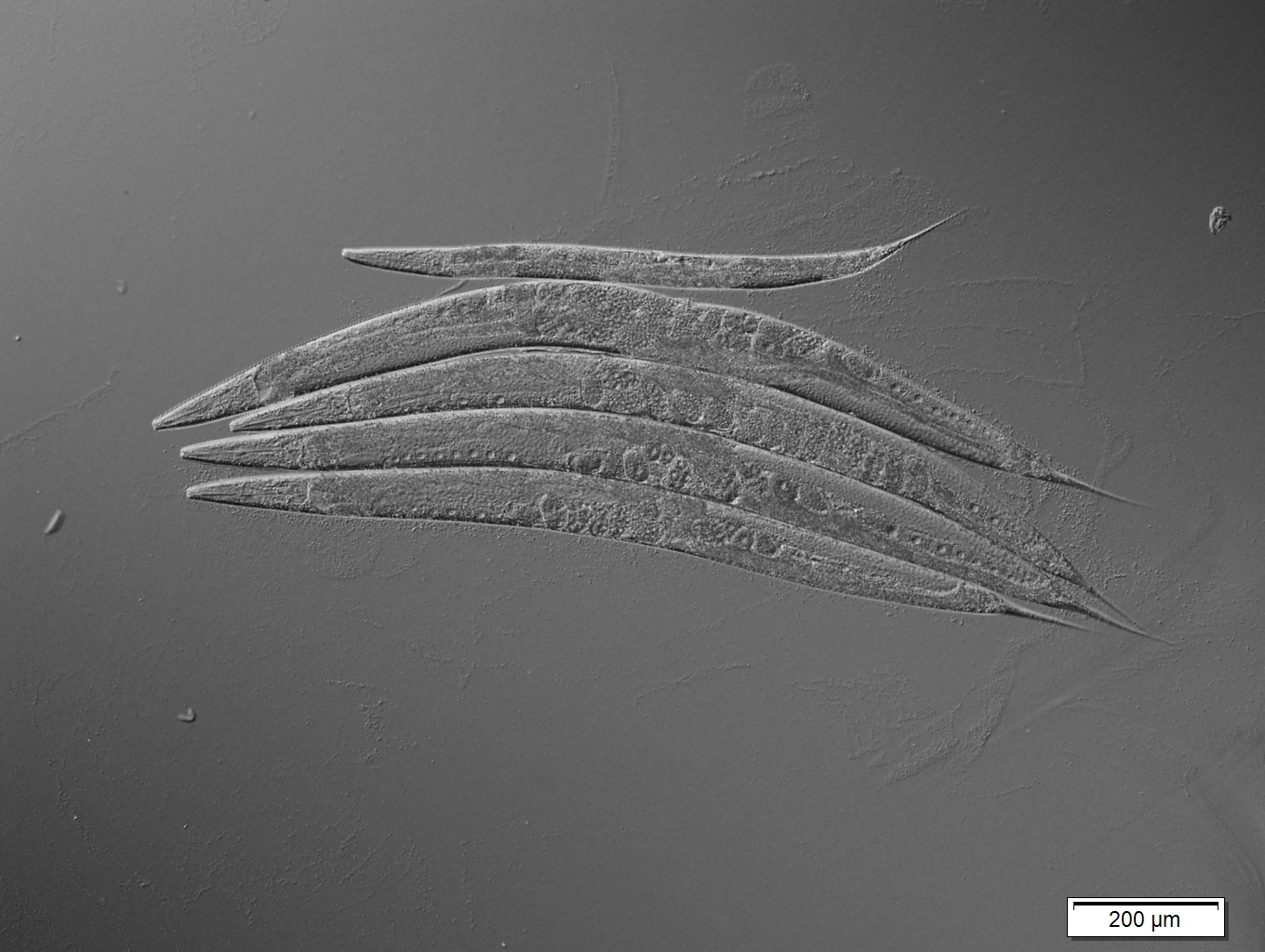

Supplement: Supplementary file 5 — Source data Fig. 4 [file 44318_2024_117_MOESM5_ESM.zip › Figure 4/4C/cbs-2_OP50.jpg]

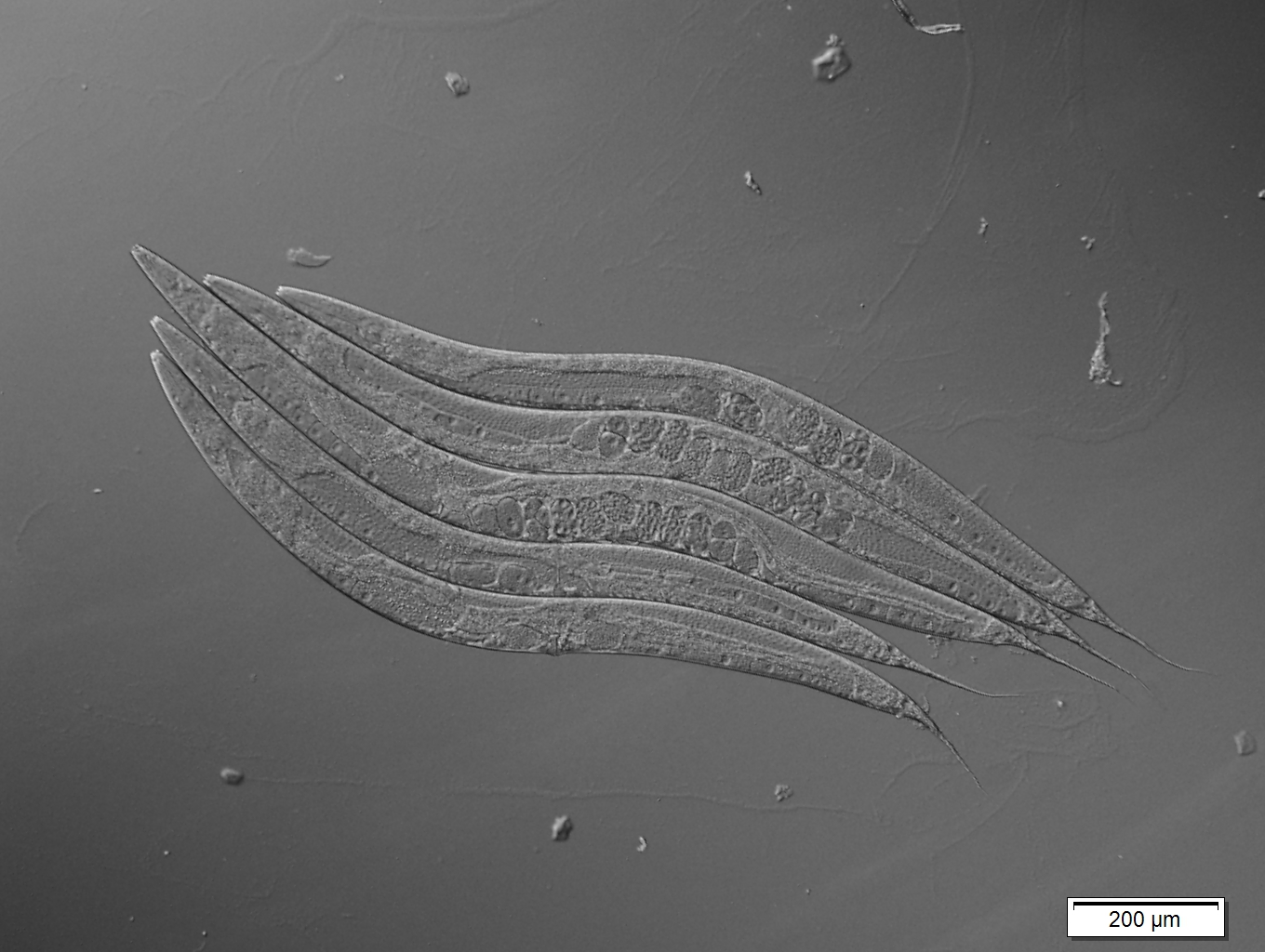

Supplement: Supplementary file 5 — Source data Fig. 4 [file 44318_2024_117_MOESM5_ESM.zip › Figure 4/4C/N2_OP50.jpg]

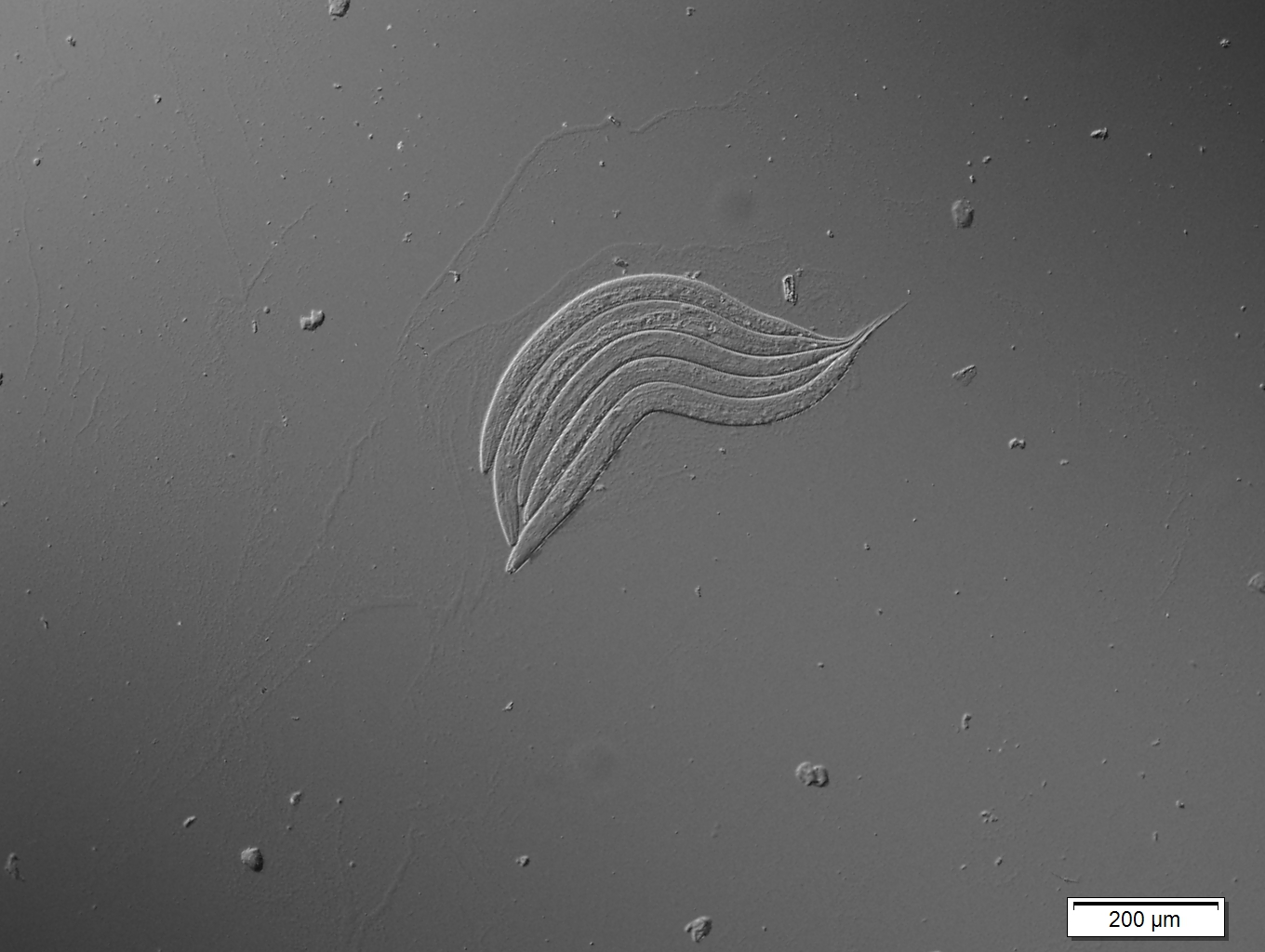

Supplement: Supplementary file 5 — Source data Fig. 4 [file 44318_2024_117_MOESM5_ESM.zip › Figure 4/4C/cbs-2cbs-1_OP50.jpg]

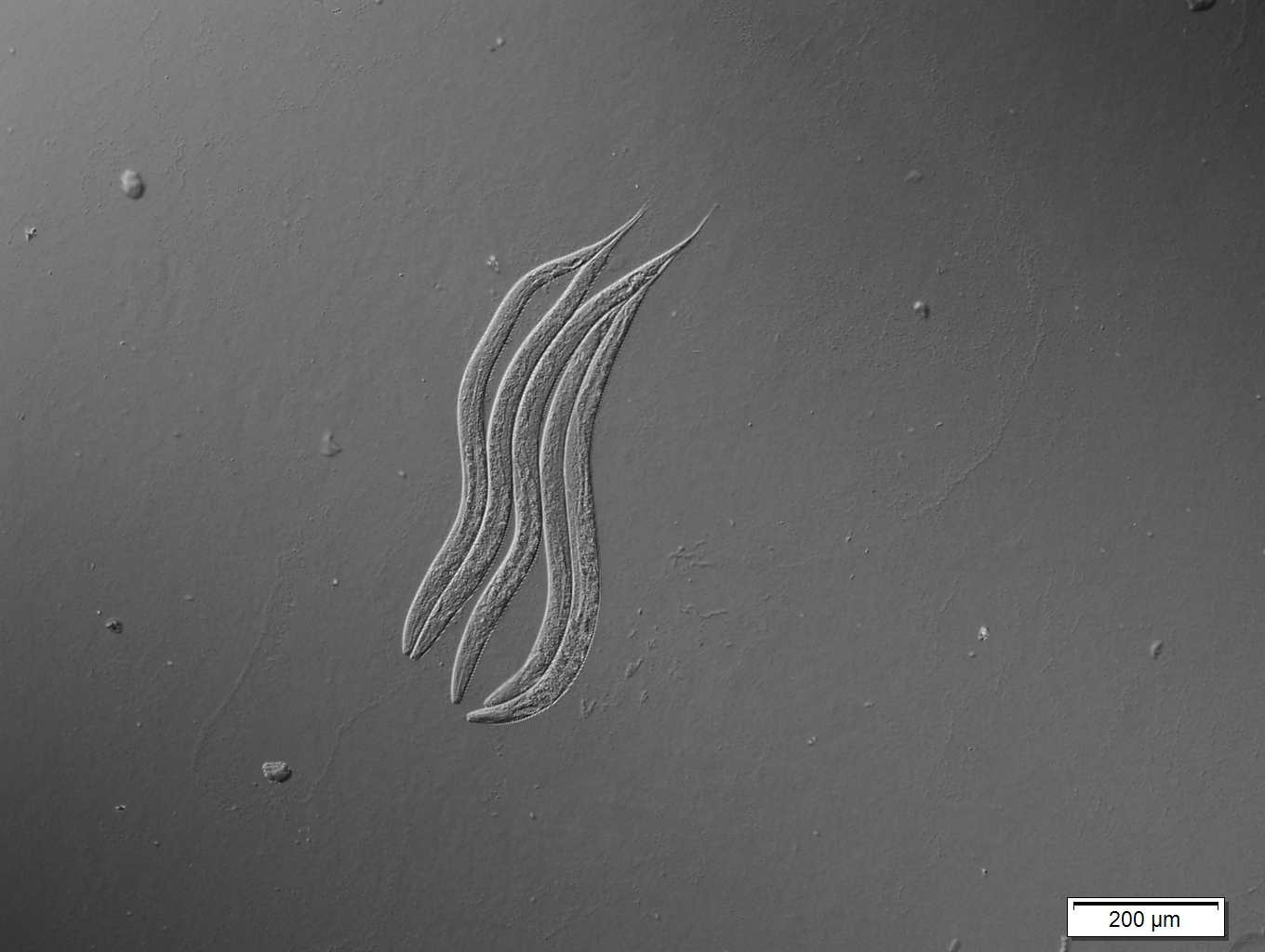

Supplement: Supplementary file 5 — Source data Fig. 4 [file 44318_2024_117_MOESM5_ESM.zip › Figure 4/4C/cbs-1_OP50.jpg]

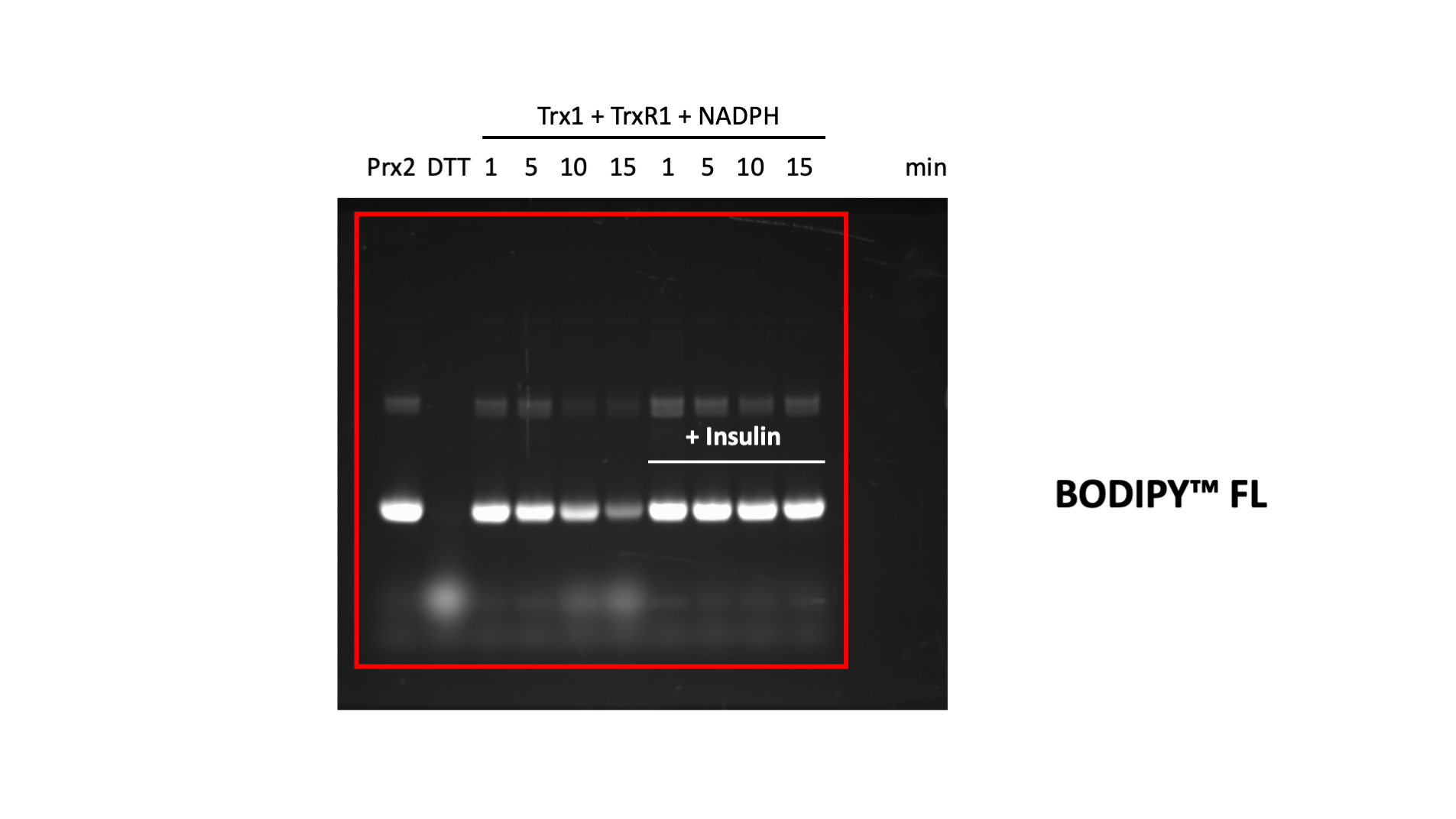

Supplement: Supplementary file 6 — Source data Fig. 5 [file 44318_2024_117_MOESM6_ESM.zip › Figure 5/5G/BODIPY FL image from the gel - Trx1.tiff]

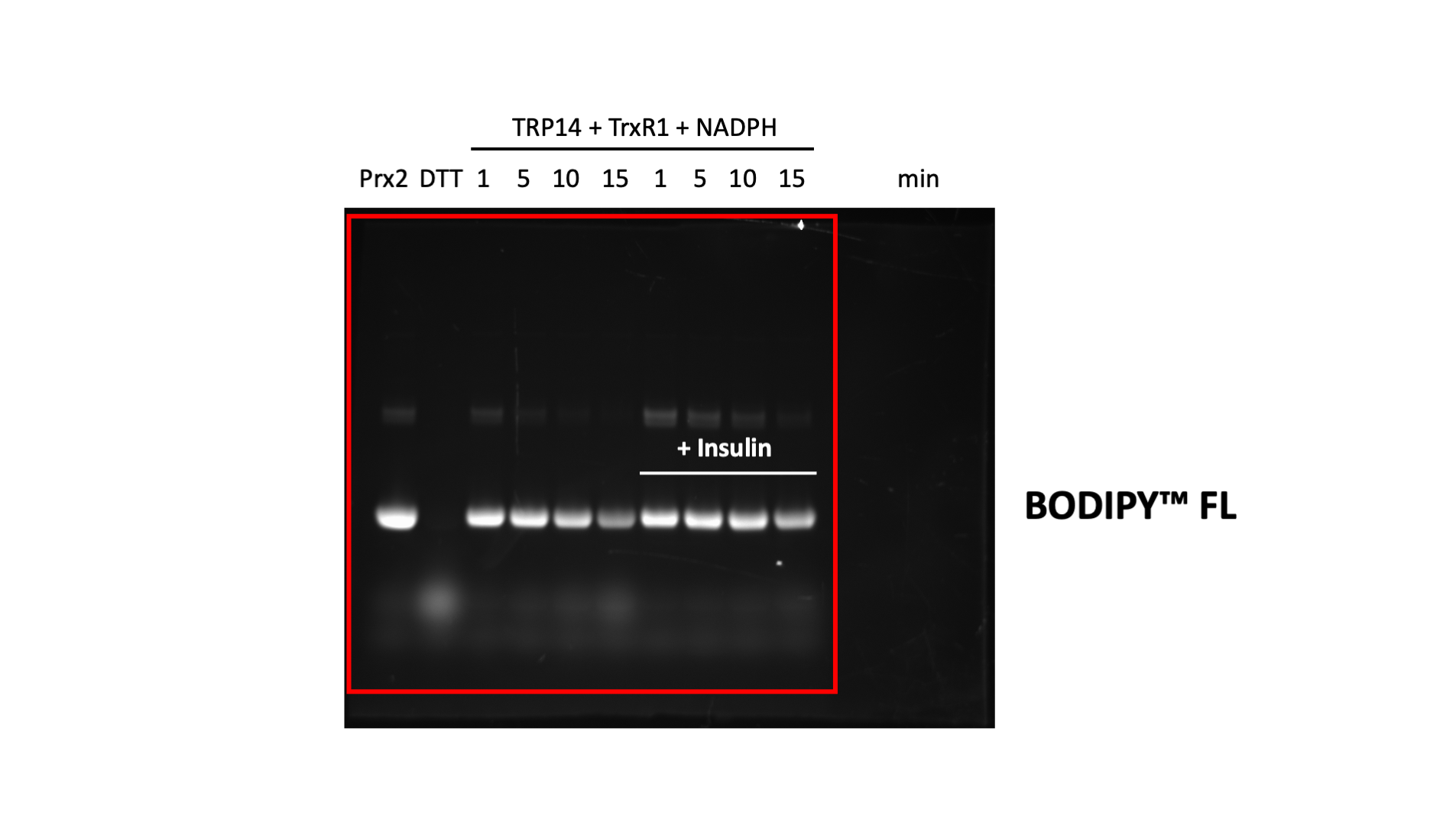

Supplement: Supplementary file 6 — Source data Fig. 5 [file 44318_2024_117_MOESM6_ESM.zip › Figure 5/5G/BODIPY FL image from the gel - TRP14.tiff]

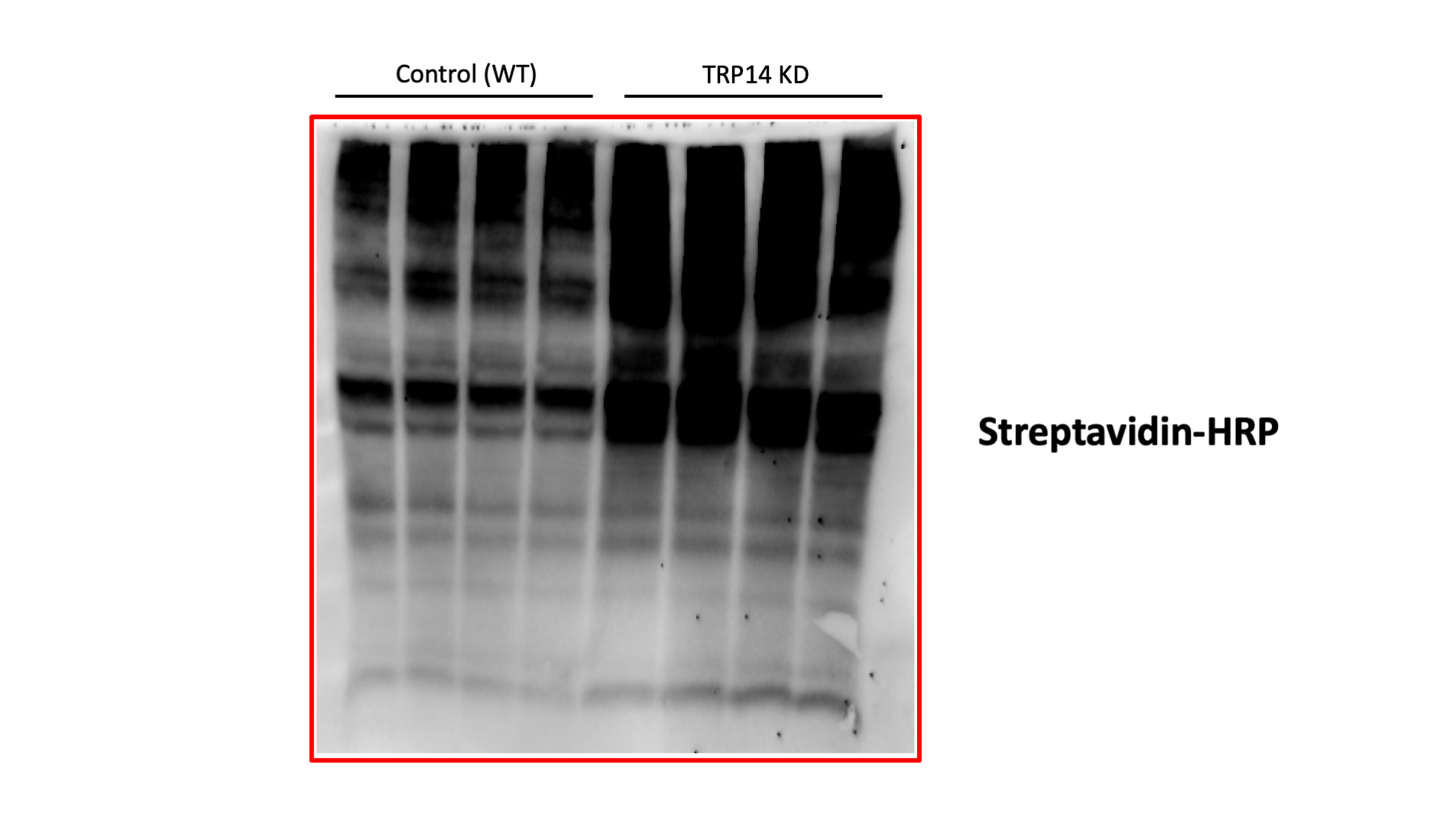

Supplement: Supplementary file 6 — Source data Fig. 5 [file 44318_2024_117_MOESM6_ESM.zip › Figure 5/5A/Western blot - Streptavidin.tiff]

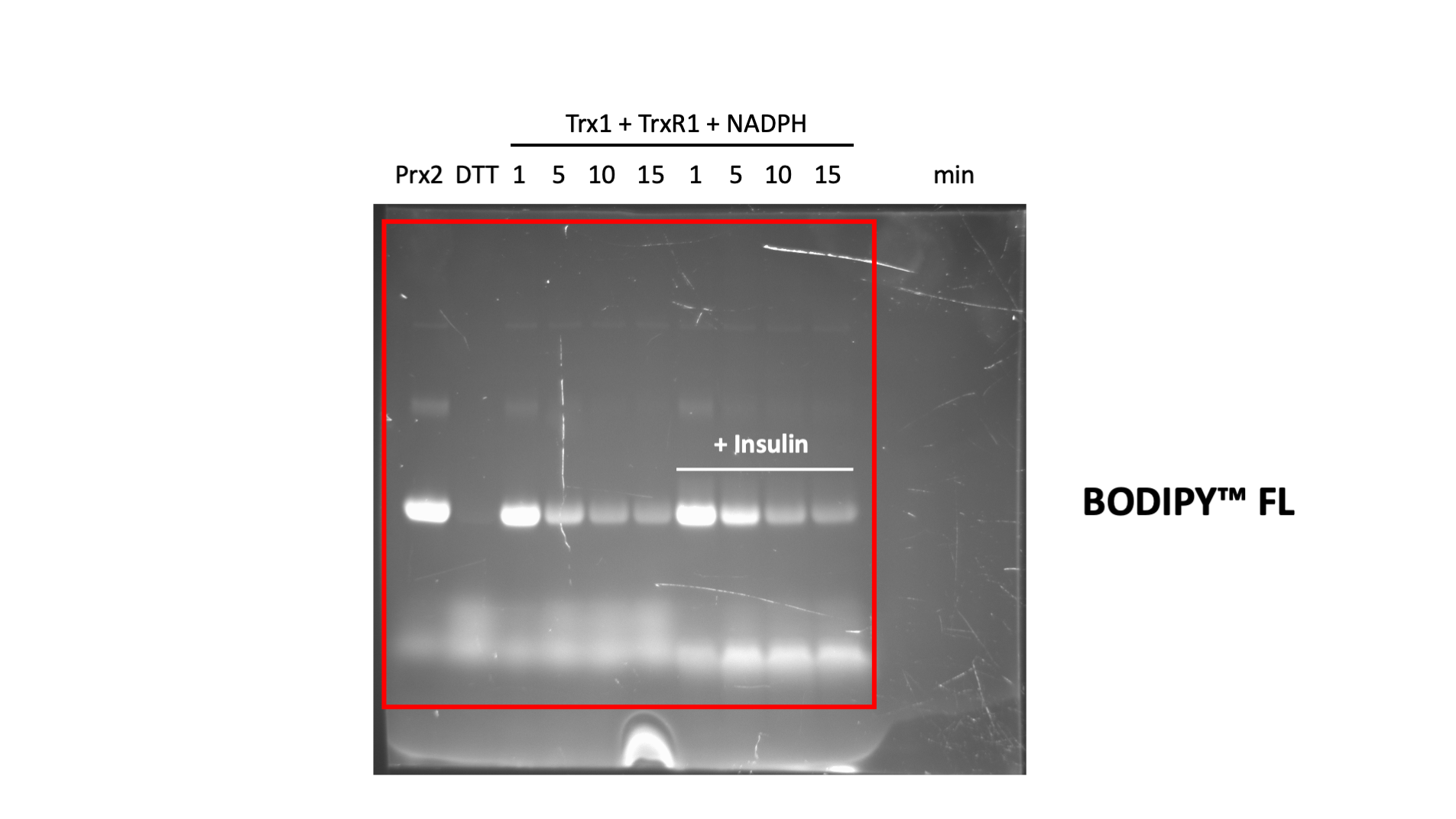

Supplement: Supplementary file 6 — Source data Fig. 5 [file 44318_2024_117_MOESM6_ESM.zip › Figure 5/5F/BOSIPY FL image from the gel - Trx1.tiff]

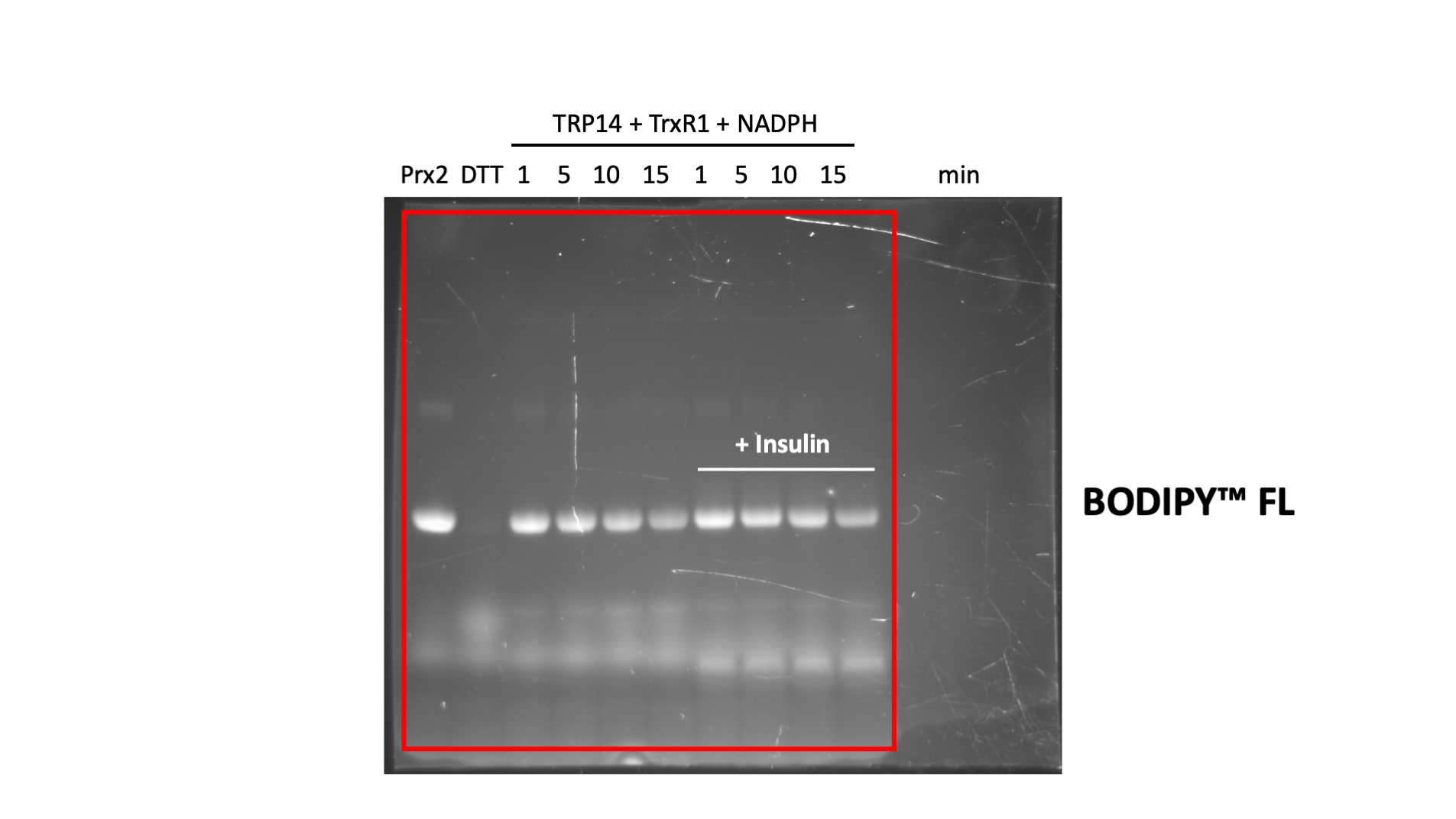

Supplement: Supplementary file 6 — Source data Fig. 5 [file 44318_2024_117_MOESM6_ESM.zip › Figure 5/5F/BODIPY FL image from the gel - TRP14.tiff]

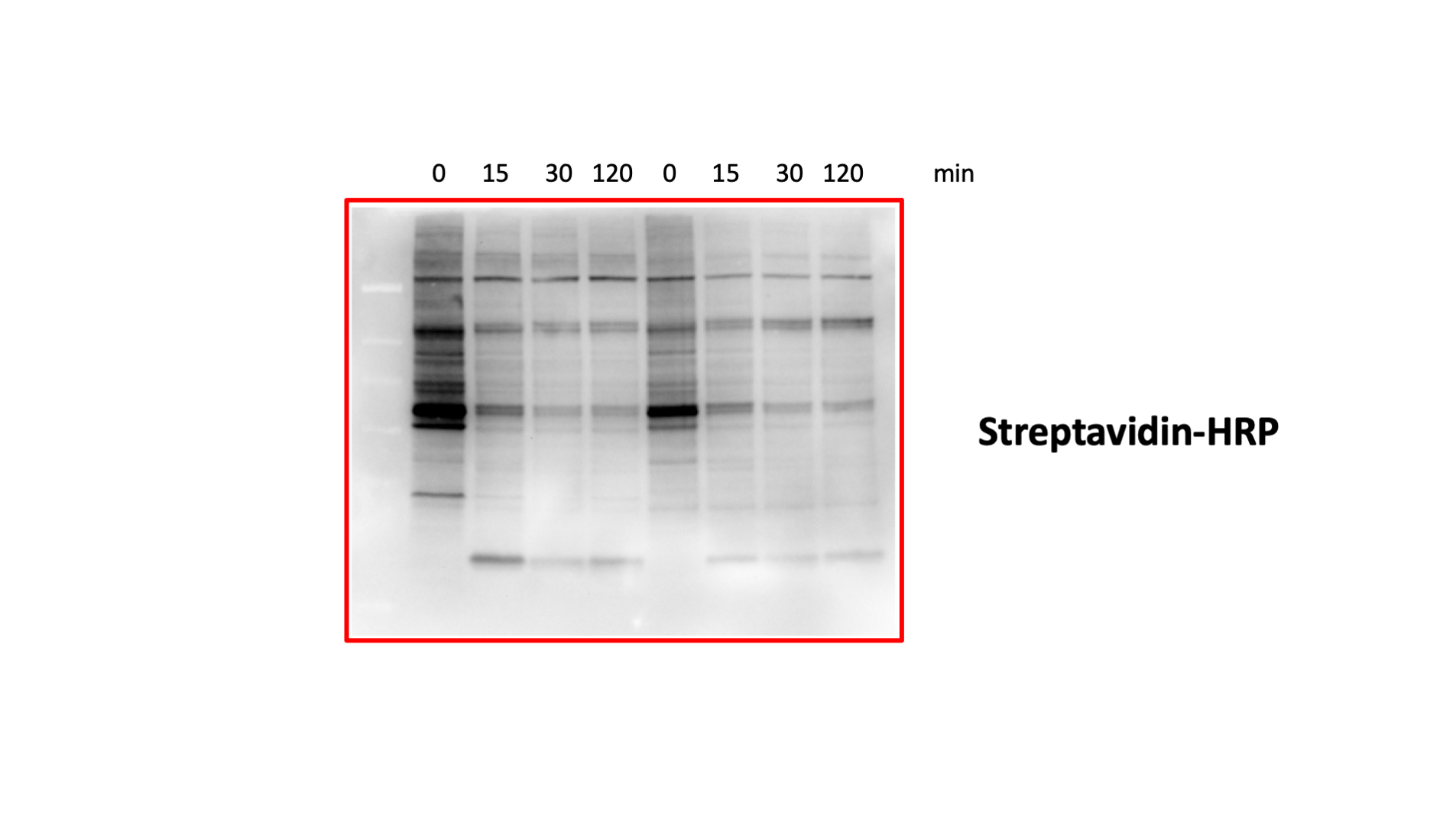

Supplement: Supplementary file 6 — Source data Fig. 5 [file 44318_2024_117_MOESM6_ESM.zip › Figure 5/5B/Western blot - Streptavidin.tiff]

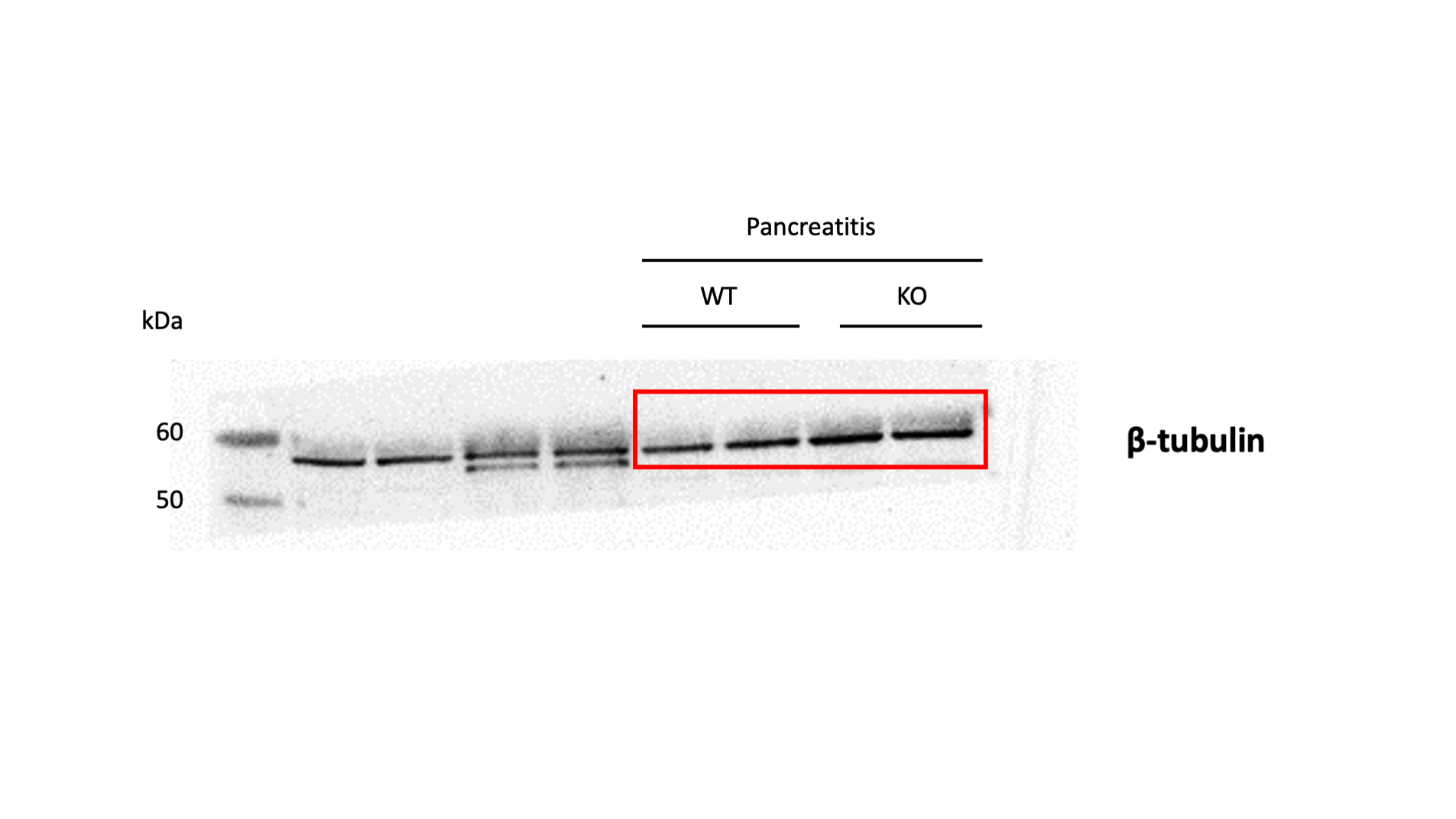

Supplement: Supplementary file 8 — Source data Fig. 8 [file 44318_2024_117_MOESM8_ESM.zip › Figure 8/8F/Western blot Pancreatitis - Tubulin.tiff]

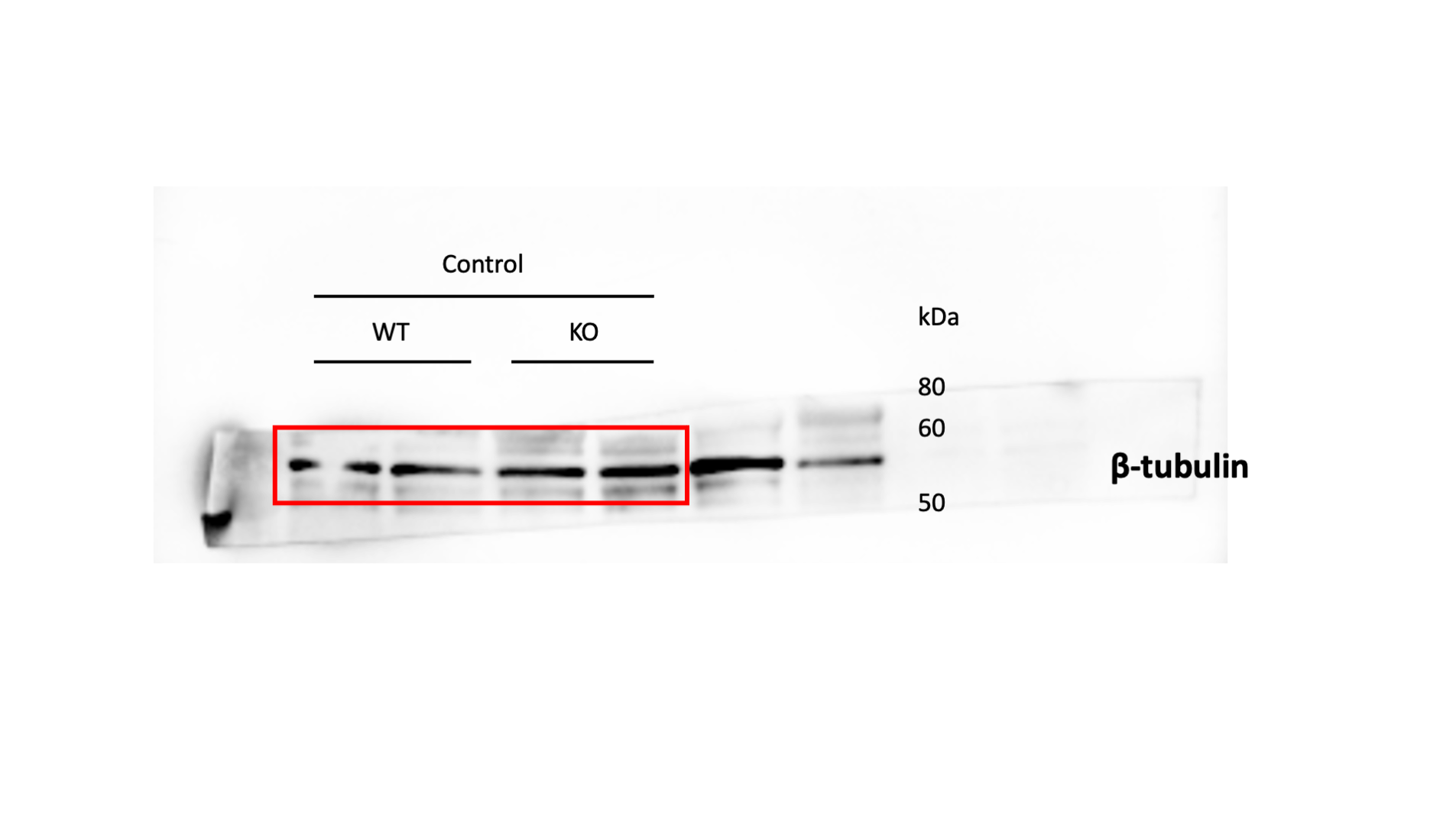

Supplement: Supplementary file 8 — Source data Fig. 8 [file 44318_2024_117_MOESM8_ESM.zip › Figure 8/8F/Western blot Control - Tubulin.tiff]

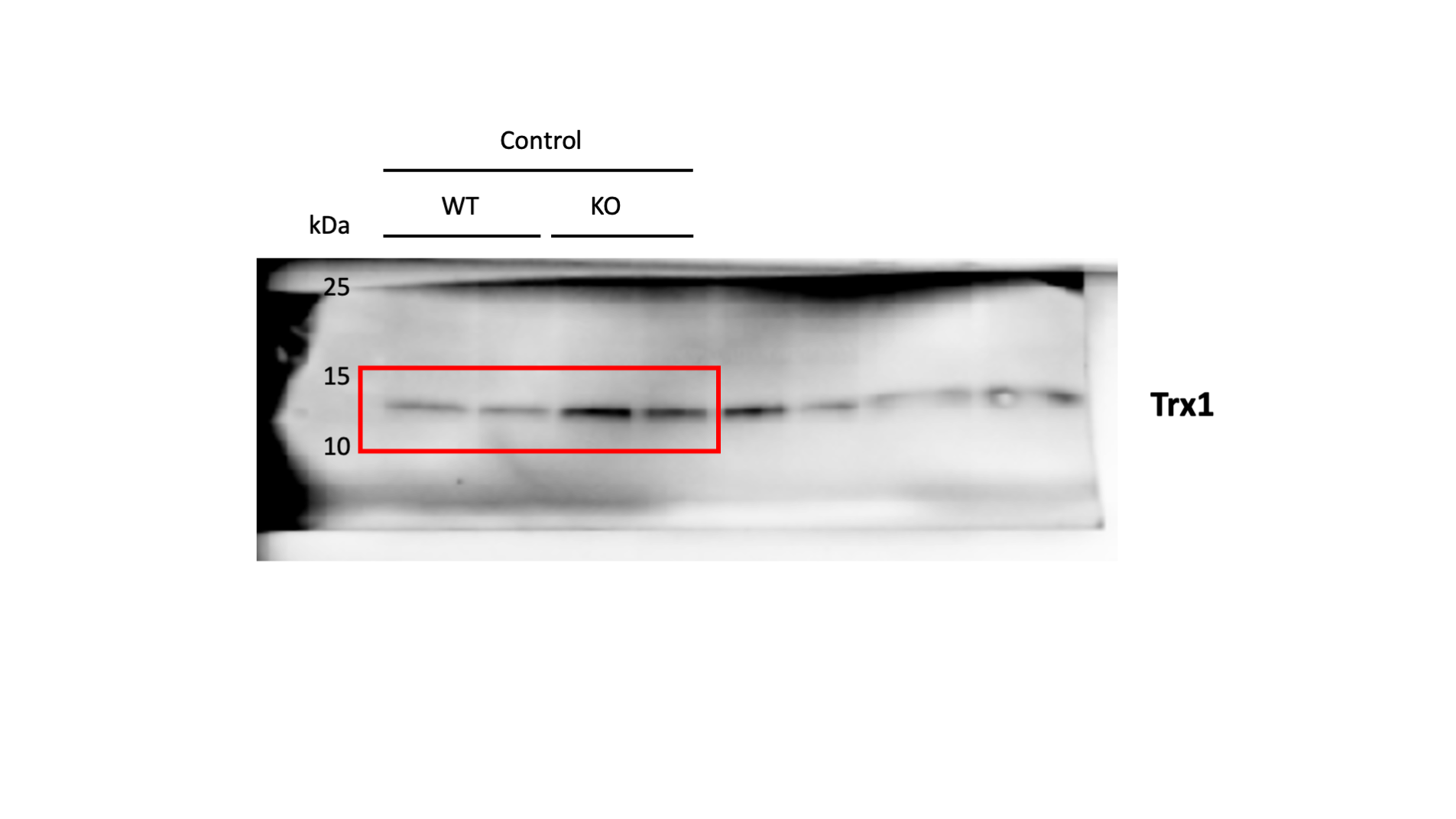

Supplement: Supplementary file 8 — Source data Fig. 8 [file 44318_2024_117_MOESM8_ESM.zip › Figure 8/8F/Western blot Control - Trx1.tiff]

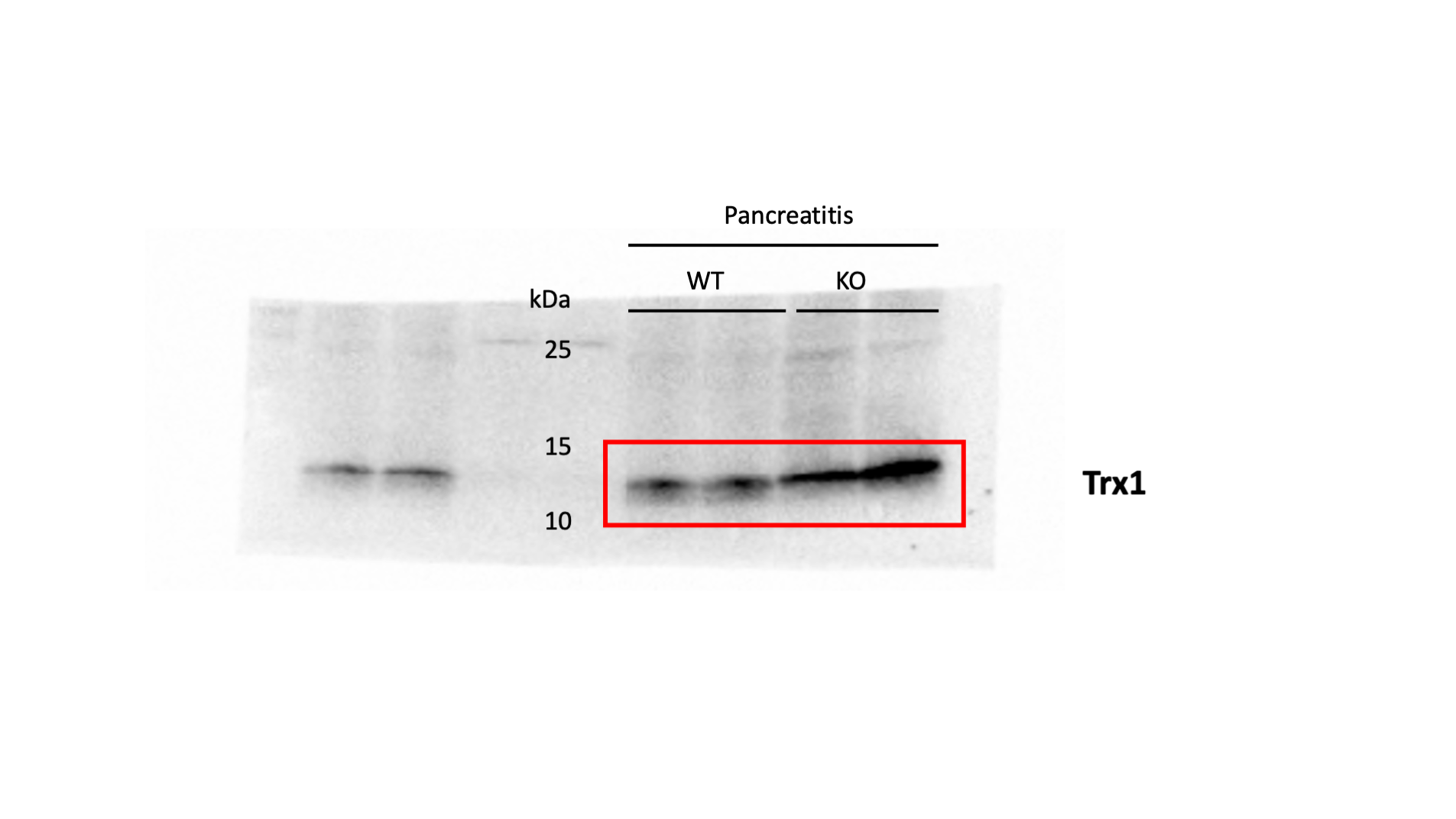

Supplement: Supplementary file 8 — Source data Fig. 8 [file 44318_2024_117_MOESM8_ESM.zip › Figure 8/8F/Western blot Pancreatitis - Trx1.tiff]

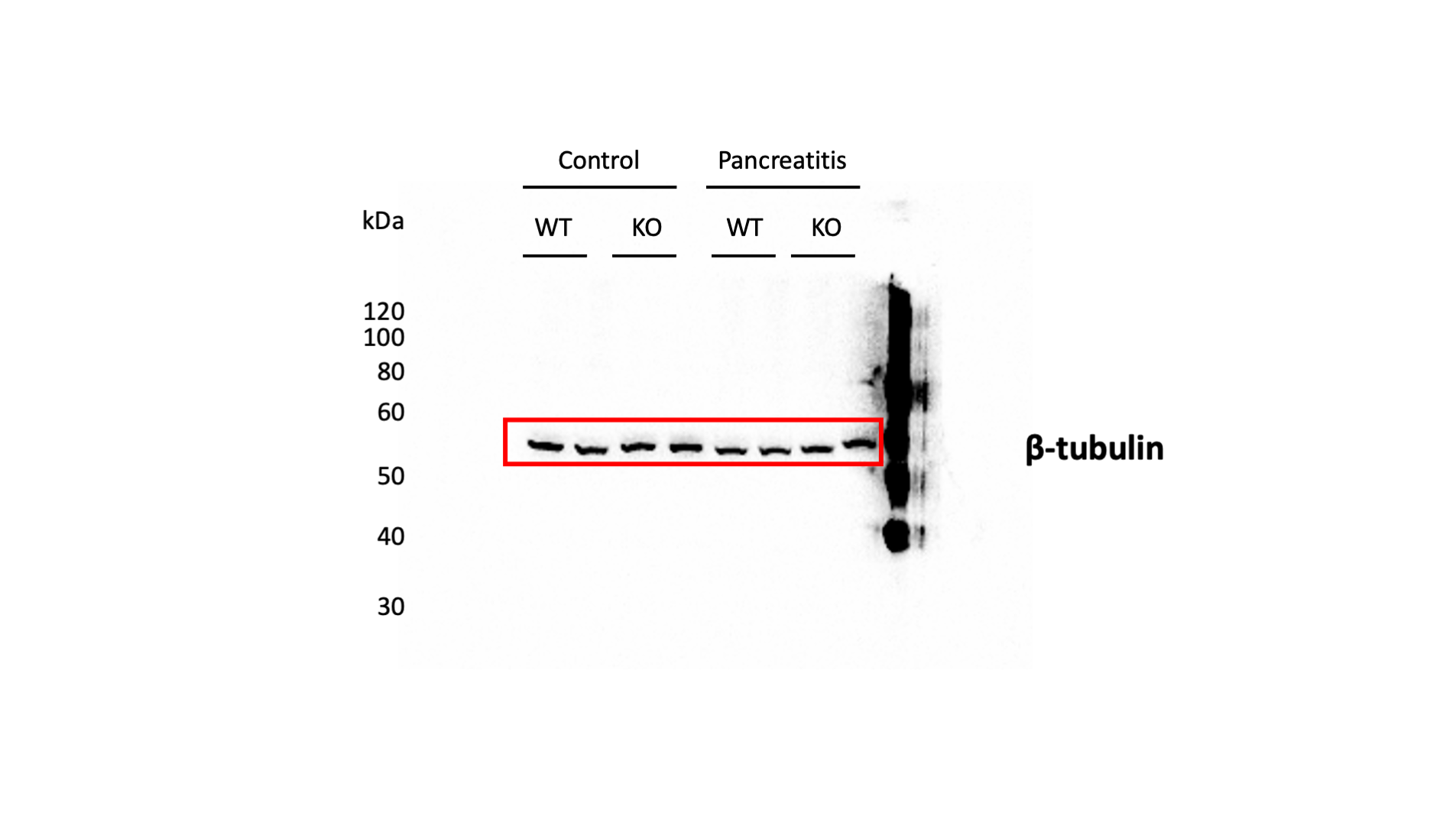

Supplement: Supplementary file 8 — Source data Fig. 8 [file 44318_2024_117_MOESM8_ESM.zip › Figure 8/8D/Western blot Tubulin.tiff]

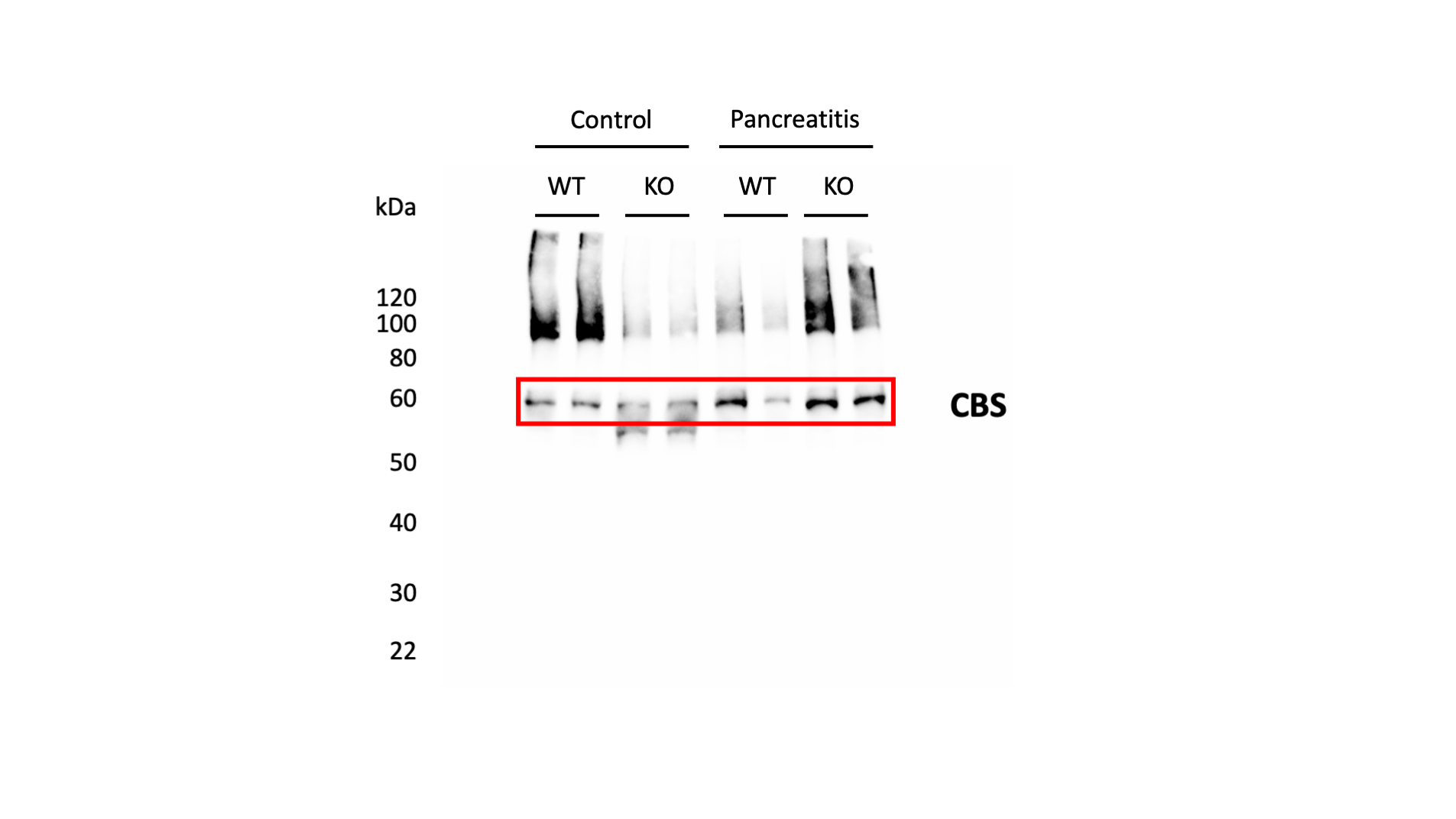

Supplement: Supplementary file 8 — Source data Fig. 8 [file 44318_2024_117_MOESM8_ESM.zip › Figure 8/8D/Western blot CBS.tiff]

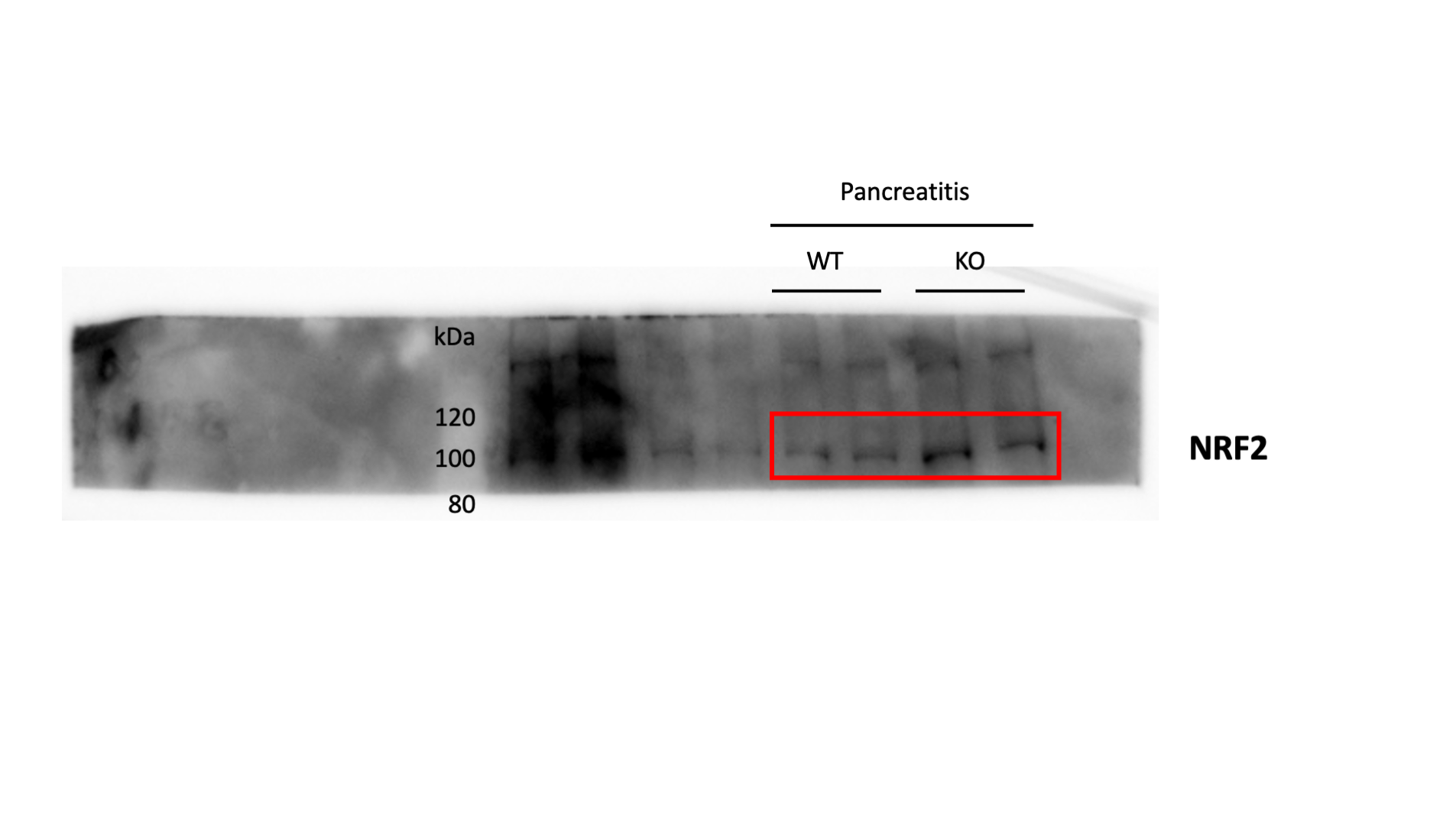

Supplement: Supplementary file 8 — Source data Fig. 8 [file 44318_2024_117_MOESM8_ESM.zip › Figure 8/8L/Western blot NRF2.tiff]

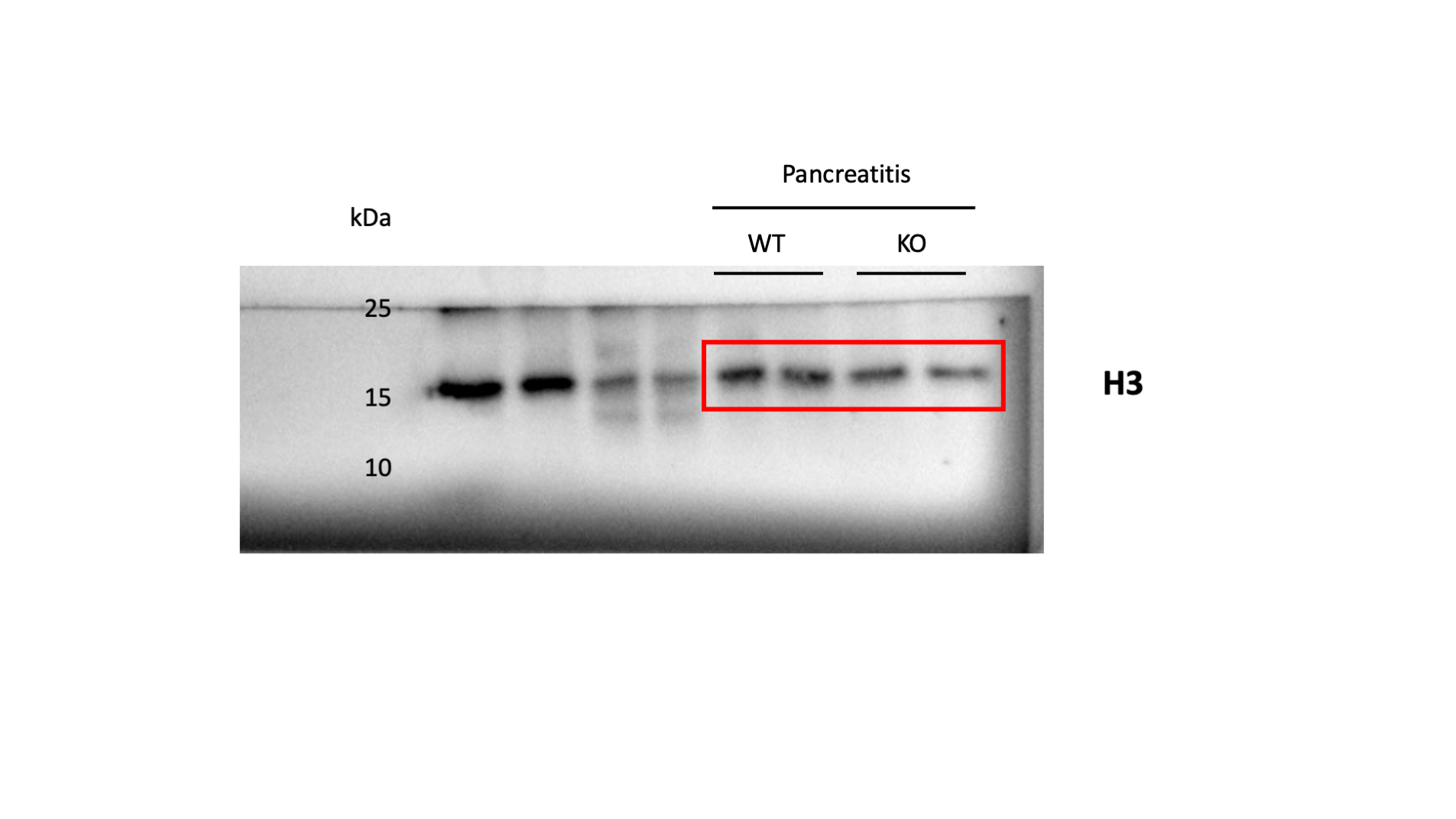

Supplement: Supplementary file 8 — Source data Fig. 8 [file 44318_2024_117_MOESM8_ESM.zip › Figure 8/8L/Western blot H3.tiff]

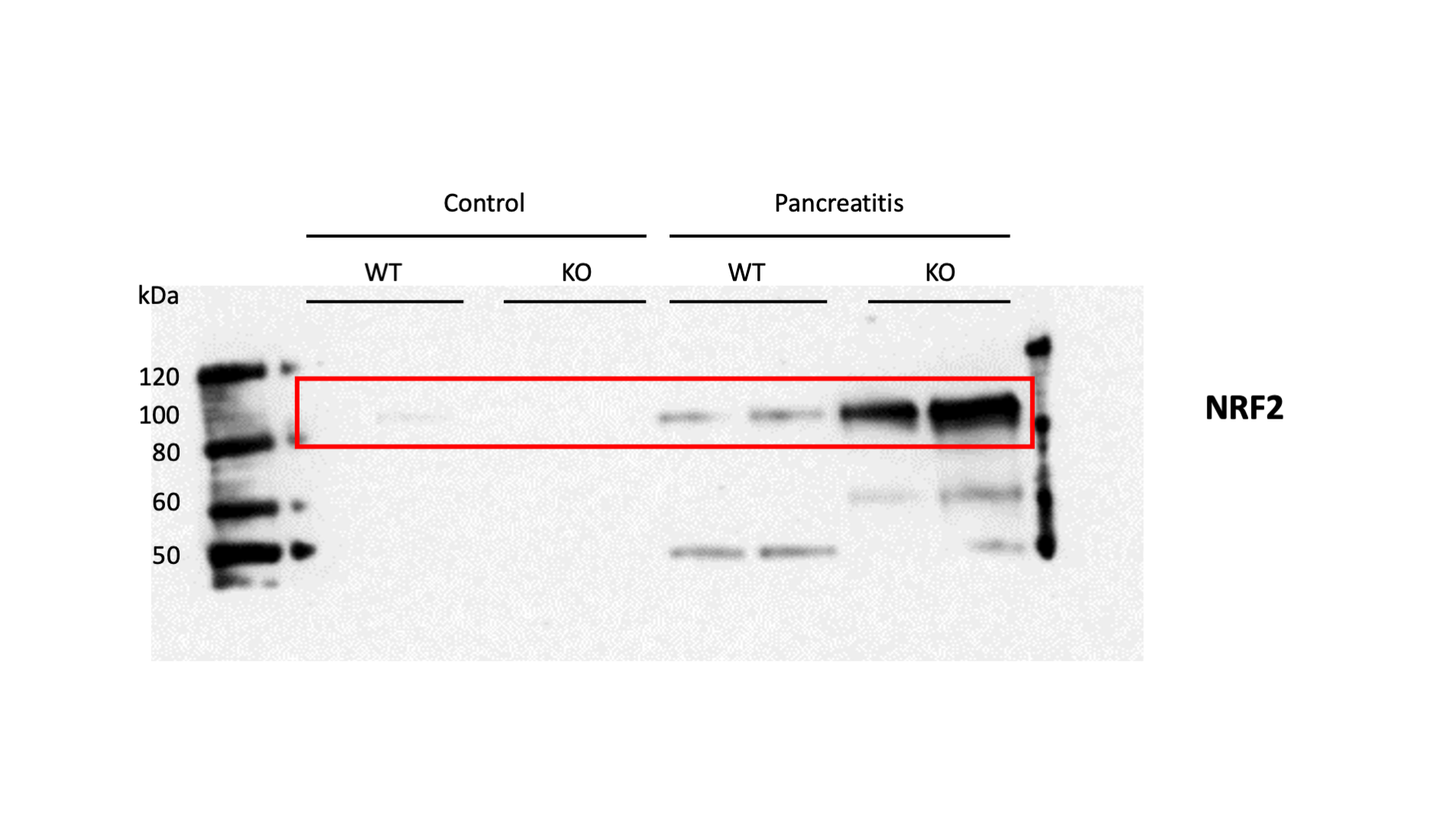

Supplement: Supplementary file 8 — Source data Fig. 8 [file 44318_2024_117_MOESM8_ESM.zip › Figure 8/8K/Western blot NRF2.tiff]

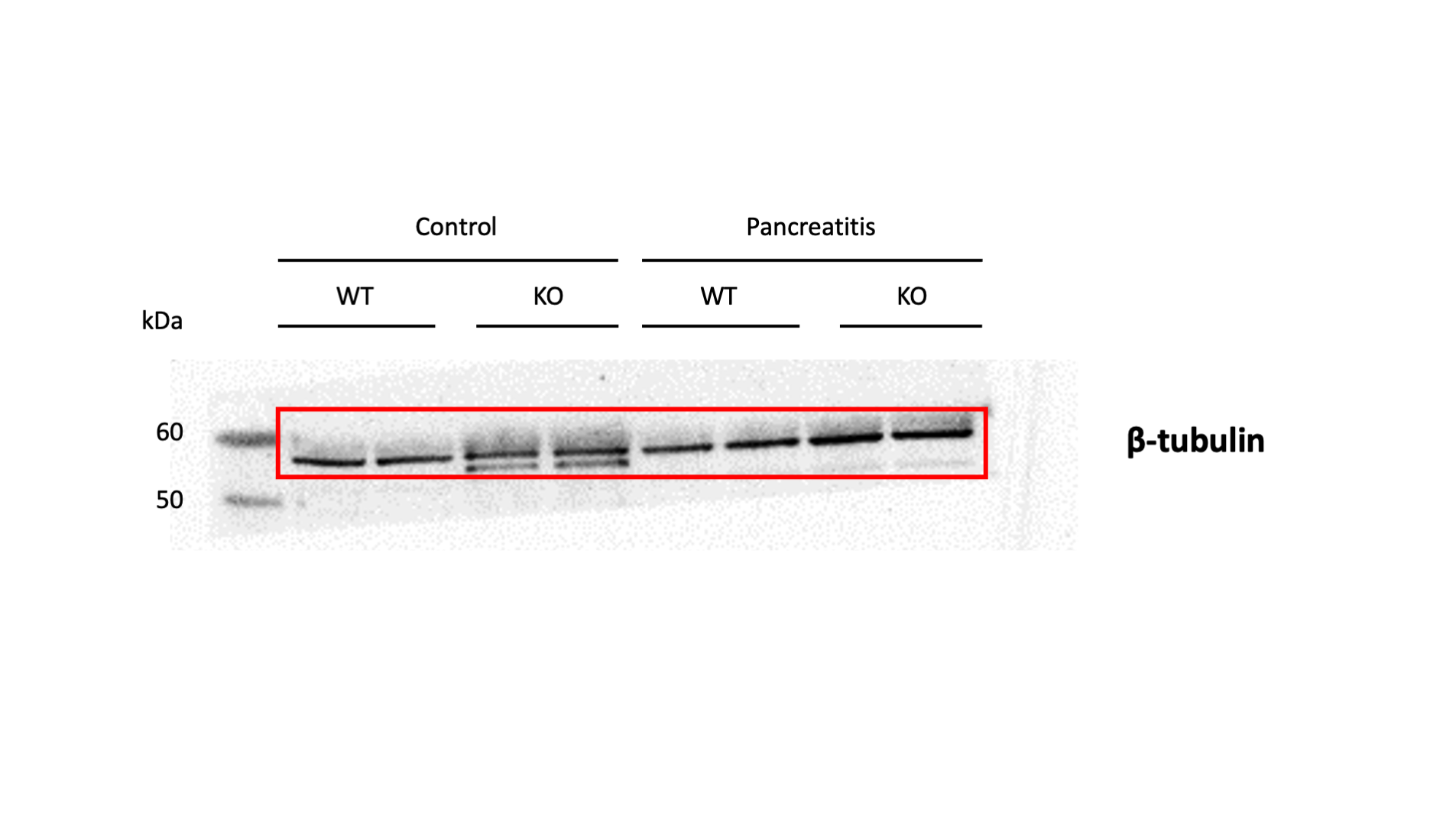

Supplement: Supplementary file 8 — Source data Fig. 8 [file 44318_2024_117_MOESM8_ESM.zip › Figure 8/8K/Western blot Tubulin.tiff]

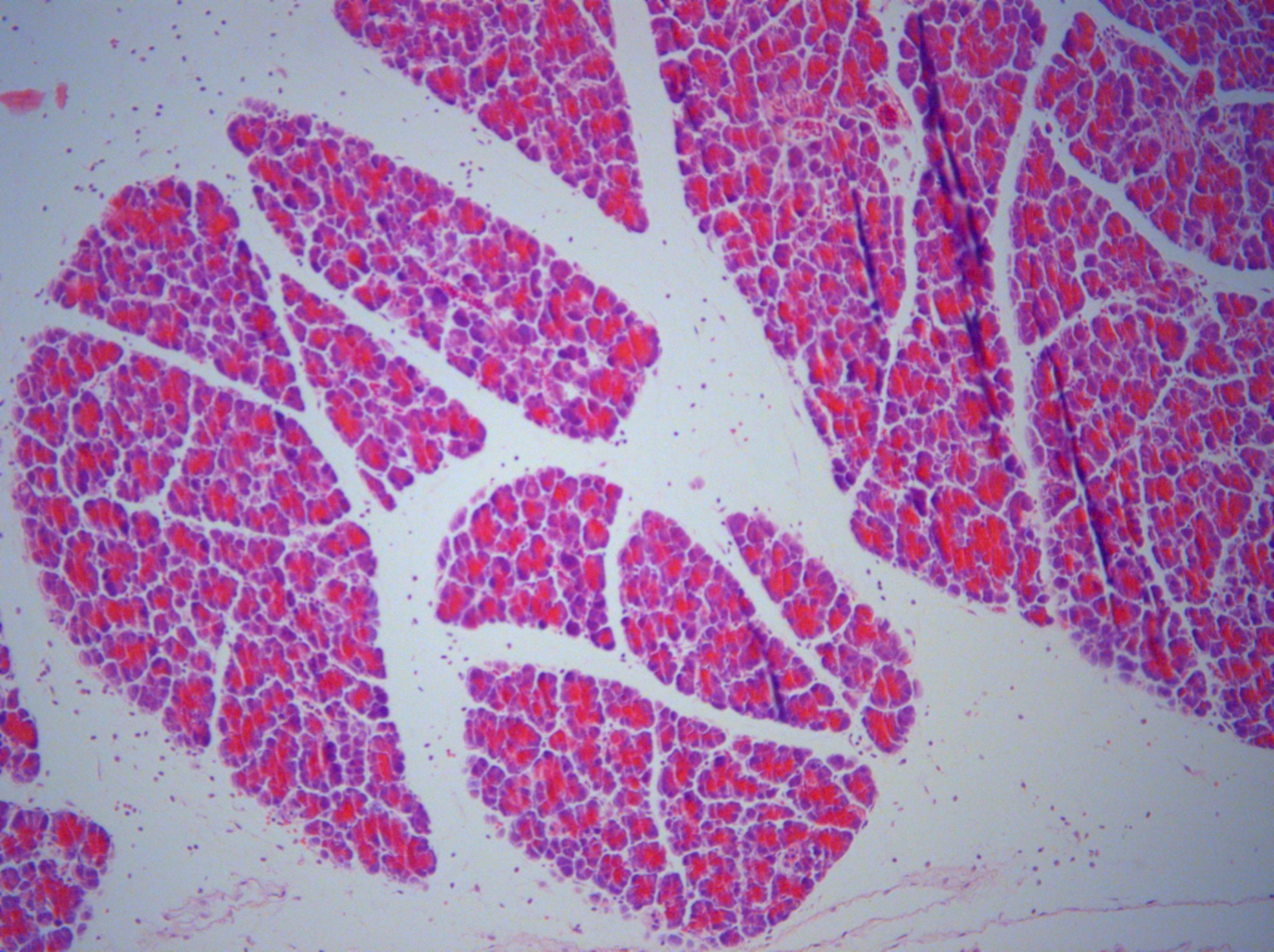

Supplement: Supplementary file 9 — Source data Fig. 9 [file 44318_2024_117_MOESM9_ESM.zip › Figure 9/9E/WT Cerulein.JPG]

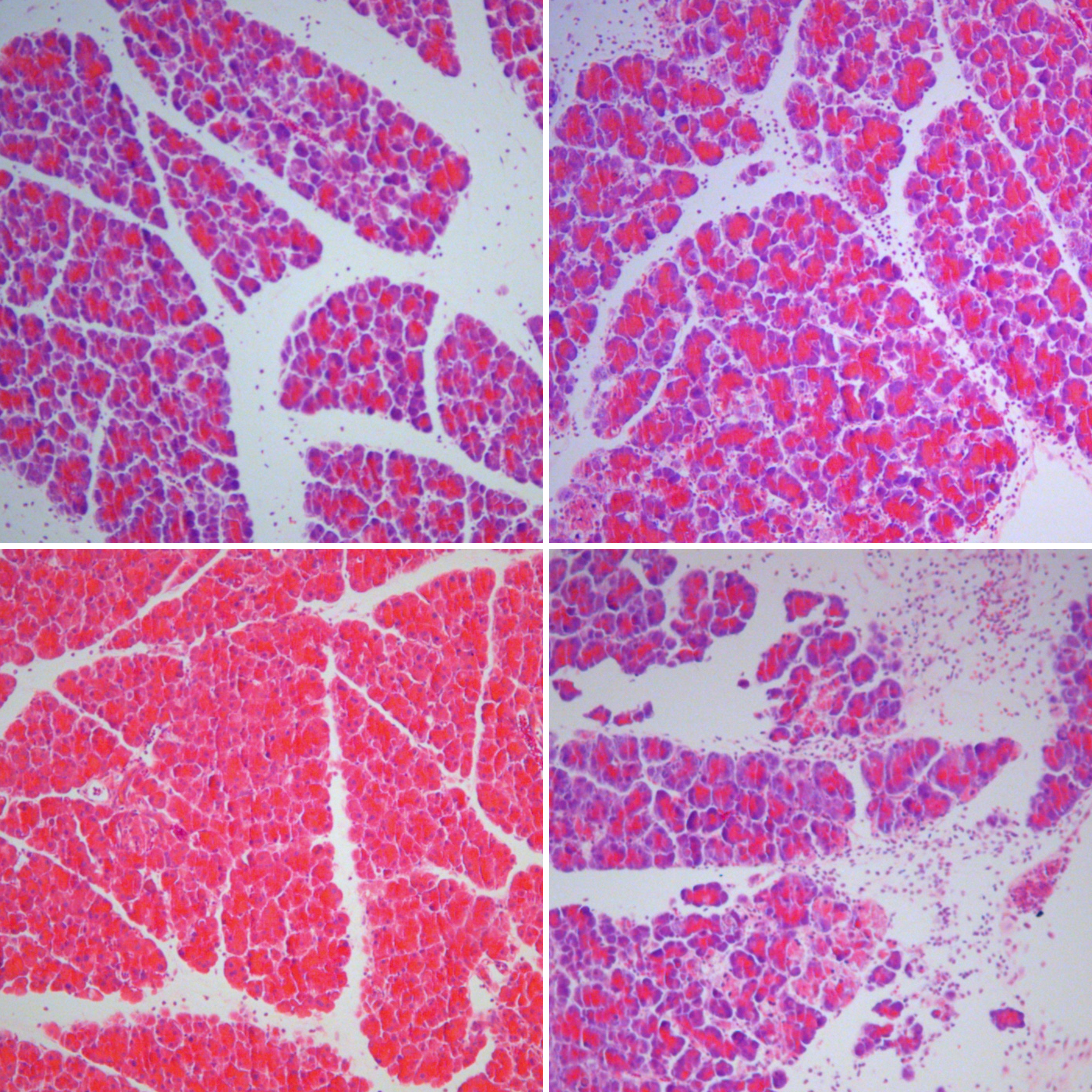

Supplement: Supplementary file 9 — Source data Fig. 9 [file 44318_2024_117_MOESM9_ESM.zip › Figure 9/9E/Figure 9E - H&E Pancreatitis + AOAA.tif]

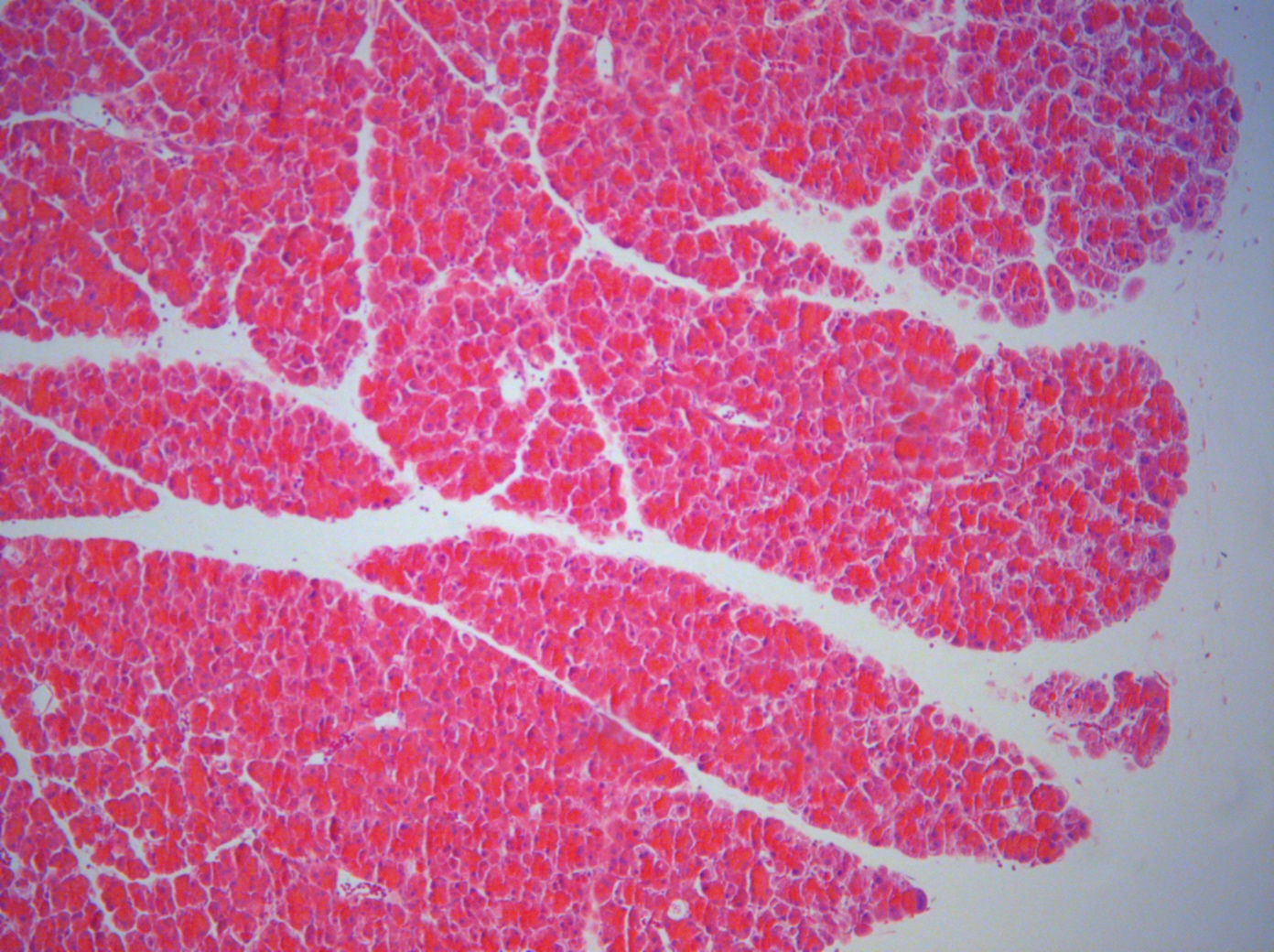

Supplement: Supplementary file 9 — Source data Fig. 9 [file 44318_2024_117_MOESM9_ESM.zip › Figure 9/9E/KO Cerulein.JPG]

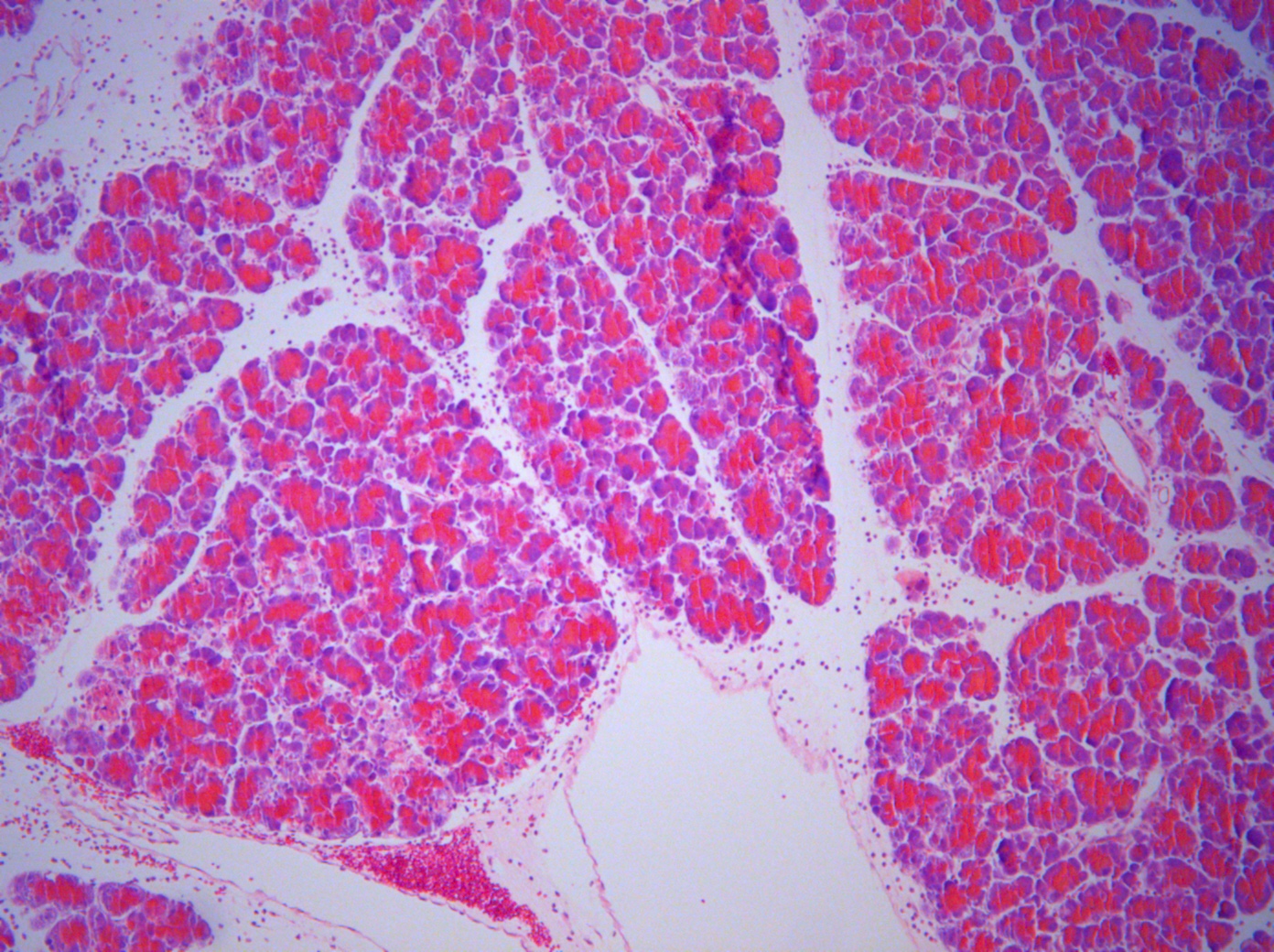

Supplement: Supplementary file 9 — Source data Fig. 9 [file 44318_2024_117_MOESM9_ESM.zip › Figure 9/9E/WT Cerulein AOAA.JPG]

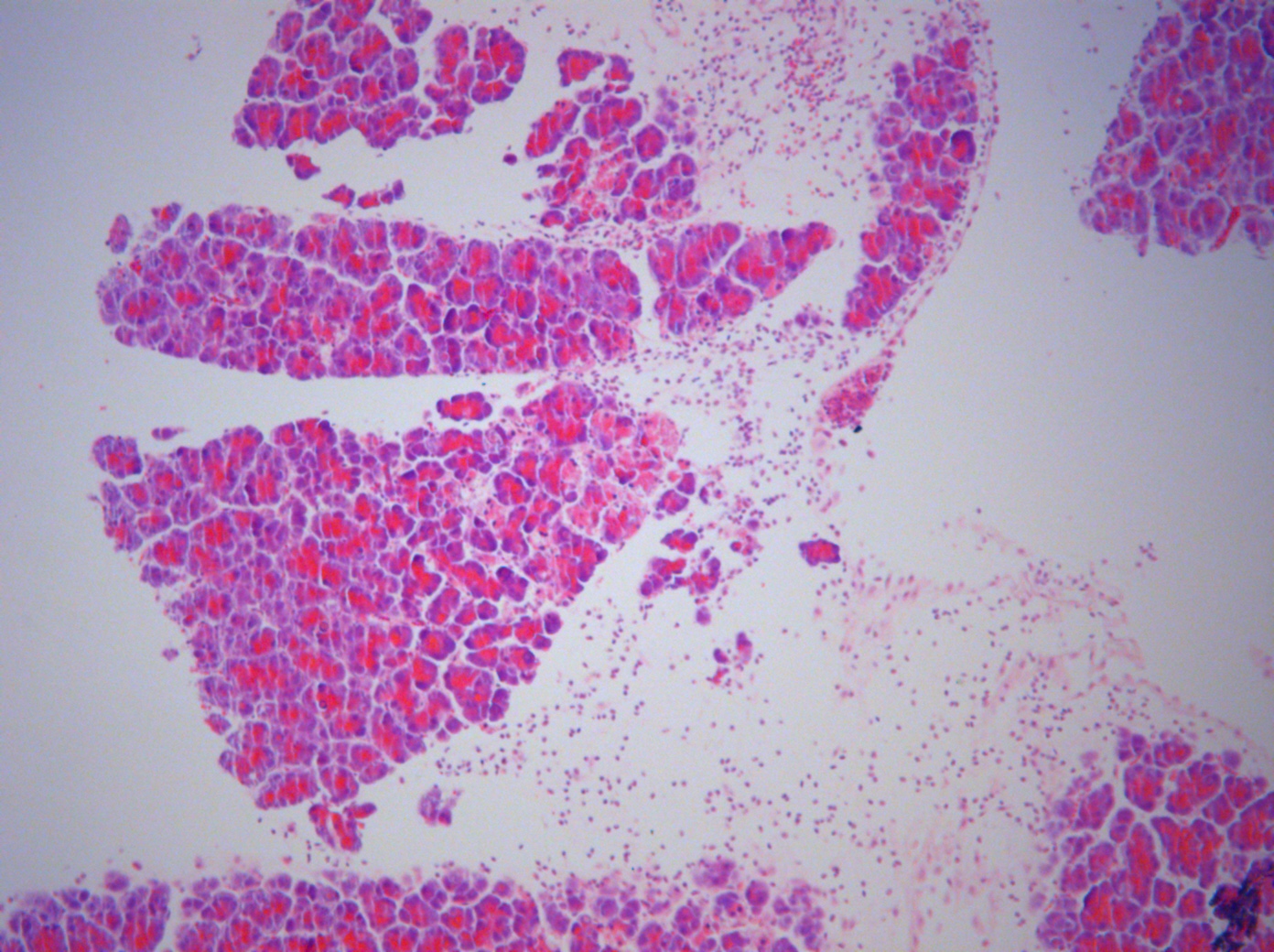

Supplement: Supplementary file 9 — Source data Fig. 9 [file 44318_2024_117_MOESM9_ESM.zip › Figure 9/9E/KO Cerulein AOAA.JPG]

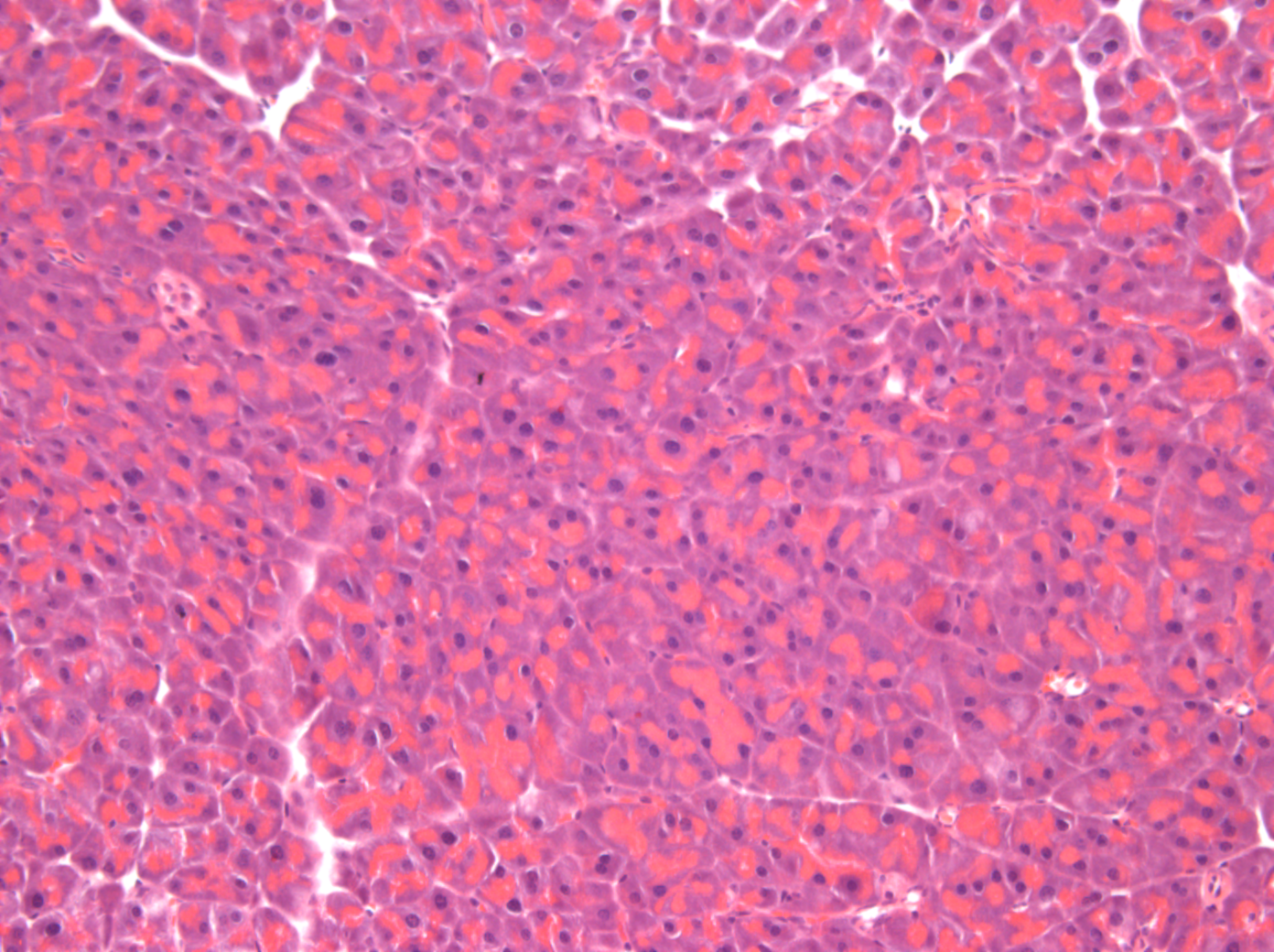

Supplement: Supplementary file 9 — Source data Fig. 9 [file 44318_2024_117_MOESM9_ESM.zip › Figure 9/9A/TRP14 KO Control.tif]

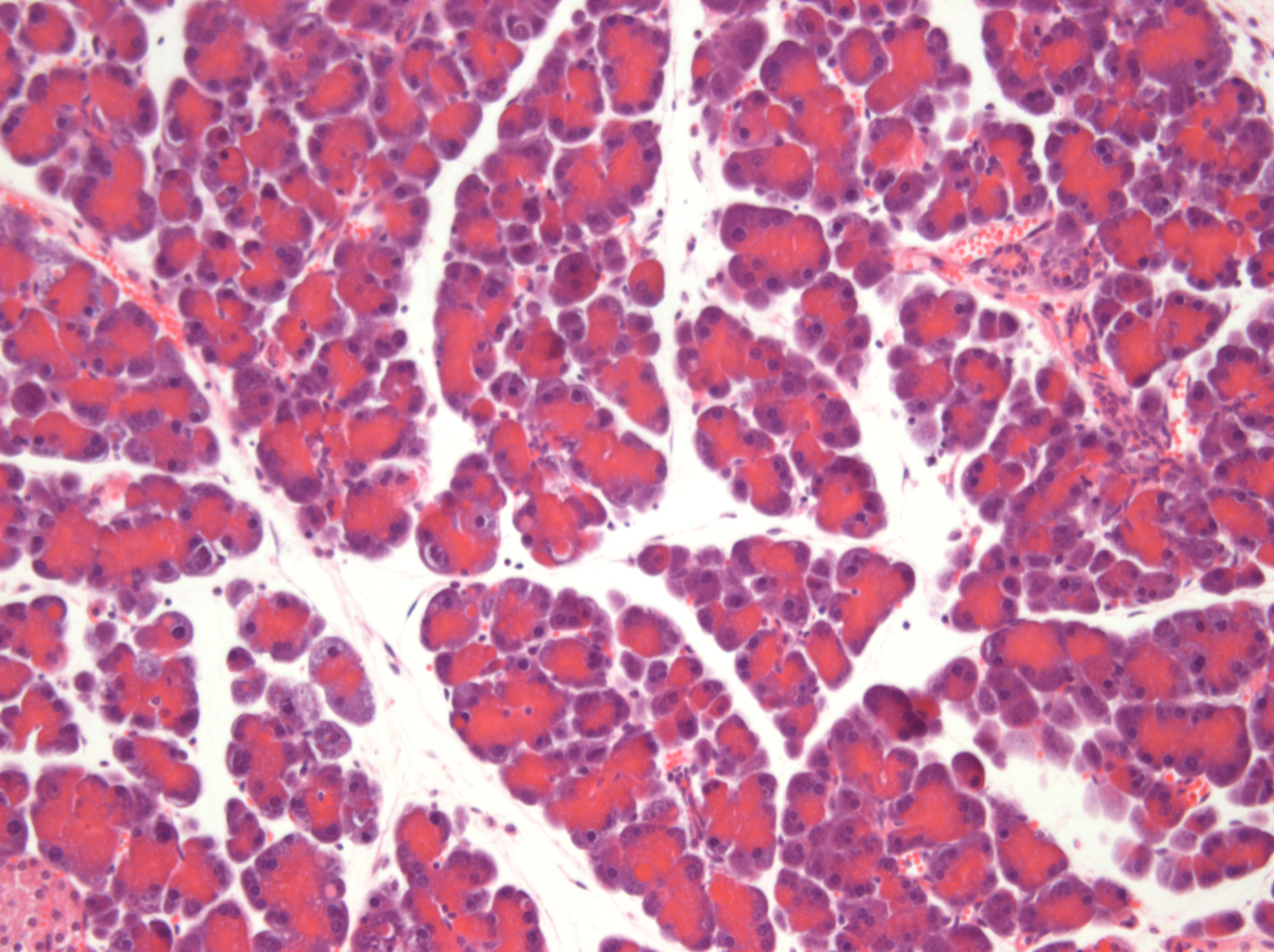

Supplement: Supplementary file 9 — Source data Fig. 9 [file 44318_2024_117_MOESM9_ESM.zip › Figure 9/9A/WT - Pancreatitis.tif]

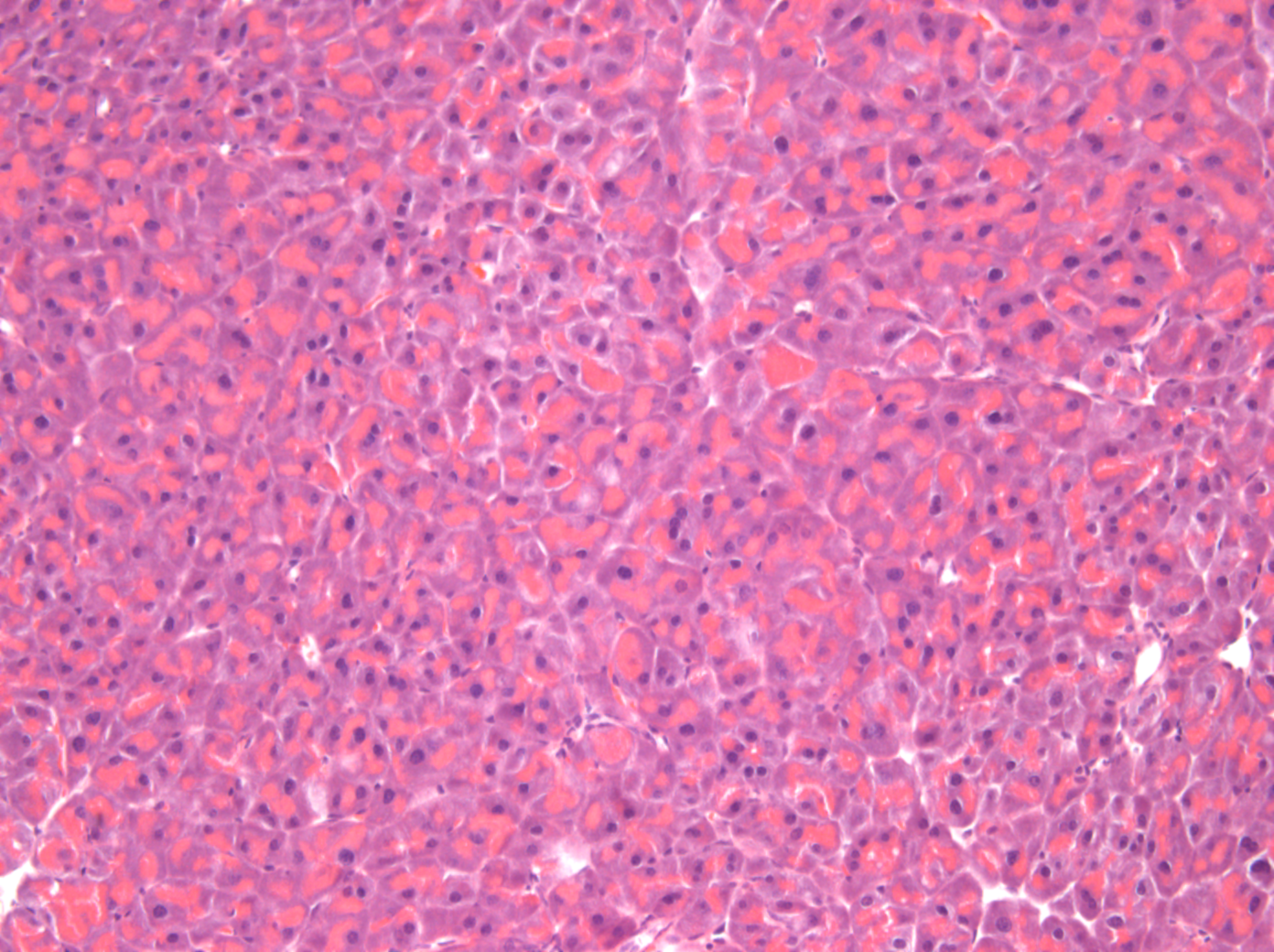

Supplement: Supplementary file 9 — Source data Fig. 9 [file 44318_2024_117_MOESM9_ESM.zip › Figure 9/9A/WT Control.tif]

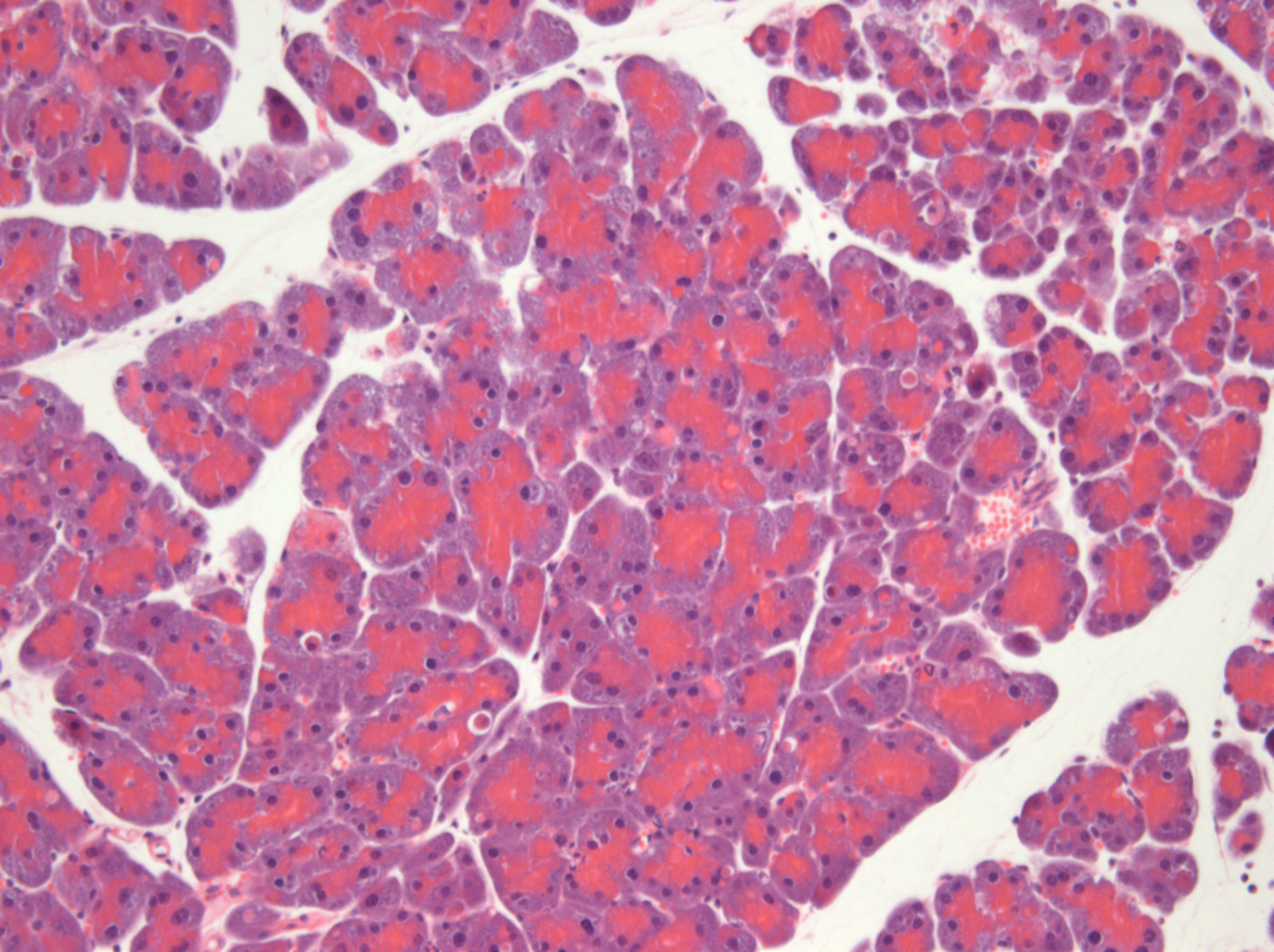

Supplement: Supplementary file 9 — Source data Fig. 9 [file 44318_2024_117_MOESM9_ESM.zip › Figure 9/9A/TRP14 KO Pancreatitis.tif]

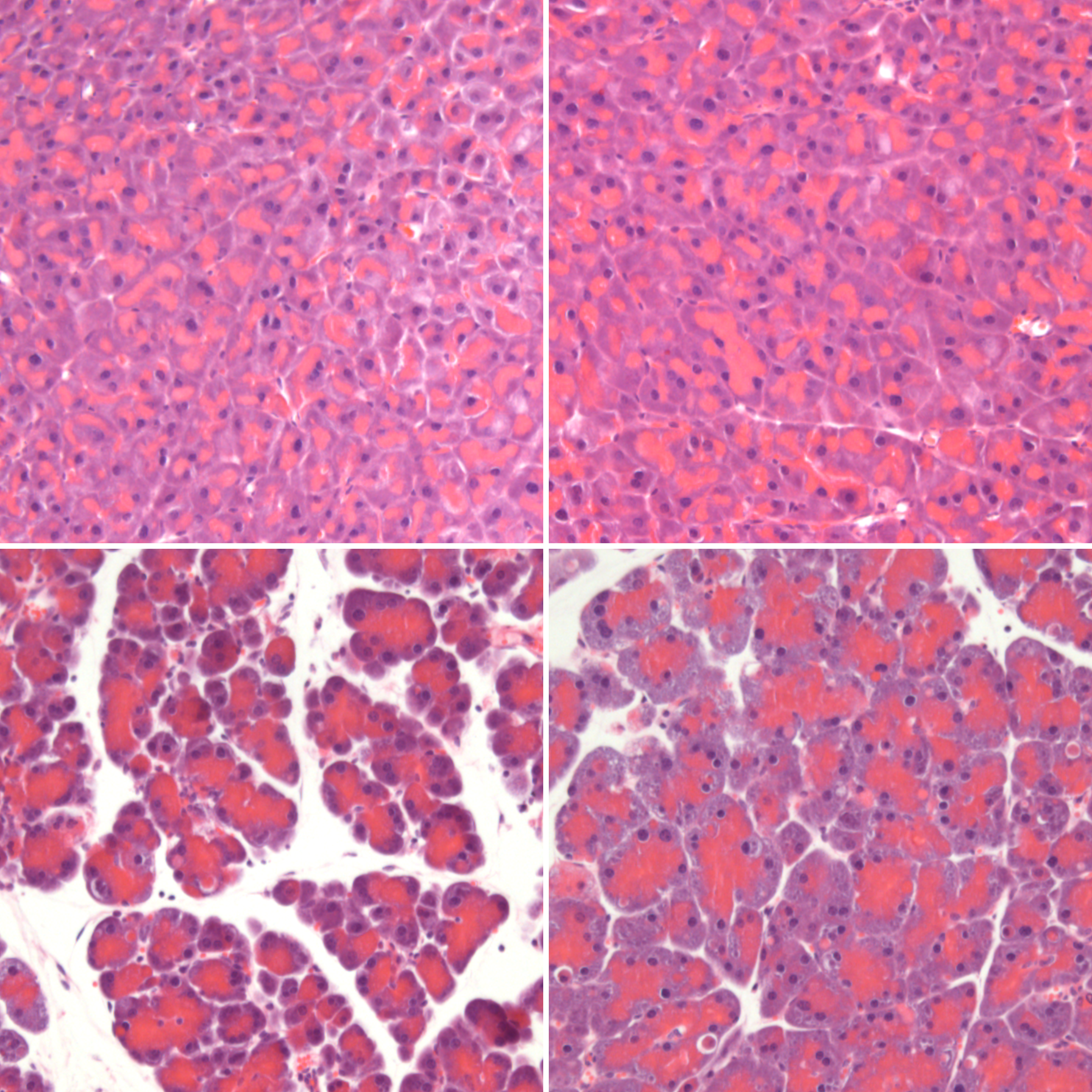

Supplement: Supplementary file 9 — Source data Fig. 9 [file 44318_2024_117_MOESM9_ESM.zip › Figure 9/9A/Figure 9A - H&E Control and Pancreatitis.tif]
