# Supplementary material for: TRP14 is the rate-limiting enzyme for intracellular cystine reduction and regulates proteome cysteinylation
Source: EMBO J. 2024 May 29;43(13):12. doi: 10.1038/s44318-024-00117-1 (PMC11217419; doi:10.1038/s44318-024-00117-1)
Supplement: Supplementary file 11 — Expanded View Figures [file 44318_2024_117_MOESM11_ESM.pdf]

Expanded View Figure

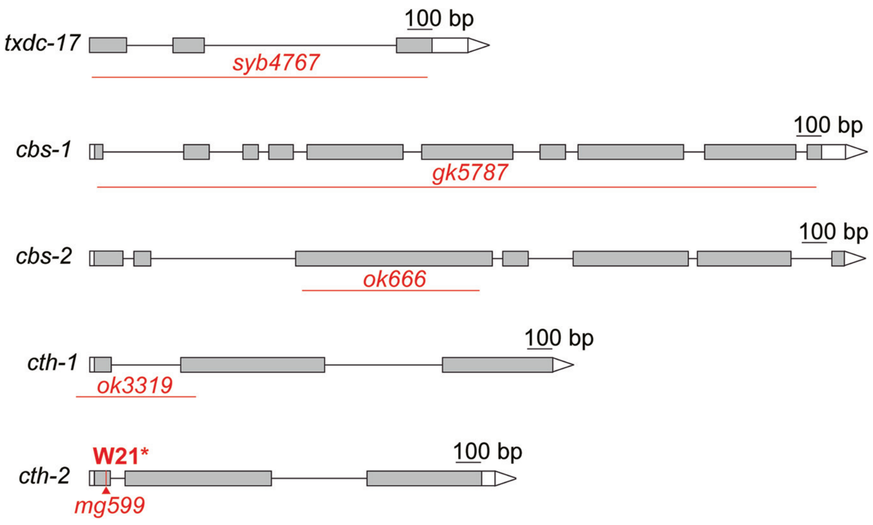

Figure EV1. Schematic representation of the genes and alleles in *C. elegans* used in this study.

Grey boxes indicate exons encoding the ORF and white boxes represent the UTRs. The molecular lesions are depicted in red.

 © The Author(s)
